# Crassifolins Q−W: Clerodane Diterpenoids From *Croton crassifolius* With Anti-Inflammatory and Anti-Angiogenesis Activities

**DOI:** 10.3389/fchem.2021.733350

**Published:** 2021-09-20

**Authors:** Canjie Li, Xin Sun, Wenjing Yin, Zhaochun Zhan, Qing Tang, Wenzhi Wang, Xuefang Zhuo, Zhongnan Wu, Haipeng Zhang, Yaolan Li, Yubo Zhang, Guocai Wang

**Affiliations:** ^1^ Guangdong Province Key Laboratory of Pharmacodynamic Constituents of TCM and New Drugs Research, Institute of Traditional Chinese Medicine and Natural Products, College of Pharmacy, Jinan University, Guangzhou, China; ^2^ The First Affiliated Hospital of Jinan University, Guangzhou, China; ^3^ Department of Pharmacology, School of Medicine, Guangdong Clinical Translational Center for Targeted Drug, Jinan University, Guangzhou, China

**Keywords:** Euphorbiaceae, *Croton crassifolius*, clerodane diterpenoids, anti-inflammatory activity, anti-angiogenic activity

## Abstract

Seven new clerodane diterpenoids, crassifolins Q−W (**1**–**7**), along with five known analogues (**8**–**12**), were isolated from the roots of *Croton crassifolius*. Their structures were identified by comprehensive spectroscopic analysis (UV, IR, NMR, and HR-ESI-MS), and their absolute configurations were determined by ECD spectra and X-ray crystallography. The activities of compounds **1**–**5** against inflammatory cytokines IL-6 and TNF-*α* levels on LPS-induced RAW 264.7 macrophages were assessed, and compound **5** showed the most significant activity with the secretion levels of IL-6 and TNF-*α* at 32.78 and 12.53%, respectively. Moreover, compounds **1**–**5** were screened for their anti-angiogenesis using a human umbilical vein endothelial cells *in vitro* mode; the results showed all of them exhibited obvious anti-angiogenesis activities, in particular, compound **5** showed the strongest anti-angiogenesis effect in the range of 6.25–50 μM.

## Introduction

The genus *Croton* (Euphorbiaceae) is a rich source of diterpenoids with interesting structural diversity such as clerodane-type (Campos et al., 2010; Khanitha and Damrong, 2011; Sun et al., 2014), kauritan-type (Dao et al., 2011; Suwancharoen et al., 2012), heliurane-type (Martha et al., 2011; Huang et al., 2014a), phorbol ester-type (Zhang et al., 2013; Corlay et al., 2014), and trachylobane-type (Martinsen et al., 2010). These diterpenoids exhibited remarkable biological characteristics including anti-inflammatory (Wang et al., 2015), anti-tumor (Fernandes et al., 2013; Young et al., 2013; Maslovskaya et al., 2015), anti-viral (Nina et al., 2014), anti-bacterial (Liu et al., 2014), and anti-oxidation activities (Mariana et al., 2013), which make them particularly valuable for phytochemical and pharmacological research studies. *Croton crassifolius* Geisel (Euphorbiaceae) is well known as “Jiguxiang” and mainly distributed in the south and southwest area of China, such as Guangxi, Guangdong, and Hainan provinces. The roots of the *C. crassifolius* are used as a traditional medicine for treatment of joint pain, rheumatic arthritis, stomach ache, pharyngitis, and jaundice (Boonyarathanakornkit et al., 1988). Previous phytochemical studies on *C. crassifolius* showed that the derivatives of diterpenoids were the main active ingredients (Huang et al., 2014b; Li et al., 2014).

In our early research studies, the investigations on *C. crassifolius* had led to the isolation of some new diterpenoids (Wang et al., 2012; Wang et al., 2016). However, the diterpenoids from their work showed relatively moderate biological activities including anti-HSV and anti-angiogenesis. In order to search more bioactive natural products from *Croton* plants, the phytochemical investigation on the roots of *C. crassifolius* was performed, leading to the isolation of seven new clerodane diterpenoids, named crassifolins Q−W (**1**–**7**), along with five known compounds, EBC-205 (**8**) (Maslovskaya et al., 2019), mallotucin B (**9**) (Takashi et al., 1976), crassin D (**10**) (Yuan et al., 2017), spiro [furan-3(2*H*),6'-(6*H*)naphtho (1,8-bc)furan]-2'a (2'*H*)-carboxylic acids (**11**) (Nakatsu et al., 1981; Li et al., 2019), and crassifolin H (**12**) (Qiu et al., 2016).

Considering the potential anti-inflammatory activity of clerodane diterpenoids (Zhang et al., 2019), we evaluated the inhibitory effects of diterpenoids **1**–**5** on inflammation by testing the secretion level of inflammatory cytokines IL-6 and TNF-*α* on LPS-stimulated RAW 264.7 cells. In earlier research papers, inflammation has anti-cancer effects, which can affect the host immune response, help the body recognize and kill cancer cells, and can be used in anti-cancer immunotherapy (Bryant, 1868). However, in recent years, more and more studies have shown that inflammation can promote the occurrence and development of cancer under most conditions and reduce the therapeutic effect of tumors (Francescone et al., 2014; Moses et al., 2018). Furthermore, the formation of new vessels and angiogenesis are important processes of tumor growth and metastasis. On this basis, we want to isolate compounds that can exhibit anti-inflammatory and anti-angiogenesis activities simultaneously, so as to act as lead compounds to interfere with inflammation and cancer. Moreover, a previous study (Wang et al., 2016) reported that some diterpenoids of this plant exhibited potential activity on anti-angiogenesis, which inspired us to conduct an anti-angiogenesis activity on compounds **1**–**5** using a human umbilical vein endothelial cell (HUVEC) *in vitro* mode. Herein, this article reported the isolation, structural elucidation, and evaluation of anti-inflammatory and anti-angiogenic activities of the new compounds.

## Results and Discussion

### Characterization of Compounds

**FIGURE 1:**
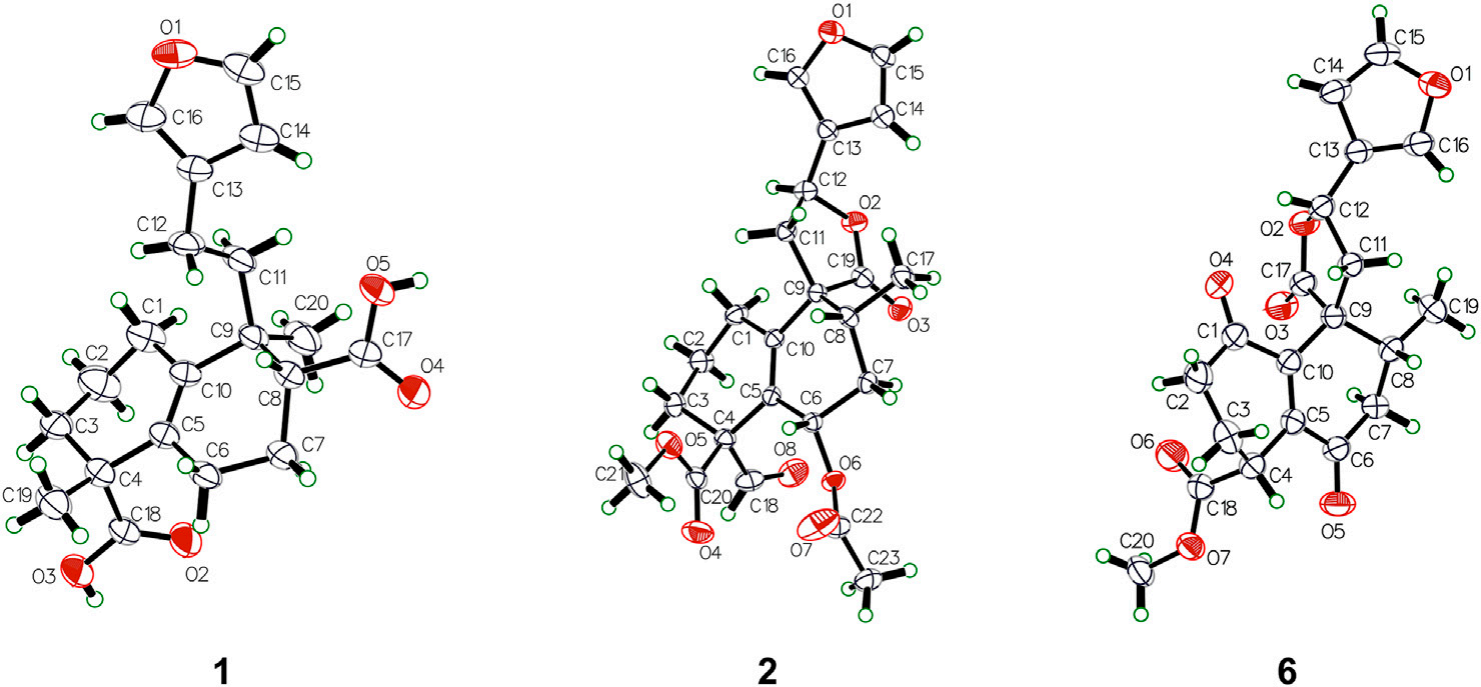
Key ^1^H–^1^H COSY and HMBC correlations of compounds **1**–**7**.

**FIGURE 2:**
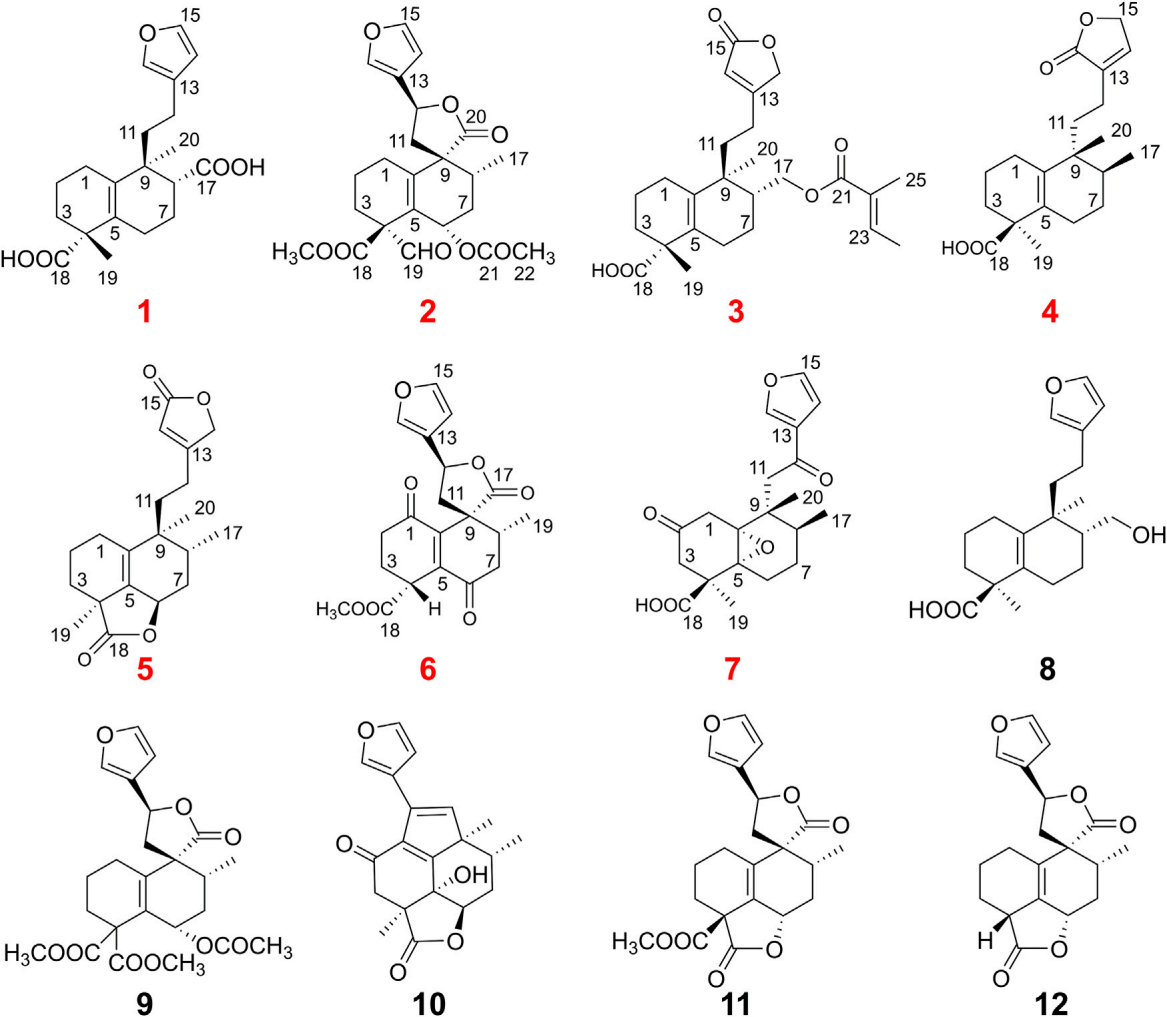
The X-ray ORTEP drawing of compounds **1**, **2**, and **6**.

**FIGURE 3:**
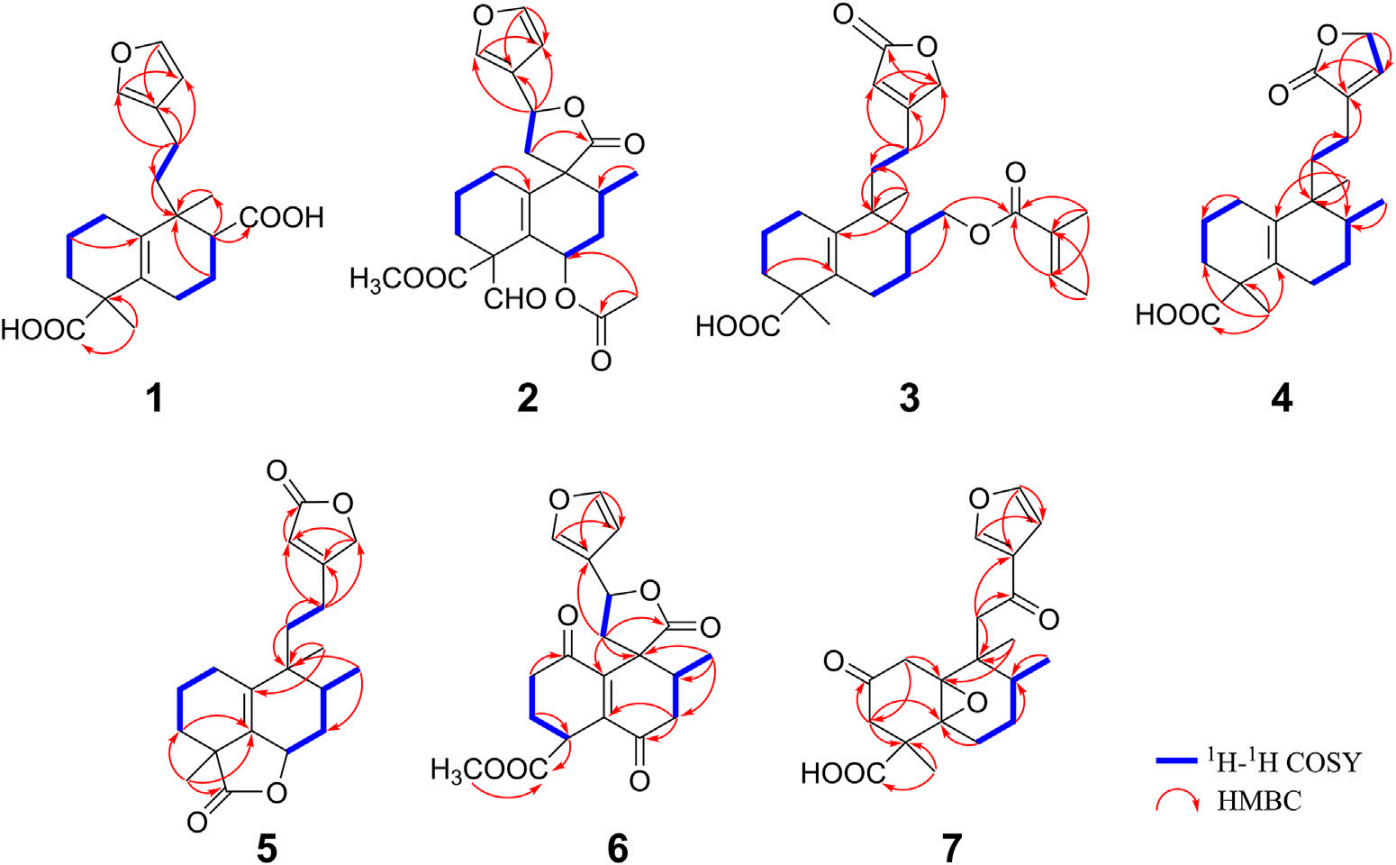
Structures of compounds **1**–**12**.

**FIGURE 4:**
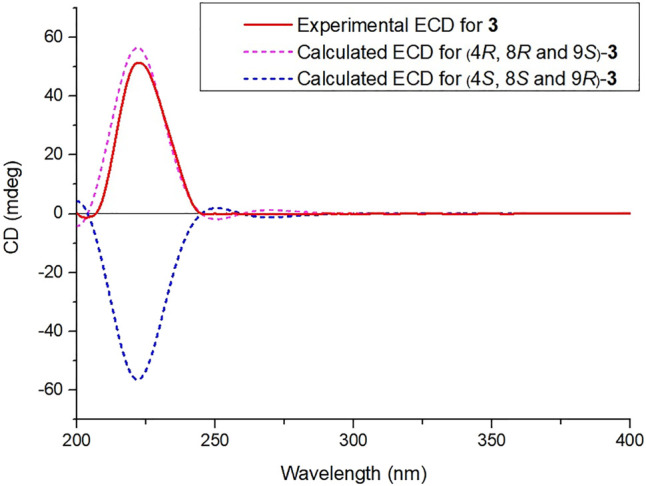
Calculated and experimental ECD spectra of compound **3**.

**FIGURE 5:**
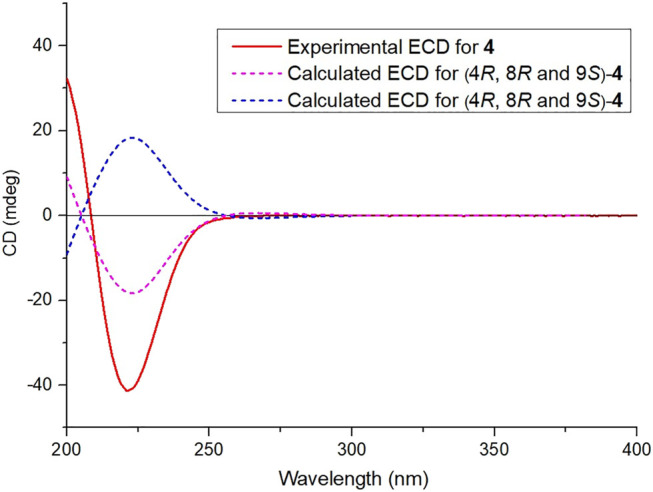
Calculated and experimental ECD spectra of compound **4**.

**FIGURE 6:**
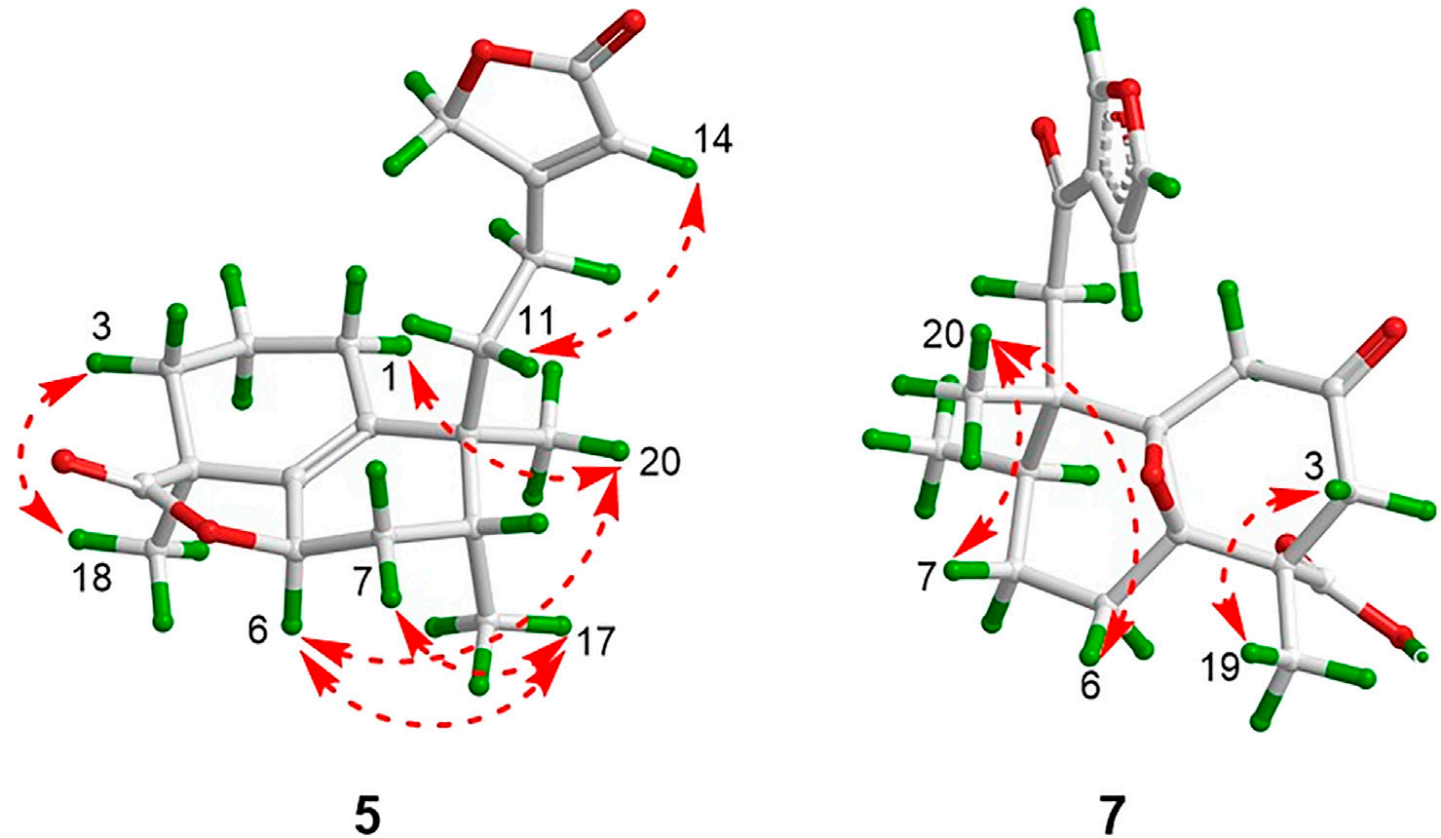
Key NOESY correlations of compounds **5** and **7**.

**FIGURE 7:**
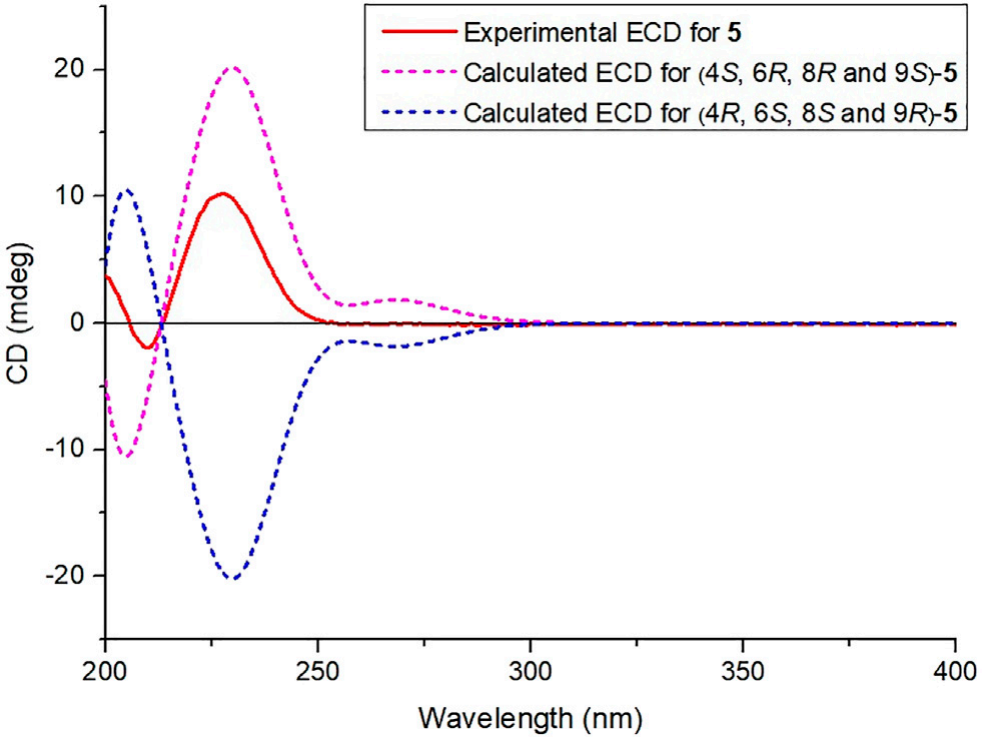
Calculated and experimental ECD spectra of compound **5**.

**FIGURE 8:**
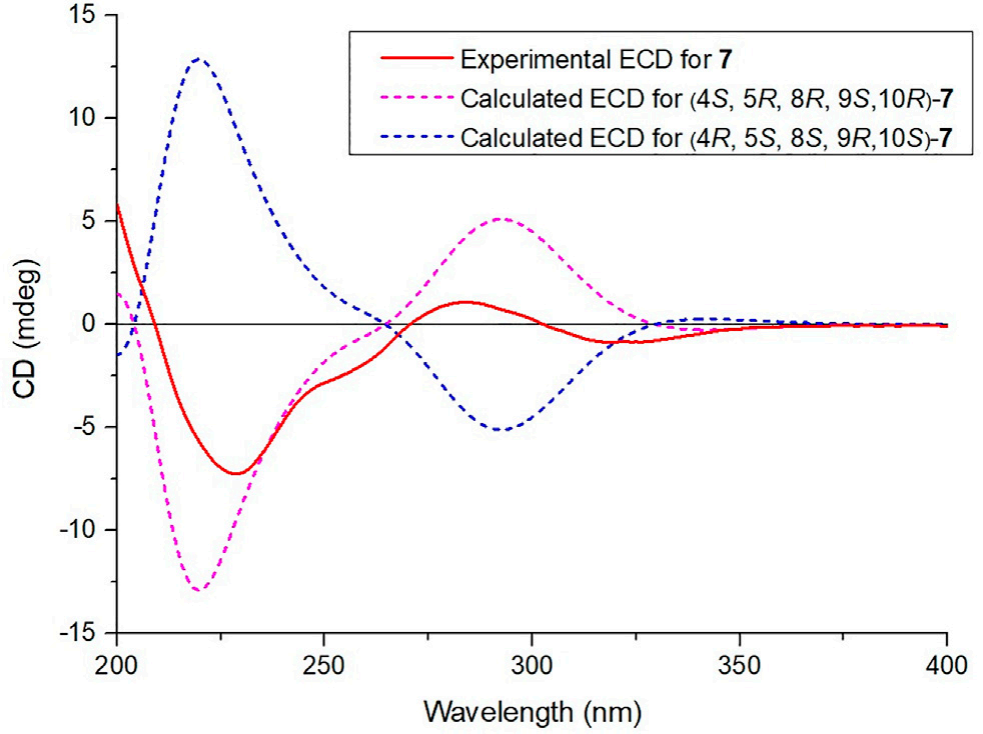
Calculated and experimental ECD spectra of compound **7**.

Compound **1** was isolated as a colorless crystal. Its molecular formula was deduced as C_20_H_26_O_5_ on the basis of its HR-ESI-MS data [*m/z* 369.1660 (M + Na)^+^, calcd for C_20_H_26_O_5_Na, 369.1672]. The IR spectrum suggested the presence of hydroxyl (3,463 cm^−1^), carbonyl (1710 cm^−1^), and furan ring (1,461, 867 cm^−1^) in the structure of **1**. The ^1^H NMR spectrum (Table 1) revealed the presence of three olefinic protons [*δ*_H_ 7.35 (1H, s), 7.22 (1H, s), and 6.29 (1H, s)] and two methyl groups [*δ*_H_ 1.30 (3H, s) and 1.12 (3H, s)]. The ^13^C NMR and DEPT spectra (Table 2) showed 20 carbons including two carboxyl carbons (*δ*_C_ 183.7 and 181.1), five quaternary carbons, seven methylenes, four methines, and two methyls, suggesting that **1** is a clerodane diterpenoid with a comparable structure to the known compound crassifolin B (Wang et al., 2012). The major differences between **1** and crassifolin B were the absence of a methyl (*δ*_C_ 16.3, C-17) and a *γ*-lactone ring, but the presence of a carboxylic acid group (*δ*_C_ 181.1) and a *β*-substituted furan ring, and the chemical shifts of C-13 to C-17 shifted from *δ*_C_ 171.2, 115.2, 174.2, 73.3, 16.3 to *δ*_C_ 125.3, 111.2, 142.9, 138.7, 181.1, respectively. These differences suggested that the *γ*-lactone in crassifolin B was replaced by a *β*-substituted furan ring in **1**, which was verified by the ^1^H–^1^H COSY correlation between H-14 (*δ*_H_ 6.29) and H-15 (*δ*_H_ 7.35) and the HMBC cross peaks from H-12 (*δ*_H_ 2.04) to C-13 (*δ*_C_ 125.3)/C-14 (*δ*_C_ 111.2)/C-16 (*δ*_C_ 138.7) and from H-16 (*δ*_H_ 7.22) to C-13/C-14/C-15 (Figure 1). In addition, the ^1^H–^1^H COSY correlations of H-6 (*δ*_H_ 1.47)/H-7 (*δ*_H_ 1.92) and H-7/H-8 (*δ*_H_ 2.70), along with the HMBC cross peaks from H-8 to C-9 (*δ*_C_ 40.9)/C-17 (*δ*_C_ 181.1)/C-20 (*δ*_C_ 23.0) were observed, which could confirm that the carboxylic acid group was attached to C-8 (Figure 1). Furthermore, to establish the stereochemistry of **1**, the single crystals were obtained in a chloroform system and subjected to an X-ray diffraction experiment with Cu K*α* radiation*.* The crystal data [Flack parameter -0.1 (3), hooft parameter -0.3 (3)] determined the absolute configuration of **1** as 4*R*, 8*R*, 9*S* (Figure 2). Consequently, the structure was determined and named as crassifolin Q. Compound **2** was obtained as a colorless crystal and the HR-ESI-MS showed an [M + Na]^+^ ion peak at *m/z* 453.1506 (calcd for C_23_H_26_O_8_Na, 453.1520), consistent with the molecular formula of C_23_H_26_O_8_. The ^1^H NMR spectrum (Table 1) showed apparent signals for an aldehyde proton [*δ*_H_ 9.89 (1H, s)], three olefinic protons [*δ*_H_ 7.62 (1H, s), 7.54 (1H, s), and 6.50 (1H, s)], two oxygenated methines [*δ*_H_ 5.48 (1H, t, *J* = 9.0 Hz) and 5.43 (1H, t, *J* = 5.1 Hz)], one oxygenated methyl [*δ*_H_ 3.68 (3H, s)], and two methyls [*δ*_H_ 1.92 (3H, s) and 1.05 (3H, d, *J* = 6.4 Hz)]. The ^13^C NMR and DEPT spectra (Table 2) exhibited the presence of 23 carbons, including one carbonyl carbon (*δ*_C_ 196.7), eight quaternary carbons, six methines, five methylenes, and three methyls, indicating that **2** was also a clerodane diterpenoid. The analysis of 1D NMR data showed that **2** had the analogous planar structure as that crassifolin M (Wang et al., 2016), except for the absence of a carbonyl group (*δ*_C_ 195.7, C-6), but the presence of an oxygenated methine (*δ*_C_ 71.7) and an acetoxyl group (*δ*_C_ 170.1 and 21.1) in **2** and the chemical shifts of C-5, C-6, and C-7 shifted from *δ*_C_ 133.2, 195.7, 40.9 to *δ*_C_ 129.8, 71.7, 32.7, correspondingly. In addition, the acetoxyl group was attached to C-6, which was confirmed by ^1^H–^1^H COSY correlation between H-6 (*δ*_H_ 5.46) and H-7 (*δ*_H_ 2.10), together with HMBC cross peaks from H-22 (*δ*_H_ 1.92) to C-6 (*δ*_C_ 71.7)/C-21 (*δ*_C_ 170.1) (Figure 1). Moreover, the absolute configuration of **2** was determined as 4*S*, 6*S*, 8*R*, 9*R*, and 12*S* by a single X-ray diffraction experiment (Figure 2). Therefore, the structure of two was confirmed as showed in Figure 3 and named as crassifolin R. Compound **3**, a yellow oil, which had the molecular formula C_25_H_34_O_6_ assigned by HR-ESI-MS (*m/z* 453.2234 [M + Na]^+^, calcd for C_25_H_34_O_6_Na, 453.2248). The IR spectrum indicated the presence of hydroxyl (3,419 cm^−1^) and ester carbonyl groups (1718, 1,259 cm^−1^). According to its ^1^H NMR spectrum (Table 1), compound **3** exhibited the presence of characteristic signals for two olefinic protons [*δ*_H_ 6.83 (1H, s, *J* = 7.0 Hz) and 5.82 (1H, s)], one methine [*δ*_H_ 1.78 (1H, m)], two oxygenated methylenes [*δ*_H_ 4.75 (2H, s), 4.38 (1H, dd, *J* = 11.0, 4.3 Hz), and 3.83 (1H, t, *J* = 9.2 Hz)], and four methyls [*δ*_H_ 1.78 (3H, s), 1.77 (3H, d, *J* = 7.0 Hz), 1.27 (3H, s), and 1.00 (3H, s)]. Comparison of 1D NMR data with those of crassifolin B (Wang et al., 2012) showed that they were similar to a typical clerodane diterpenoid skeleton. The differences between **3** and crassifolin B were the absence of a methyl (*δ*_C_ 16.2, C-17), but the presence of an oxygenated methylenes (*δ*_C_ 65.7) and a *cis*-2-methylbut-2-enoyloxy moiety (*δ*_C_ 168.4, 137.6, 128.8, 14.5, and 12.2). As a result, the chemical shifts of C-8 and C-17 shifted from *δ*_C_ 33.8 and 16.2 in crassifolin B to *δ*_C_ 39.0 and 65.7 in **3**, respectively. The presence of *cis*-2-methylbut-2-enoyloxy moiety was confirmed by the ^1^H–^1^H COSY correlation between H_3_-24 (*δ*_H_ 1.77) and H-23 (*δ*_H_ 6.83), together with the HMBC cross peaks from H_3_-24 to C-22 (*δ*_C_ 128.8)/C-23 (*δ*_C_ 137.6), from H_3_-25 (*δ*_H_ 1.78) to C-21 (*δ*_C_ 168.4)/C-22 (Figure 1). In addition, the HMBC cross peaks from H-17 (*δ*_H_ 3.83) to C-21 indicated that the *cis*-2-methylbut-2-enoyloxy moiety was connected to C-17 (*δ*_C_ 65.7). Due to the influence of substituents on the chemical shift of olefinic hydrogen, the chemical shift of olefinic proton in *cis*-2-methylbut-2-enoyloxy moiety was greater than that of *trans*-2-methylbut-2-enoyloxy moiety (Pedro et al., 1989; Russel et al., 2014), and our ^1^H NMR spectrum of **3** was consistent with *cis*-2-methylbut-2-enoyloxy moiety. Herein, the 2-methylbut-2-enoyloxy moiety in compound **3** was *cis*-configuration. Moreover, the absolute configuration of **3** was established by quantum-chemical ECD calculations. The experimental ECD spectrum of **3** showed a positive cotton effect at 224 nm (Δ*ε* +50.9), which was compared with the predicted ECD spectrum of (4*R*, 8*R*, 9*S*)-**3** and its enantiomer (Figure 4), indicating the absolute configuration of **3** was 4*R*, 8*R*, and 9*S*. Hence, the structure of **3** was established as Figure 3 and named as crassifolin S. Compound **4** was a white powder and showed an [M + Na]^+^ ion peak at *m/z* 355.1869 (calcd for C_20_H_28_O_4_Na, 355.1880) in the HR-ESI-MS spectrum, consistent with the molecular formula of C_20_H_28_O_4_. The ^1^H NMR spectrum of **4** (Table 1) exhibited one olefinic proton [*δ*_H_ 7.10 (1H, s)], one methine [*δ*_H_ 1.72 (1H, m)], and three methyls [*δ*_H_ 1.29 (3H, d, *J* = 6.8 Hz), 0.88 (3H, s), and 0.87 (3H, s)]. The NMR spectroscopic data of **4** resembled those of crassifolin B (Wang et al., 2012). A detailed comparison of the NMR spectra of **4** with crassifolin B indicated the connection position of C-12 and *γ*-lactone ring has changed, and the chemical shifts of C-12 to C-16 shifted from *δ*_C_ 23.7, 171.4, 115.1, 174.3, 73.3 to 20.5, 135.2, 174.5, 70.2, 143.9, respectively. The *γ*-lactone ring was confirmed by the ^1^H–^1^H COSY correlation between H-15 (*δ*_H_ 4.75) and H-16 (*δ*_H_ 7.10) along with the HMBC correlations from H-15 to C-13 (*δ*_C_ 135.2)/C-14 (*δ*_C_ 174.5)/C-16 (*δ*_C_ 143.9), from H-16 to C-14 (*δ*_C_ 174.5)/C-15 (*δ*_C_ 70.2). Moreover, the HMBC correlation from H-12 (*δ*_H_ 2.22) to C-13 was observed, which could confirm that the *γ*-lactone ring was attached to C-12 (Figure 1). The experimental ECD spectrum of **4** showed a positive cotton effect at 202 nm (Δ*ε* +35.0) and a negative cotton effect at 222 nm (Δ*ε* -42.1), which was similar to those of the calculated ECD spectrum for (4*R*, 8*R*, 9*S*)-**4** (Figure 5), suggested the absolute configuration of **4** is 4*R*, 8*R*, and 9*S*. Thus, compound **4** was named crassifolin T. Compound **5** was isolated as a yellow oil and the molecular formula was determined to be C_20_H_26_O_4_ by HR-ESI-MS (*m/z* 353.1710 [M + Na]^+^, calcd for C_20_H_24_O_6_Na, 353.1723). The IR spectrum showed characteristic ester carbonyl group (1743, 1,236 cm^−1^). The ^1^H NMR spectrum of **5** (Table 1) showed one olefinic proton [*δ*_H_ 5.82 (1H, s)], one oxygenated methine [*δ*_H_ 4.99 (1H, t, *J* = 8.1 Hz)], and three methyls [*δ*_H_ 1.32 (3H, s), 1.02 (3H, s), and 0.98 (3H, d, *J* = 7.2 Hz)]. The NMR spectra of **5** exhibited several similarities to crassifolin B (Wang et al., 2012). The main differences were the absence of a carboxylic acid group (*δ*_C_ 183.7, C-18) and a methylene (*δ*_C_ 27.8, C-6), but the presence of an ester group (*δ*_C_ 180.7) and a tertiary carbon bearing oxygen (*δ*_C_ 74.7), and the chemical shifts of C-4, C-6, and C-18 shifted from 47.8, 27.8, 183.7 to 41.7, 74.7, 180.7, respectively. This indicated that a lactone ring was formed between C-4 and C-6, which was confirmed by the ^1^H–^1^H COSY between H-6 (*δ*_H_ 4.99) and H-7 (*δ*_H_ 1.63) and the HMBC cross peak from H-19 (*δ*_H_ 1.32) to C-18. In addition, the key NOESY correlations between H-6 and H_3_-17 (*δ*_H_ 0.98)/H_3_-20 (*δ*_H_ 1.02), indicated that H-6 and H-17/H-20 were *α*-orientated (Figure 6). The experimental ECD spectrum of **5** exhibited a positive cotton effect at 226 nm (Δ*ε* +10.1) and a negative cotton effect at 206 nm (Δ*ε* -2.0), which was similar to those of the calculated ECD spectrum for (4*S*, 6*R*, 8*R*, 9*S*)-**5** (Figure 7). Thus, the structure of **5** was elucidated as 4*S*, 6*R*, 8*R*, 9*S* and compound **5** was named crassifolin U. Compound **6**, a white powder, which had an [M + H]^+^ ion peak at *m/z* 373.1283, consistent with the molecular formula C_20_H_20_O_7_. The ^1^H NMR spectrum of **6** (Table 1) showed three olefinic protons [*δ*_H_ 7.46 (1H, s), 7.44 (1H, s), and 6.40 (1H, s)], one oxygenated methine [*δ*_H_ 5.90 (t, *J* = 7.5 Hz)], and two methyls [*δ*_H_ 3.68 (s) and 1.14 (d, *J* = 7.0 Hz)]. The 1D NMR data of **6** were similar to those of spiro [furan-3(2*H*),1' (2'*H*)-naphtha-lene]-5'-carboxylic acid (Rodríguez and Jimeno, 2004), and the main differences were the absence of two methines [*δ*_C_ 50.2 (C-5) and 50.0 (C-10)] and a methylene (*δ*_C_ 28.1, C-1), but the presence of two olefinic carbons (*δ*_C_ 144.9 and 145.9) and a carbonyl group (*δ*_C_ 199.9). Thus, it indicated that two methines were replaced by two olefinic carbons and the methylene was replaced by a carbonyl group. These were confirmed by HMBC cross peaks from H-2 (*δ*_H_ 2.56) to C-1 (*δ*_C_ 199.9), from H-7 (*δ*_H_ 2.44) to C-5 (*δ*_C_ 144.9), and from H-11 (*δ*_H_ 2.71) to C-10 (*δ*_C_ 145.9) (Figure 1). The single crystal were obtained in a methanol system and subjected to an X-ray diffraction experiment with Cu K*α* radiation and the crystal data [Flack parameter -0.06 (3), hooft parameter -0.03 (3)] determined the absolute configuration of **6** is 4*, 8*, 9*R*, 12*S* (Figure 1). Compound **6** was named crassifolin V. Compound **7** was obtained as yellow oil and had the molecular formula C_20_H_24_O_6_. The ^1^H NMR spectrum of **7** (Table 1) exhibited three olefinic protons [*δ*_H_ 8.05 (1H, s), 7.41 (1H, s), and 6.75 (1H, s)], one methine [*δ*_H_ 1.97 (m)], and three methyls [*δ*_H_ 1.20 (s), 1.08 (s) and 0.99 (d, *J* = 6.5 Hz)]. According to the 1D NMR spectroscopic data, the planar structure of compound **7** showed several similarities to methyl 5a,10a-epoxy-2,12-dioxo-13 (16),14-enthalimandien18-oate (Marcos et al., 2003), and the main differences were the absence of a carbethoxy group, but the presence of a carboxylic acid group (*δ*_C_ 176.6, C-18). Thus, it indicated the carbethoxy group was replaced by a carboxylic acid group in **7**, which was supported by the HMBC cross peaks from H-3 (*δ*_H_ 2.70) to C-4 (*δ*_C_ 51.0) and from H_3_-19 (*δ*_H_ 1.08) to C-4/C-5/C-18 (Figure 1). Moreover, the NOESY correlation between H-8 and H_3_-20 was not observed, which suggested that H_3_-20 and H_3_-17 were *α*-orientated; besides, the correlations of H_3_-20/H-1*α*, of H-1*α*/H-3*α*, and of H-3*α*/H_3_-19 indicated that H_3_-20 and H_3_-19 were *α*-orientated (Figure 6). The experimental ECD spectrum of **7** showed a positive cotton effect at 281 nm (Δ*ε* +2.1) and a negative cotton effect at 226 nm (Δ*ε* -7.0), which was similar to those of the calculated ECD spectrum for (4*S*, 5*R*, 8*R*, 9*S*, 10*R*)-**7** (Figure 8), suggested the absolute configuration of **7** is 4*S*, 5*R*, 8*R*, 9*S*, 10*R*. Compound **7** was named crassifolin W.

**TABLE 1:** ^1^H NMR data of **1**–**7** (*δ* in ppm, *J* in Hz)^a^.

Position	1	2	3	4	5	6	7
1	1.84, m	α 2.34, m	1.65, m	1.95, m	2.06, m	—	α 3.09, d (17.1)
	—	β 1.96, m	—	—	—	—	β 2.66, m
2	α 2.14, m	1.70, m	1.61, m	α 1.71, m	1.34, m	2.56, m	—
	β 1.85, m	—	—	β 1.51, m	—	—	—
3	α 2.07, m	α 2.09, m	α 2.03, m	1.94, m	α 1.83, m	α 2.43, m	α 2.70, m
	β 1.45, m	β 1.80, m	β 1.42, m	—	β 1.35,m	β 2.17, m	β 2.26, d (17.1)
4	—	—	—	—	—	3.97, t (4.6)	—
6	α 1.67, m	5.46, t (8.2)	α 2.07, m	1.96, m	4.99, t (8.1)	—	α 1.75, m
	β 1.47, m	—	β 1.97, m	—	—	—	β 1.68, d (9.6)
7	α 1.92, m	α 2.10, m	α 1.73, m	α 1.50, m	α 2.09, m	α 2.90, dd (16.3, 10.7)	α 1.68, d (9.6)
	β 1.70, m	β 1.87, m	β 1.46, m	β 1.35, m	β 1.63, m	β 2.44, m	β 1.42, m
8	2.70, dd (10.2, 4.3)	1.89, m	1.78, m	1.72, m	1.97, m	2.22, m	1.97, m
11	α 2.04, m	α 2.87, dd (14.5, 8.7)	1.74, m	1.62, m	1.69, m	α 2.71, dd (12.3, 9.0)	2.79, s
	β 1.92, m	β 2.34, m	—	—	—	β 2.18, m	—
12	α 2.54, t (13.9)	5.46, t (8.2)	α 2.41, m	α 2.22, m	2.31, m	5.90, t (7.5)	—
	β 2.01, m		β 1.98, m	β 2.01, m	—	—	—
14	6.29, s	6.50, s	5.82, s	—	5.82, s	6.40, s	6.75, s
15	7.35, s	7.54, s	—	4.75, d (1.8)	—	7.44, s	8.05, s
16	7.22, s	7.62, s	4.75, s	7.10, s	4.72, s	7.46, s	7.41, s
17	—	1.05, d (6.4)	α 4.38, dd (11.0, 4.3)	0.88, d (6.8)	α 0.98, d (7.2)		0.99, d (6.5)
	—	—	β 3.83, t (9.2)	—	β 1.32, s	—	—
18	—	—	—	—	—	—	—
19	1.30, s	9.89, s	1.27, s	1.29, s		1.14, d (7.0)	1.08, s
20	1.12, s		1.00, s	0.87, s	1.02, s	—	1.20, s
22	—	1.92, s	—	—	—	—	—
23	—	—	6.83, d (7.0)	—	—	—	—
24	—	—	1.77, d (7.0)	—	—	—	—
25	—	—	1.78, s	—	—	—	—
18-OCH_3_	—	3.68, s	—	—	—	3.68, s	—

a All measured in CDCl3 at 400 MHz.

**TABLE 2:** ^13^C NMR data of **1**–**7** (*δ* in ppm)^a^.

Position	1	2	3	4	5	6	7
1	21.7, CH2	26.1, CH2	24.8, CH2	25.2, CH2	22.8, CH2	199.9, C	43.7, CH2
2	24.9, CH2	17.6, CH2	20.0, CH2	19.6, CH2	18.5, CH2	35.4, CH2	205.7, C
3	36.6, CH2	28.7, CH2	36.6, CH2	35.6, CH2	29.2, CH2	24.4, CH2	45.9, CH2
4	47.7, C	59.2, C	47.6, C	47.5, C	41.7, C	38.7, CH	51.0, C
5	131.8, C	129.8, C	133.2, C	131.4, C	131.6, C	144.9, C	76.6, C
6	20.1, CH2	71.7, CH	27.5, CH2	25.9, CH2	74.7, CH	198.6, C	32.8, CH2
7	37.4, CH2	32.7, CH2	22.2, CH2	26.8, CH2	31.3, CH2	41.7, CH2	24.9, CH2
8	46.5, CH	36.2, CH	39.0, CH	33.4, CH	32.7, CH	37.9, CH	35.8, CH
9	40.9, C	54.1, C	40.5, C	40.9, C	39.4, C	48.8, C	44.0, C
10	135.3, C	138.2, C	134.6, C	135.8, C	136.2, C	145.9, C	89.0, C
11	27.1, CH2	40.5, CH2	33.9, CH2	34.4, CH2	36.9, CH2	41.6, CH2	44.4, CH2
12	19.7, CH2	72.6, CH	23.7, CH2	20.5, CH2	24.2, CH2	72.7, CH	194.4, C
13	125.3, C	125.0, C	171.0, C	135.2, C	170.1, C	125.4, C	129.7, C
14	111.2, CH	108.1, CH	115.3, CH	174.5, CH	115.6, CH	108.3, CH	108.9, CH
15	142.9, CH	144.4, CH	174.2, C	70.2, CH2	173.9, C	144.3, CH	147.7, CH
16	138.7, CH	139.6, CH	73.3, CH2	143.9, CH	73.1, CH2	139.2, CH	144.5, CH
17	181.1, C	16.6, CH3	65.7, CH2	16.1, CH3	15.6, CH3	175.2, C	17.5, CH_3_
18	183.7, C	172.2, C	182.7, C	182.7, C	180.7, C	171.3, C	176.6, C
19	24.4, CH3	196.7, CH	24.4, CH3	23.1, CH3	21.0, CH3	16.0, CH3	14.0, CH_3_
20	23.0, CH3	176.7, C	21.4, CH3	21.0, CH3	22.2, CH3	—	15.3, CH_3_
21	—	170.1, C	168.4, C	—	—	—	—
22	—	21.1, CH3	128.8, C	—	—	—	—
23	—	—	137.6, CH	—	—	—	—
24	—	—	14.5, CH3	—	—	—	—
25	—	—	12.2, CH3	—	—	—	—
18-OCH_3_	—	52.6, CH3	—	—	—	52.9, CH3	—

a All measured in CDCl3 at 100 MHz.

### The Hypothetical Biosynthesis Pathway of New Compounds

**FIGURE 9:**
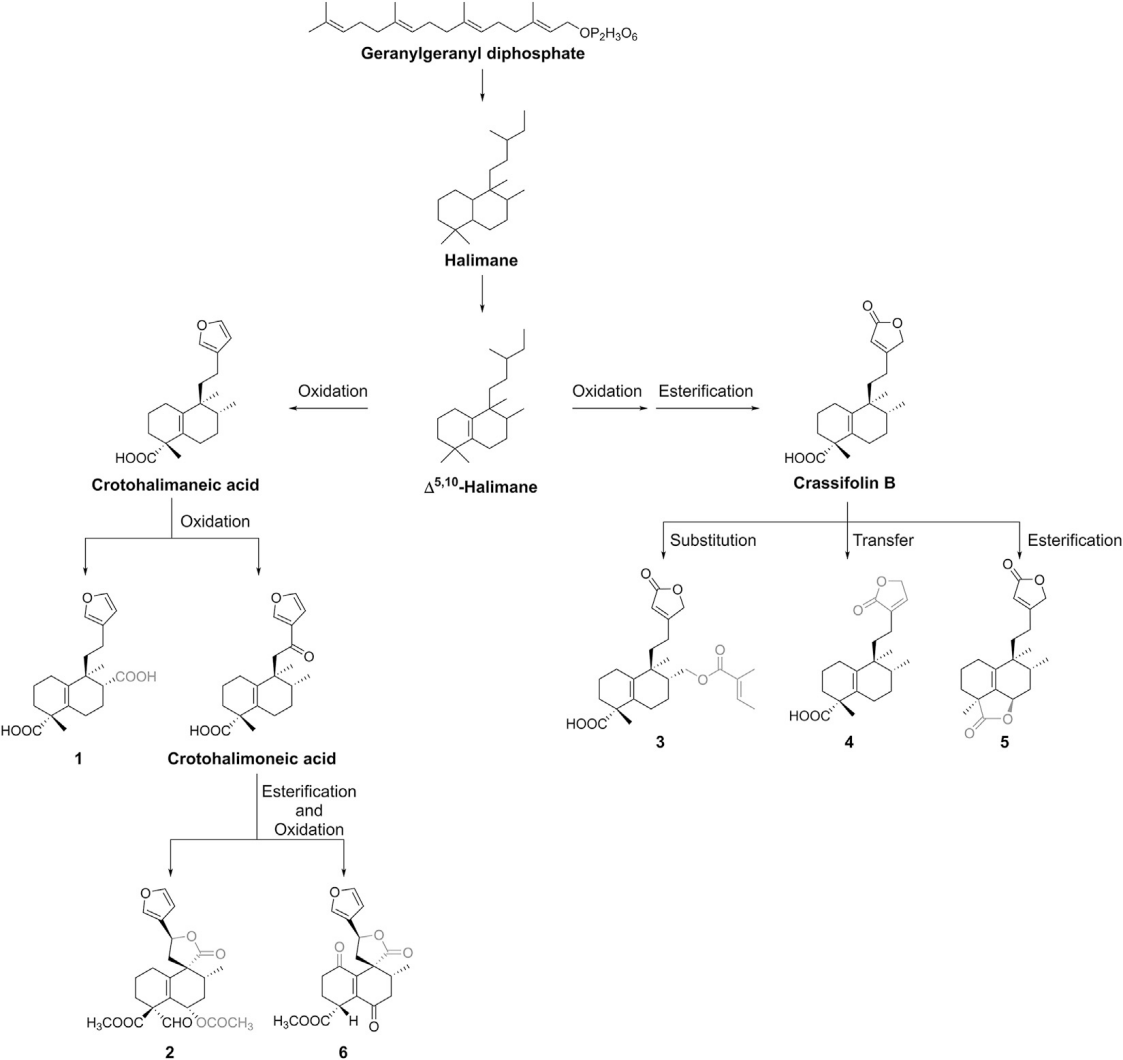
Hypothetical biosynthesis pathway of new diterpenoids.

In this paper, the new compounds we isolated from *C. crassifolius* all have the clerodane skeleton, which inspired us deriving their possible biosynthetic pathways. Biogenetically, geranylgeranyl diphosphate is the precursor of diterpenoids, and it converts to the halimane system first (Figure 9). The carbocations participate in methyl migration, thereby generates some unsaturated halimanes represented by ∆^5,10^-halimane (Maslovskaya et al., 2019). In this process, ∆^5,10^-halimane underwent oxidation to produce crassifolin B, or underwent oxidation and esterification to gain crotohalimaneic acid. On the one side, crassifolin B underwent substitution, transfer, and esterification to afford crassifolin S (**3**), crassifolin T (**4**), and crassifolin U (**5**), respectively. On the other side, crotohalimaneic acid underwent oxidation to obtain crassifolin Q (**1**) and crotohalimoneic acid. Crotohalimoneic acid underwent esterification and oxidation to get crassifolin R (**2**) and crassifolin V (**6**). However, the stereochemistry of C-18 and C-19 of compound **7** and **8** were different for other isolated compounds, which implied their skeleton were different from others.

### Anti-Inflammatory Activities of Compounds 1–5

According to previous reports (Wang et al., 2015; Maslovskaya et al., 2019), clerodane diterpenoids of *Croton crassifolius* had showed significant anti-inflammatory effect. Therefore, compounds (**1**–**5**) were tested for their activities against inflammatory cytokines IL-6 and TNF-*α* on RAW 264.7 cells. The cytotoxicities of compounds **1**–**5** were assessed by MTT assay on RAW 264.7 cells. As a result, compounds **1**–**5** at the concentration of 50 μM had no obvious cytotoxic activities on RAW 264.7 cells after 24 h treatment. In this study, ELISA was applied to evaluate the levels of two inflammatory cytokines IL-6 and TNF-*α*. As shown in Table 3, compounds **1**–**5** showed the secretion levels of IL-6 ranging from 32.78 to 77.88%, and the TNF-*α* ranging from 12.53 to 89.38%. Among them, compound **5** showed the most significant secretion levels of IL-6 and TNF-*α* at 32.78 and 12.53%, respectively.

**TABLE 3:** Anti-inflammatory activities of compounds **1**–**5** in LPS-stimulated RAW 264.7 cells.

Group	IL-6 (%)	TNF-*α* (%)
Blank	1.99 ± 2.89	1.68 ± 3.15
LPS	100 ± 2.23	100 ± 4.38
Dexamethasone	16.76 ± 2.98^a^	48.26 ± 3.68^a^
1	72.23 ± 3.84^b^	89.38 ± 3.54^b^
2	77.88 ± 5.21^b^	77.73 ± 4.22^b^
3	73.36 ± 1.06^b^	79.23 ± 3.21^b^
4	35.48 ± 2.15^a^	54.14 ± 1.55^a^
5	32.78 ± 1.99^a^	12.53 ± 1.92^a^

a *p* < 0.001.

b *p* < 0.01 *vs* LPS group.

### Anti-Angiogenesis Activities of Compounds 1–5

**FIGURE 10:**
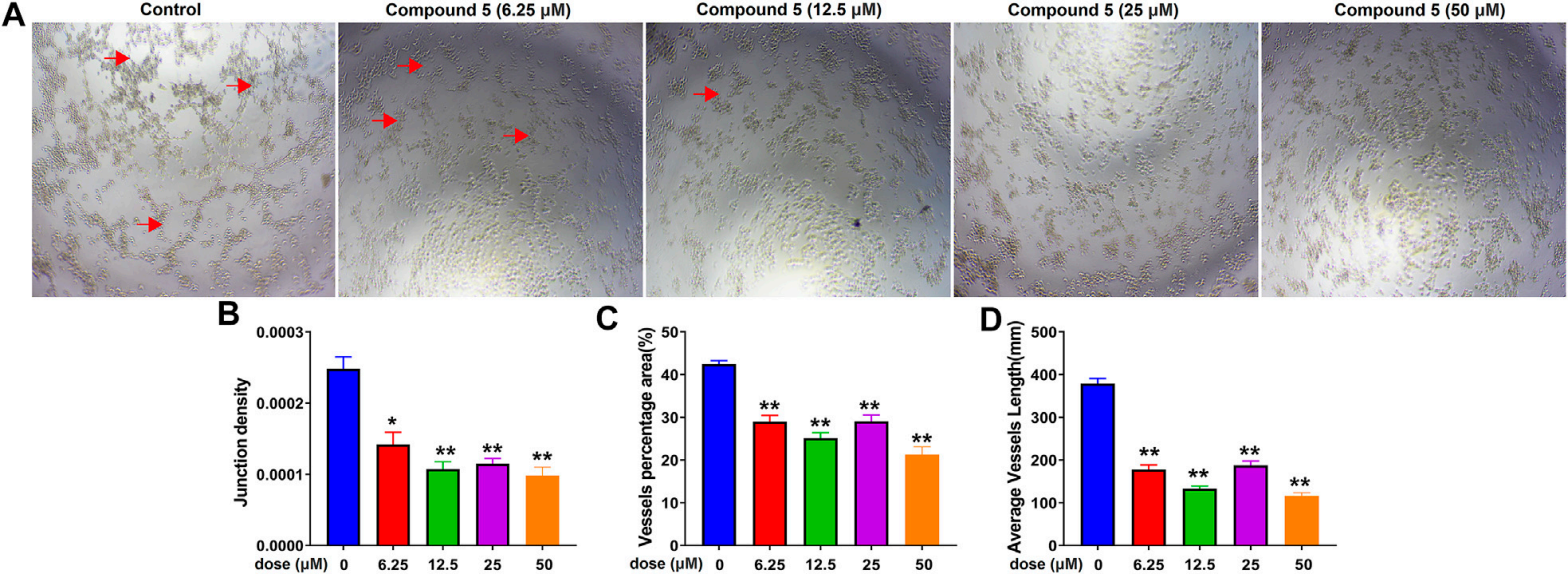
The inhibitory effect of compound **5** on angiogenesis. **(A)** Bright field images of HUVECs seeded on Matrigel for 24 h after treatment at different doses of compound **5**; red arrows indicated the cavities formation during angiogenesis. **(B)**, **(C)**, **(D)** Quantitative analysis on junction densities, vessel percentage areas, and average vessel lengths, respectively. Data are expressed as mean ± SD of three independent experiments. ^*^ *p* < 0.05, ^**^ *p* < 0.005, ^***^ *p* < 0.001 compared with the control group.

Considering the potential anti-angiogenesis activity of diterpenoids from *C. crassifolius* (Huang et al., 2016; Wang et al., 2016), compounds **1**–**5** were evaluated for their anti-angiogenesis activities by using tube formation assay in HUVECs. In this assay, we evaluated anti-angiogenesis activities of compounds according to junction densities, vessel percentage areas, and average vessel lengths. Experimental results (Figure 10 and Supplementary Figure S71−Figure S74) showed that compounds **1**–**5** significantly inhibited angiogenesis by reducing the number of cavities formation. Among these compounds, compound **5** showed the strongest anti-angiogenesis effect, resulting in half maximal inhibitory concentration (IC_50_) values of junction densities, vessel percentage areas, and average vessel lengths of 7.20 ± 2.23, 48.27 ± 1.98, and 8.62 ± 2.89 μM, respectively. These results revealed that compounds **1**–**5** significantly inhibited the tube formation of HUVECs and showed their anti-angiogenesis activities. According to the results of experiments with biological activities, crassifolin U (**5**) showed better inhibitory activities in both inflammation and angiogenesis. Compared with other compounds, there were more lactone rings in the structure of compound **5**, which implied that the lactone ring may be an important functional group for anti-inflammatory and anti-angiogenesis activities.

## Conclusion

In conclusion, seven new clerodane diterpenoids were isolated from the roots of *C. crassifolius*. Their structures were identified by comprehensive analysis of spectroscopic data and X-ray crystallography. The anti-inflammatory activities of compounds **1**–**5** was determined according to two inflammatory cytokines IL-6 and TNF-*α*, and compound **5** showed the strongest anti-inflammatory ability with the secretion of IL-6 and TNF-*α* at 32.78 and 12.78%, respectively. Moreover, the results of experiments on anti-angiogenesis activities showed compounds **1**–**5** inhibited angiogenesis obviously and compound **5** showed the strongest anti-angiogenesis effect with doses in the range of 6.25–50 μM.

## Experimental

### General Experimental Procedures

UV spectra were measured by a JASCO V-550 UV/VIS spectrophotometer (JASCO, Tokyo, Japan). IR spectra were measured by a JASCO FT/IR-480 plus FT-IR spectrometer with KBr pellets (JASCO, Tokyo, Japan). A JASCO P-1020 polarimeter was used for measuring the optical rotations (JASCO, Tokyo, Japan). HRESIMS data were accomplished using an Agilent 6210 ESI/TOF mass spectrometer (Agilent, Massachusetts, United States). ECD spectra were measured by an Applied Photophysics Chirascan plus CD (Applied Photophysics, United Kingdom). NMR spectra were obtained on a Bruker AV-400 spectrometer with TMS as the internal standard (Bruker, Karlsruhe, Germany), and the chemical shifts (*δ*) are expressed in ppm and coupling constants (*J*) in Hz. For column chromatography (CC), silica gel (200–300 mesh, Qingdao Marine Chemical Plant, Qingdao, P. R. China), ODS (50 μm, YMC, Kyoto, Japan), and Sephadex LH-20 (Pharmacia Biotech, Uppsala, Sweden) were used. Silica gel GF_254_ plates (Yantai Chemical Industry Research Institute, Yantai, China) were used for thin-layer chromatography (TLC). HPLC separations were performed using a COSMOSIL C_18_ preparative column (5 μm, 20 × 250 mm). All chemical reagents were purchased from Shandong Yuwang Chemical Company (Shandong, P. R. China).

### Plant Material

The roots of *C. crassifolius* were collected from Yulin City, Guangxi province of China. The species was identified by Professor Guangxiong Zhou of Jinan University. A voucher specimen (No. 20200713) is deposited in the Institute of Traditional Chinese Medicine and Natural Products of Jinan University.

### Extraction and Isolation

The air-dried roots of *C. crassifolius* (20.0 kg) were extracted three times with 95% ethanol at room temperature. The solution was evaporated under reduced pressure to get a residue (1,500.0 g), which was suspended in water and then partitioned with petroleum ether (PE, 55.6 g), dichloromethane (CH_2_Cl_2_, 1,005.3 g), and ethyl acetate (EtOAc, 180.9 g). The dichloromethane extract was chromatographed on a silica gel column chromatograph using the petroleum ether/ethyl acetate (100:0→0:100, v/v) solvent system. The fractions were examined by TLC and combined to give eight fractions (Fr. 1−Fr. 8). Fr. 6 (72.4 g) was subjected to a reversed silica gel column chromatography eluting with MeOH/H_2_O (30:70→100:0, v/v) to afford six subfractions (Fr. 6A−Fr. 6F). Fr. 6C (6.1 g) was separated by Sephadex LH-20 (CHCl_3_/MeOH, 1:1, v/v) and further purified by the preparative HPLC (MeOH/H_2_O, 70:30, v/v) to yield compounds **1** (20.2 mg), **2** (26.8 mg), **3** (23.6 mg), and **8** (19.8 mg). F. 6D (10.2 g) was purified by Sephadex LH-20 (CHCl_3_/MeOH, 1:1, v/v) to obtain compounds **4** (232.6 mg), **5** (22.4 mg), **6** (230.8 mg), **9** (332.5 mg), and **10** (33.8 mg). Fr. 6E (5.5 g) was separated by the preparative HPLC (MeOH/H_2_O, 60:40, v/v) to afford compound **7** (30.7 mg), **10** (35.5 mg), **11** (19.7 mg), and **12** (22.9 mg).

### Characterization of Compounds 1–7

Crassifolin Q (**1**): colorless crystal (MeOH); mp 139–140°C; [*α*]^25^_D_ +36.8 (*c* 1.0, MeOH); HR-ESI-MS *m/z* 369.1660 [M + Na]^+^ (calcd for C_19_H_24_O_7_Na, 369.1672); UV (MeOH) *λ*_max_ 200 nm; IR (KBr) *ν*_max_ 3,463, 2,956, 1710, 1,461, 1,355, 1,203, 1,037 cm^−1^; ^1^H and ^13^C NMR data see Tables 1, 2. Crassifolin R (**2**): colorless crystal (MeOH); mp 146–147°C; [*α*]^25^_D_ +57.9 (*c* 1.0, MeOH); HR-ESI-MS *m/z* 453.1506 [M + Na]^+^ (calcd for C_19_H_24_O_7_Na, 453.1520); UV (MeOH) *λ*_max_ 201 nm; IR (KBr) *ν*_max_ 3,133, 2,965, 1737, 1,450, 1,249, 1,164, 1,018, 808, 748, 603 cm^−1^; ^1^H and ^13^C NMR data see Tables 1, 2. Crassifolin S (**3**): yellow oil; [*α*]^25^_D_ +33.9 (*c* 1.0, MeOH); HR-ESI-MS *m/z* 453.2234 [M + Na]^+^ (calcd for C_19_H_24_O_7_Na, 453.2248); UV (MeOH) *λ*_max_ 203 nm; IR (KBr) *ν*_max_ 3,419, 2,942, 1718, 1,446, 1,357, 1,259, 1,155, 1,027, 867 cm^−1^; ECD (MeOH) *λ*_max_ (Δ*ε*) 224 (+50.9) nm; ^1^H and ^13^C NMR data see Tables 1, 2. Crassifolin T (**4**): white powder; mp 135–136°C; [*α*]^25^_D_ -48.6 (*c* 1.0, MeOH); HR-ESI-MS *m/z* 355.1869 [M + Na]^+^ (calcd for C_19_H_24_O_7_Na, 355.1880); UV (MeOH) *λ*_max_ 201 nm; IR (KBr) *ν*_max_ 2,946, 2,657, 1735, 1,448, 1,280, 1,195, 1,056, 923, 842 cm^−1^; ECD (MeOH) *λ*_max_ (Δ*ε*) 201 (+35.0), 224 (−42.1) nm; ^1^H and ^13^C NMR data see Tables 1, 2. Crassifolin U (**5**): yellow oil; (*α*)^25^_D_ +42.5 (*c* 1.0, MeOH); HR-ESI-MS *m/z* 353.1710 (M + Na)^+^ (calcd for C_19_H_24_O_7_Na, 353.1723); UV (MeOH) *λ*_max_ 202 nm; IR (KBr) *ν*_max_ 2,935, 1743, 1,639, 1,448, 1,236, 1,164, 1,014, 869 cm^−1^; ECD (MeOH) *λ*_max_ (Δ*ε*) 226 (+10.1), 206 (−2.0) nm; ^1^H and ^13^C NMR data see Tables 1, 2. Crassifolin V (**6**): white powder; mp 144–147°C; [*α*]^25^_D_ +22.7 (*c* 1.0, MeOH); HR-ESI-MS *m/z* 373.1283 [M + H]^+^ (calcd for C_20_H_21_O_7_, 373.1282); UV (MeOH) *λ*_max_ 203 nm; IR (KBr) *ν*_max_ 2,948, 2,898, 1745, 1,679, 1,434, 1,201, 1,160, 1,025 cm^−1^; ECD (MeOH) *λ*_max_ (Δ*ε*) 263 (+42.5), 225 (−28.1) nm; ^1^H and ^13^C NMR data see Tables 1, 2. Crassifolin W (**7**): yellow oil; (*α*)^25^_D_ -32.5 (*c* 1.0, MeOH); HR-ESI- MS *m/z* 361.1644 [M + H]^+^ (calcd for C_20_H_25_O_6_, 361.1646); UV (MeOH) *λ*_max_ 200 nm; IR (KBr) *ν*_max_ 3,436, 2,942, 2,879, 1783, 1720, 1,666, 1,562, 1,511, 1,390, 1,253, 1,155, 871 cm^−1^; ECD (MeOH) *λ*_max_ (Δ*ε*) 280 (+2.1), 226 (−7.0) nm; ^1^H and ^13^C NMR data see Tables 1, 2.

### X-Ray Crystallographic Data of 1, 2, and 6

Crystal data for **1**: C_20_H_26_O_5_, *M* = 346.41, orthorhombic, space group *P2*_*1*_ *2*_*1*_ *2*_*1*_, *a* = 9.129 (5) Å, *b* = 24.185 (5) Å, *c* = 17.080 (5) Å, *V* = 3,771.66 (2) Å^3^, *Z* = 8, *d*_*x*_ = 1.220 g/cm^3^, *F* (000) = 1,488.0, *μ*(Cu K*α*) = 0.708 mm^−1^. Data collection was performed on a Gemini S Ultra using graphite monochromatic radiation (*λ* = 1.54184 Å), 2,188 unique reflections were collected to *θ*_max_ = 74.98°, where 3,898 reflections were observed [*F* ^2^ > 2*σ*(*F* ^2^)]. The structure was solved by direct methods (SHELXS 97) and refined by full matrix least-squares on *F* ^2^. Final *R* = 0.0525, *R*_w_ = 0.1726, and *S* = 1.046. Crystallographic data for this structure have been deposited with the Cambridge Crystallographic Data Center as CCDC 2078339 for crassifolin Q (**1**). Crystal data for **2**: C_23_H_26_O_8_, *M* = 430.44, orthorhombic, space group *P2*_*1*_ *2*_*1*_ *2*_*1*_, *a* = 8.2304 (1) Å, *b* = 10.0512 (2) Å, *c* = 25.6929 (4) Å, *V* = 2,125.46 (6) Å^3^, *Z* = 4, *d*_*x*_ = 1.345 g/cm^3^, *F* (000) = 912.0, *μ*(Cu K*α*) = 0.850 mm^−1^. Data collection was performed on a Gemini S Ultra using graphite monochromatic radiation (*λ* = 1.54184 Å), 2,479 unique reflections were collected to *θ*_max_ = 73.93°, where 4,308 reflections were observed [*F* ^2^ > 2*σ*(*F* ^2^)]. The structure was solved by direct methods (SHELXS 97) and refined by full matrix least-squares on *F* ^2^. Final *R* = 0.0357, *R*_w_ = 0.1174, and *S* = 1.026. Crystallographic data for this structure have been deposited with the Cambridge Crystallographic Data Center as CCDC 2078342 for crassifolin R (**2**). Crystal data for **6**: C_20_H_21_O_7_, *M* = 372.12, orthorhombic, space group *P2*_*1*_ *2*_*1*_ *2*_*1*_, *a* = 6.3217 (4) Å, *b* = 7.3898 (8) Å, *c* = 10.1209 (7) Å, *V* = 440.11 (7) Å^3^, *Z* = 1, *d*_*x*_ = 1.405 g/cm^3^, *F* (000) = 196.0, *μ*(Cu K*α*) = 0.895 mm^−1^. Data collection was performed on a Gemini S Ultra using graphite monochromatic radiation (*λ* = 1.54184 Å), 2,998 unique reflections were collected to *θ*_max_ = 74.455°, where 3,600 reflections were observed [*F* ^2^ > 2*σ*(*F* ^2^)]. The structure was solved by direct methods (SHELXS 97) and refined by full matrix least-squares on *F* ^2^. Final *R* = 0.0488, *R*_w_ = 0.1459, and *S* = 1.108. Crystallographic data for this structure have been deposited with the Cambridge Crystallographic Data Center as CCDC 2093010 for crassifolin V (**6**).

### Cell Viability Assay

RAW 264.7 cells were purchased from American Type Culture Collection. Cells were seeded in a 96-well plate at the density of 5 × 10^4^ cells/ml for 24 h and then the cells were treated with compounds **1**–**5** for 24 h. The mixture was then removed and each well of the plates was incubated with 30 μl of MTT (5 mg/ml) at 37°C for 4 h. After complete removal of the supernatant, DMSO (100 μl/well) was added into the plates to dissolve the formazan produced in the cells. The absorbance was recorded by a microplate reader at 570 nm.

### Assay of Enzyme-Linked Immunosorbent Assay

To quantify the level of proinflammatory cytokines, RAW 264.7 cells were placed in a 24-well plate at a density of 2 × 10^5^/ml for 24 h. Then, the cells were pretreated with 50 μM of isolated compounds **1**–**5** for 12 h before stimulation with LPS for another 12 h. The level of IL-6 and TNF-*α* in cell supernatants were quantified with ELISA kits according to the manufacturer's protocols. In this assay, data were expressed as mean ± SD. Statistical significance was considered when *p* value <0.05.

### Tube Formation Assay

The effects of compounds on angiogenesis of endothelial cell *in vitro* were tested using the tube formation assay on Matrigel (BD Biosciences, MA, United States). In this assay, Matrigel (50 μl) was added to the wells of a pre-chilled 96-well plate and put at 37°C for 30 min to congeal. The prepared HUVECs with a density of 2 × 10^5^ cells/well was added and treated with isolated compounds **1**–**5** at different concentrations. The image of tube formation was collected after 8 h. The junction densities, vessel percentage areas, and average vessel lengths were measured and recorded. In this assay, data were expressed as mean ± SD of three independent experiments. ^*^ *p* < 0.05, ^**^ *p* < 0.01, ^***^ *p* < 0.001 compared with the control group.

## Data Availability

The original contributions presented in the study are included in the article/Supplementary files, further inquiries can be directed to the corresponding authors.
