# Supplementary material for: Crassifolins Q−W: Clerodane Diterpenoids From Croton crassifolius With Anti-Inflammatory and Anti-Angiogenesis Activities
Source: Front Chem. 2021 Sep 20;9:733350. doi: 10.3389/fchem.2021.733350 (PMC8488372; doi:10.3389/fchem.2021.733350)
Supplement: Supplementary file 1 [file DataSheet1.pdf]

Supplementary Material

Crassifolins Q−W: Clerodane Diterpenoids from *Croton crassifolius* with Anti-inflammatory and Anti-angiogenesis Activities

Canjie Li[1†], Xin Sun[1,2†], Wenjing Yin[3], Zhaochun Zhan[1], Qing Tang[1], Wenzhi Wang[1], Xuefang Zhuo[1], Zhongnan Wu[2], Haipeng Zhang[3], Yaolan Li[1*], Yubo Zhang[1,3*] and Guocai Wang[1*]

[1] Institute of Traditional Chinese Medicine & Natural Products, Guangdong Province Key Laboratory of Pharmacodynamic Constituents of TCM and New Drugs Research, College of Pharmacy, Jinan University, Guangzhou 510632, China
[2] The First Affiliated Hospital of Jinan University, Guangzhou 510632, China
[3] Guangdong Clinical Translational Center for Targeted Drug, Department of Pharmacology, School of Medicine, Jinan University, Guangzhou 510632, China

[*] **Correspondence:**
Yaolan Li: tliyl@jnu.edu.cn; Yubo Zhang: ybzhang99@126.com;
Guocai Wang: twangguocai@jnu.edu.cn.

List of supplementary material

Contents: **page**

Figure S1. HR-ESI-MS of **1**........ 1
Figure S2. UV spectrum of **1** (CH_3OH)........ 1
Figure S3. IR spectrum of **1** (KBr disc)........ 2
Figure S4. ^{1}H NMR spectrum of **1** in $CDCl_3$ (400 MHz)........ 2
Figure S5. ^{13}C NMR spectrum of **1** in $CDCl_3$ (100 MHz)........ 3
Figure S6. DEPT-135 spectrum of **1** in $CDCl_3$ (100 MHz)........ 3
Figure S7. ^{1}H-^{1}H COSY spectrum of **1** in $CDCl_3$........ 4
Figure S8. HSQC spectrum of **1** in $CDCl_3$........ 4
Figure S9. HMBC spectrum of **1** in $CDCl_3$........ 5
Figure S10. NOESY spectrum of **1** in $CDCl_3$........ 5
Figure S11. HR-ESI-MS of **2**........ 6
Figure S12. UV spectrum of **2** (CH_3OH)........ 6
Figure S13. IR spectrum of **2** (KBr disc)........ 7
Figure S14. ^{1}H NMR spectrum of **2** in $CDCl_3$ (400 MHz)........ 7
Figure S15. ^{13}C NMR spectrum of **2** in $CDCl_3$ (100 MHz)........ 8
Figure S16. DEPT-135 spectrum of **2** in $CDCl_3$ (100 MHz)........ 8
Figure S17. ^{1}H-^{1}H COSY spectrum of **2** in $CDCl_3$........ 9
Figure S18. HSQC spectrum of **2** in $CDCl_3$........ 9
Figure S19. HMBC spectrum of **2** in $CDCl_3$........ 10
Figure S20. NOESY spectrum of **2** in $CDCl_3$........ 10
Figure S21. HR-ESI-MS of **3**........ 11
Figure S22. UV spectrum of **3** (CH_3OH)........ 11
Figure S23. IR spectrum of **3** (KBr disc)........ 12
Figure S24. ^{1}H NMR spectrum of **3** in $CDCl_3$ (400 MHz)........ 12
Figure S25. ^{13}C NMR spectrum of **3** in $CDCl_3$ (100 MHz)........ 13
Figure S26. DEPT-135 spectrum of **3** in $CDCl_3$ (100 MHz)........ 13
Figure S27. ^{1}H-^{1}H COSY spectrum of **3** in $CDCl_3$........ 14
Figure S28. HSQC spectrum of **3** in $CDCl_3$........ 14
Figure S29. HMBC spectrum of **3** in $CDCl_3$........ 15
Figure S30. NOESY spectrum of **3** in $CDCl_3$........ 15
Figure S31. HR-ESI-MS of **4**........ 16
Figure S32. UV spectrum of **4** (CH_3OH)........ 16
Figure S33. IR spectrum of **4** (KBr disc)........ 17
Figure S34. ^{1}H NMR spectrum of **4** in $CDCl_3$ (400 MHz)........ 17
Figure S35. ^{13}C NMR spectrum of **4** in $CDCl_3$ (100 MHz)........ 18
Figure S36. DEPT-135 spectrum of **4** in $CDCl_3$ (100MHz)........ 18
Figure S37. ^{1}H-^{1}H COSY spectrum of **4** in $CDCl_3$........ 19
Figure S38. HSQC spectrum of **4** in $CDCl_3$........ 19
Figure S39. HMBC spectrum of **4** in $CDCl_3$........ 20
Figure S40. NOESY spectrum of **4** in $CDCl_3$........ 20
Figure S41. HR-ESI-MS of **5**........ 21
Figure S42. UV spectrum of **5** (CH_3OH)........ 21

Figure S43. IR spectrum of **5** (KBr disc)......22
Figure S44. ^{1}H NMR spectrum of **5** in $CDCl_3$ (400 MHz)......22
Figure S45. ^{13}C NMR spectrum of **5** in $CDCl_3$ (100 MHz)......23
Figure S46. DEPT-135 spectrum of **5** in $CDCl_3$ (100 MHz)......23
Figure S47. ^{1}H-^{1}H COSY spectrum of **5** in $CDCl_3$......24
Figure S48. HSQC spectrum of **5** in $CDCl_3$......24
Figure S49. HMBC spectrum of **5** in $CDCl_3$......25
Figure S50. NOESY spectrum of **5** in $CDCl_3$......25
Figure S51. HR-ESI-MS of **6**......26
Figure S52. UV spectrum of **6** (CH_3OH)......26
Figure S53. IR spectrum of **6** (KBr disc)......27
Figure S54. ^{1}H NMR spectrum of **6** in $CDCl_3$ (400 MHz)......27
Figure S55. ^{13}C NMR spectrum of **6** in $CDCl_3$ (100 MHz)......27
Figure S56. DEPT-135 spectrum of **6** in $CDCl_3$ (100 MHz)......28
Figure S57. ^{1}H-^{1}H COSY spectrum of **6** in $CDCl_3$......28
Figure S58. HSQC spectrum of **6** in $CDCl_3$......29
Figure S59. HMBC spectrum of **6** in $CDCl_3$......29
Figure S60. NOESY spectrum of **6** in $CDCl_3$......30
Figure S61. HR-ESI-MS of **7**......30
Figure S62. UV spectrum of **7** (CH_3OH)......31
Figure S63. IR spectrum of **7** (KBr disc)......31
Figure S64. ^{1}H NMR spectrum of **7** in $CDCl_3$ (400 MHz)......32
Figure S65. ^{13}C NMR spectrum of **7** in $CDCl_3$ (100 MHz)......32
Figure S66. DEPT-135 spectrum of **7** in $CDCl_3$ (100 MHz)......33
Figure S67. ^{1}H-^{1}H COSY spectrum of **7** in $CDCl_3$......33
Figure S68. HSQC spectrum of **7** in $CDCl_3$......34
Figure S69. HMBC spectrum of **7** in $CDCl_3$......34
Figure S70. NOESY spectrum of **7** in $CDCl_3$......35
Figure S71. The inhibitory effect of compound **1** on angiogenesis......35
Figure S72. The inhibitory effect of compound **2** on angiogenesis......36
Figure S73. The inhibitory effect of compound **3** on angiogenesis......36
Figure S74. The inhibitory effect of compound **4** on angiogenesis......36
Figure S75. ^{1}H NMR spectrum of **8** in $CDCl_3$ (400MHz)......37
Figure S76. ^{13}C NMR spectrum of **8** in $CDCl_3$ (100MHz)......37
Figure S77. DEPT-135 spectrum of **8** in $CDCl_3$ (100MHz)......38
Figure S78. ^{1}H NMR spectrum of **9** in $CDCl_3$ (400MHz)......38
Figure S79. ^{13}C NMR spectrum of **9** in $CDCl_3$ (100MHz)......39
Figure S80. DEPT-135 spectrum of **9** in $CDCl_3$ (100MHz)......39
Figure S81. ^{1}H NMR spectrum of **10** in pyridine-d_5 (400MHz)......40
Figure S82. ^{13}C NMR spectrum of **10** in pyridine-d_5 (100MHz)......40
Figure S83. DEPT-135 spectrum of **10** in pyridine-d_5 (100MHz)......41
Figure S84. ^{1}H NMR spectrum of **11** in $CDCl_3$ (400MHz)......41
Figure S85. ^{13}C NMR spectrum of **11** in $CDCl_3$ (100MHz)......42
Figure S86. DEPT-135 spectrum of **11** in $CDCl_3$ (100MHz)......42

Figure S87. ^{1}H NMR spectrum of **12** in $CDCl_3$ (400MHz)... 43
Figure S88. ^{13}C NMR spectrum of **12** in $CDCl_3$ (100MHz)... 43
Figure S89. DEPT-135 spectrum of **12** in $CDCl_3$ (100MHz)... 44

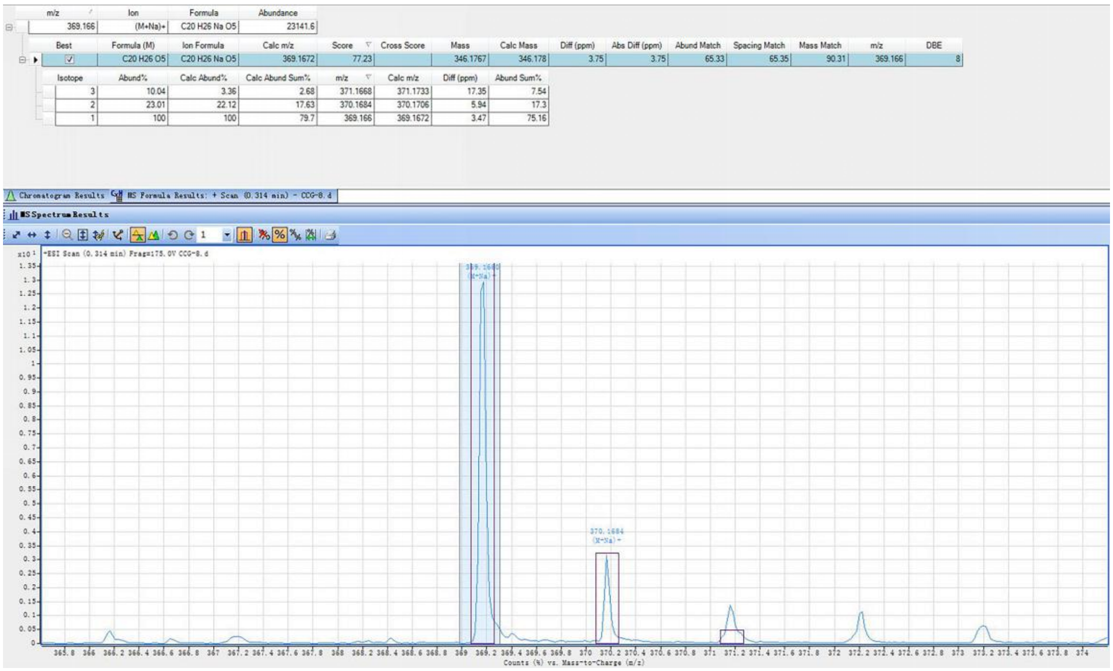

Figure S1. HR-ESI-MS of **1**

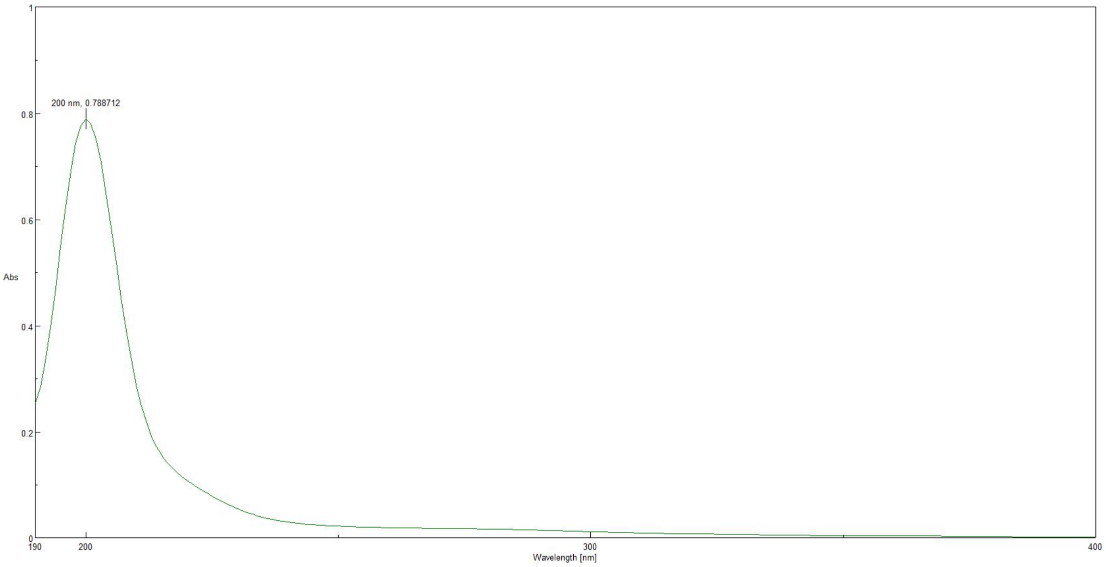

Figure S2. UV spectrum of **1** (CH_3OH)

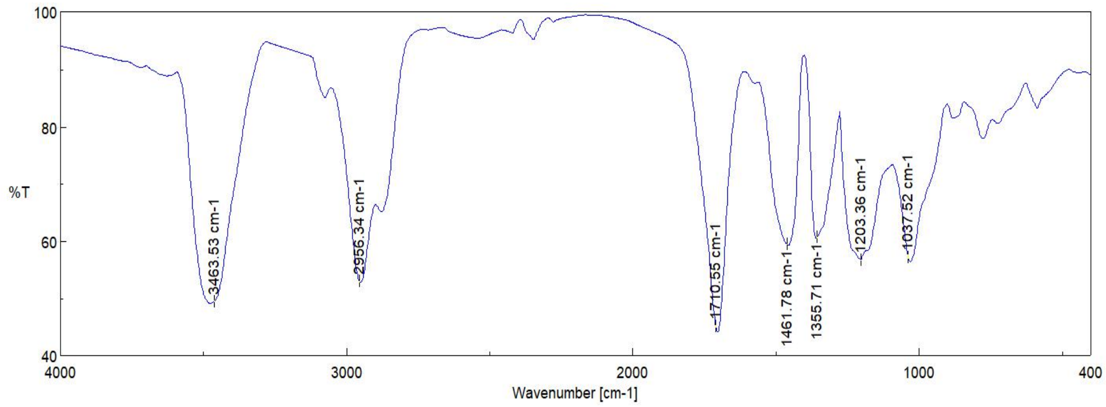

Figure S3. IR spectrum of **1** (KBr disc)

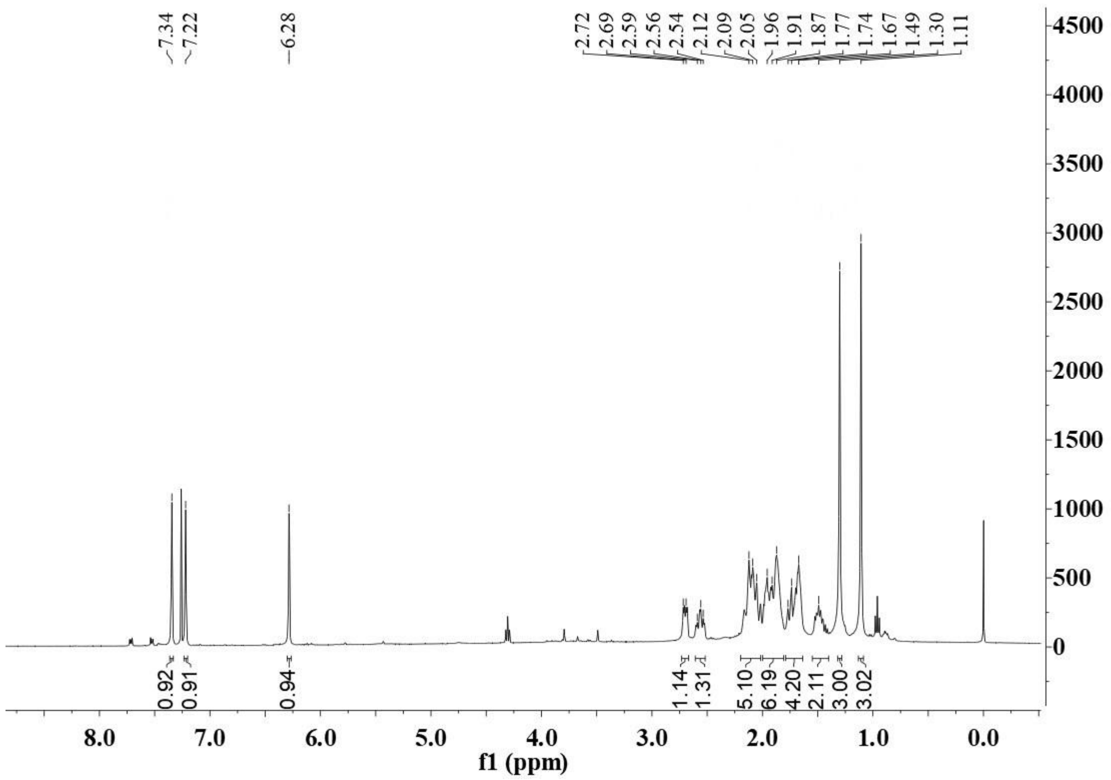

Figure S4. ^{1}H NMR spectrum of **1** in $CDCl_3$ (400 MHz)

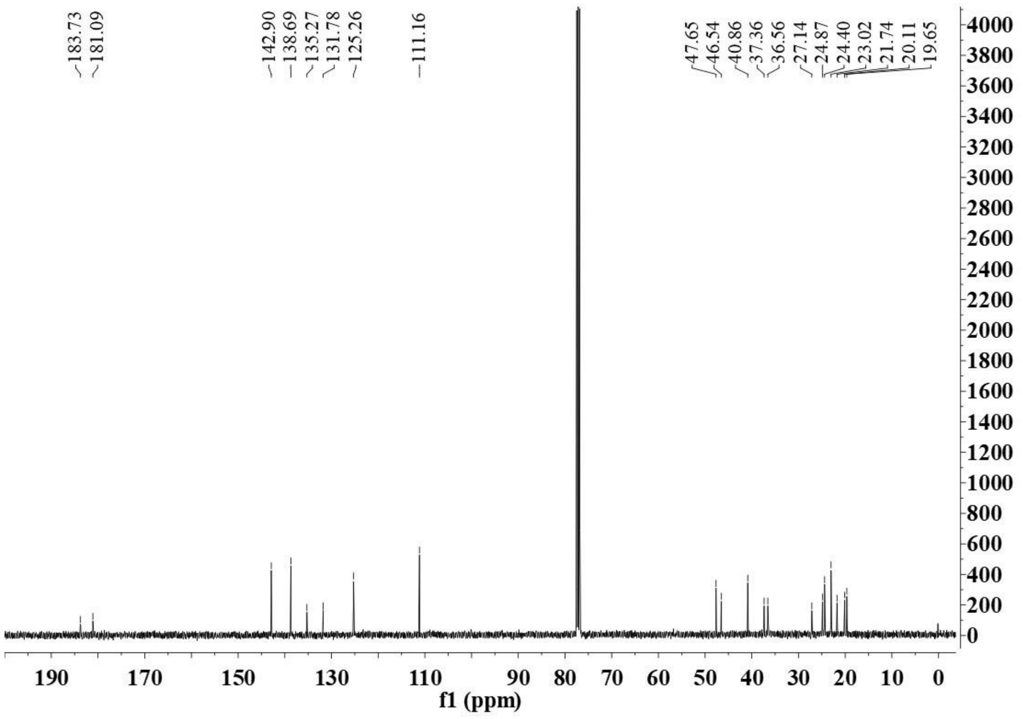

Figure S5. ^{13}C NMR spectrum of **1** in $CDCl_3$ (100 MHz)

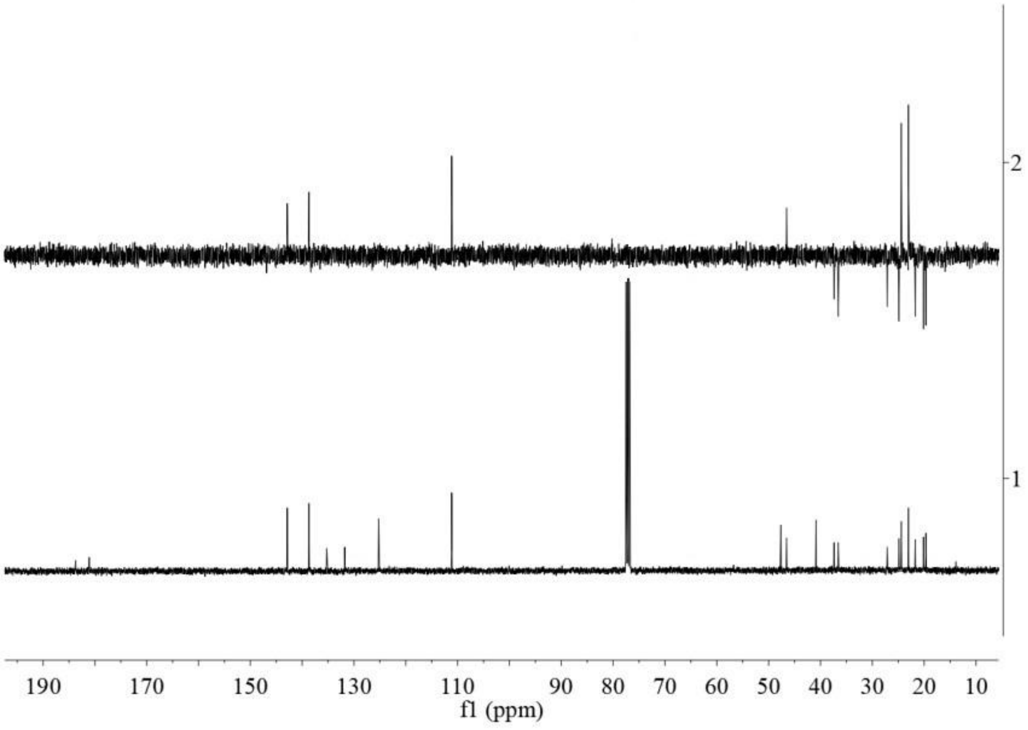

Figure S6. DEPT-135 spectrum of **1** in $CDCl_3$ (100 MHz)

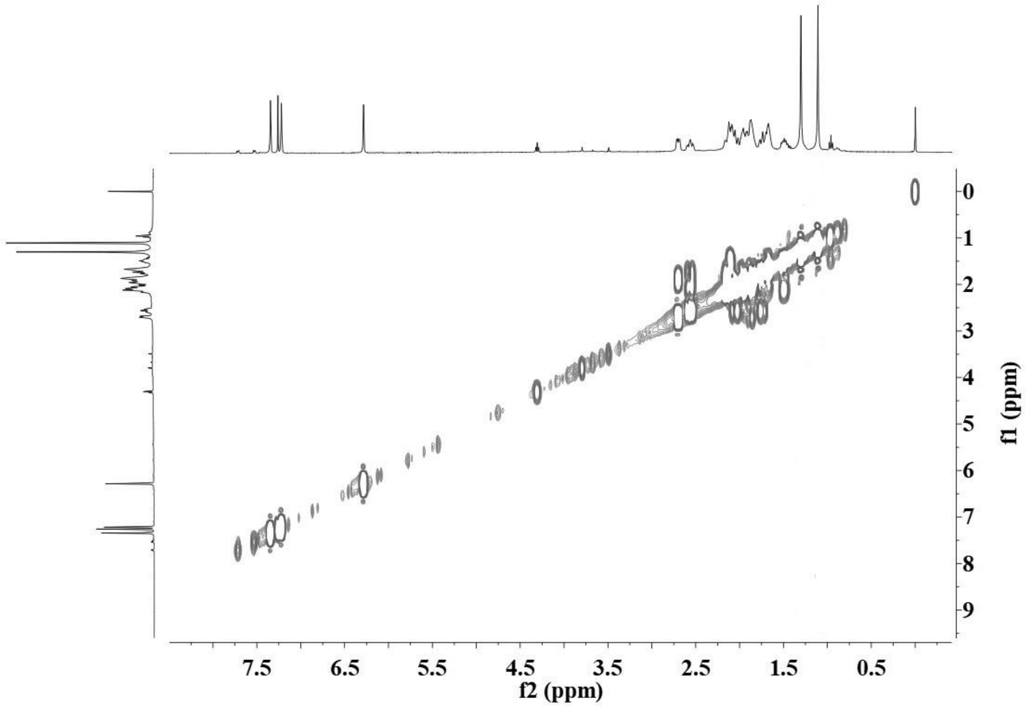

Figure S7. ^{1}H-^{1}H COSY spectrum of **1** in $CDCl_3$

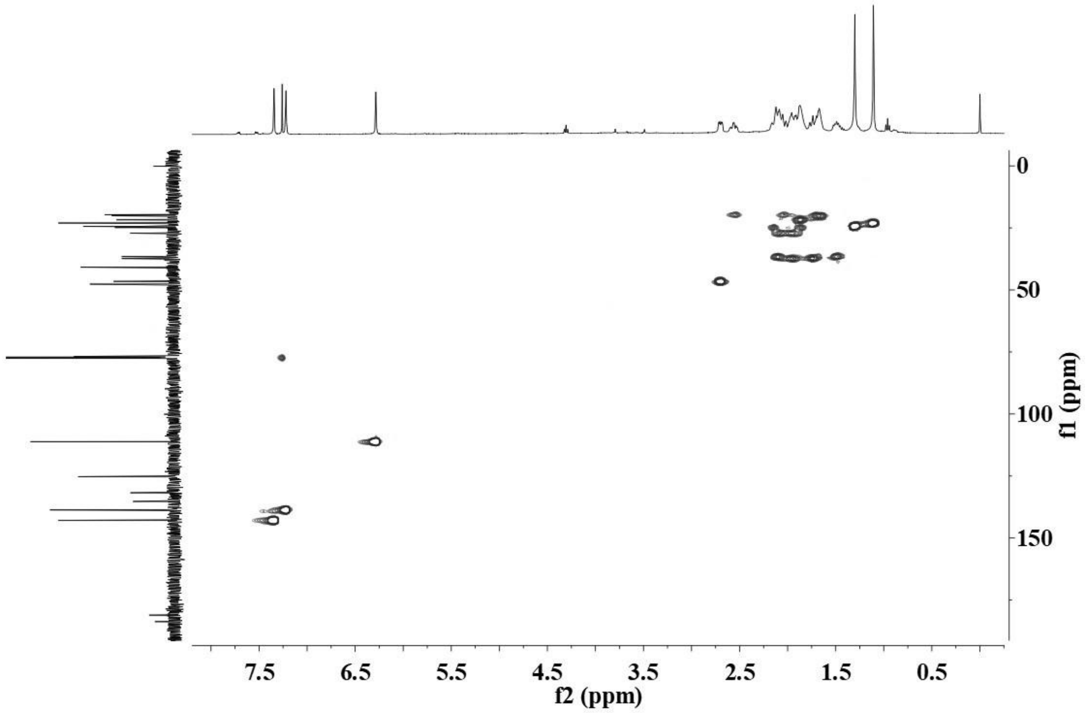

Figure S8. HSQC spectrum of **1** in $CDCl_3$

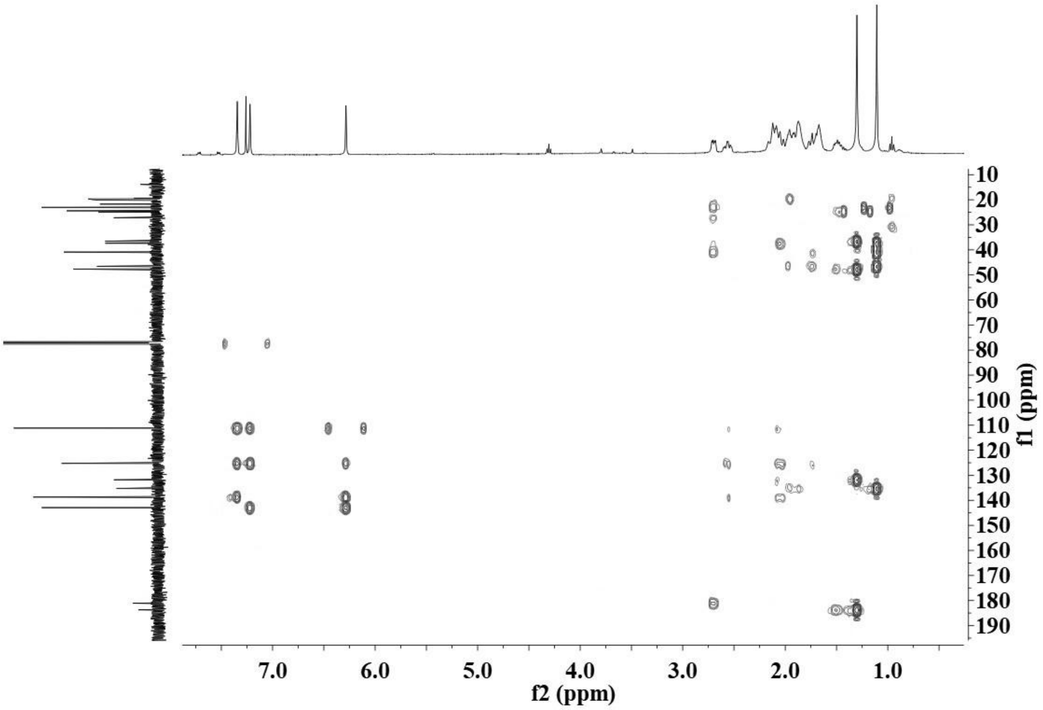

Figure S9. HMBC spectrum of **1** in $CDCl_3$

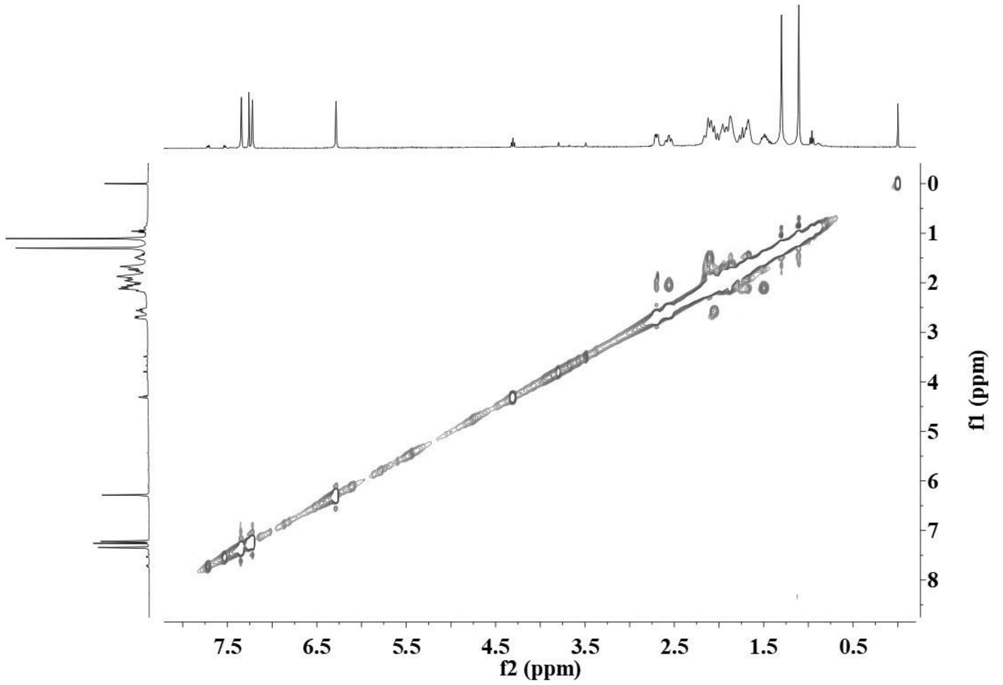

Figure S10. NOESY spectrum of **1** in $CDCl_3$

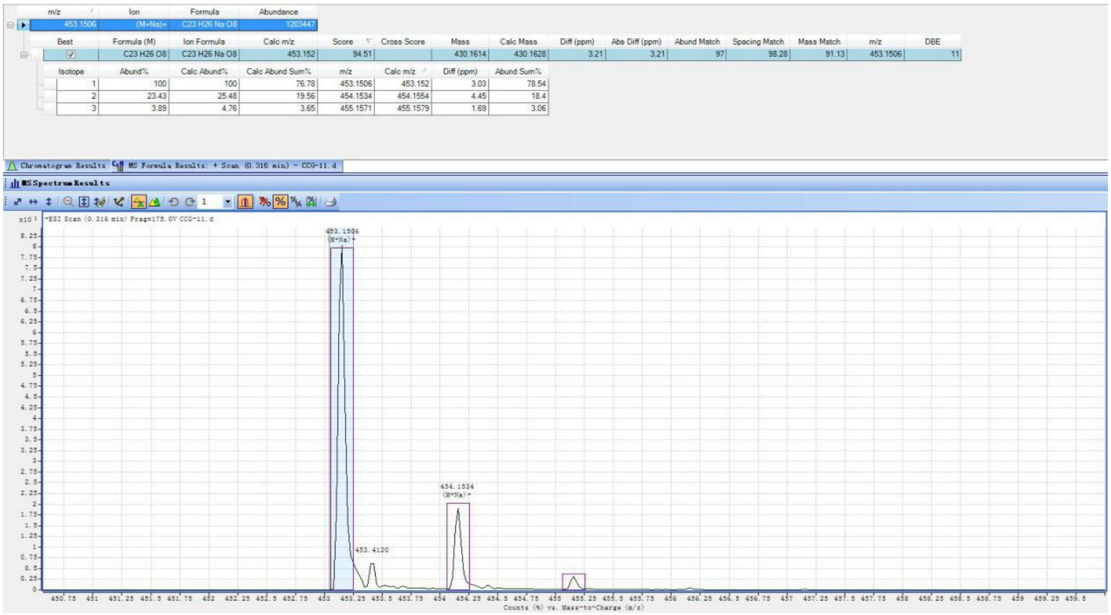

Figure S11. HR-ESI-MS of **2**

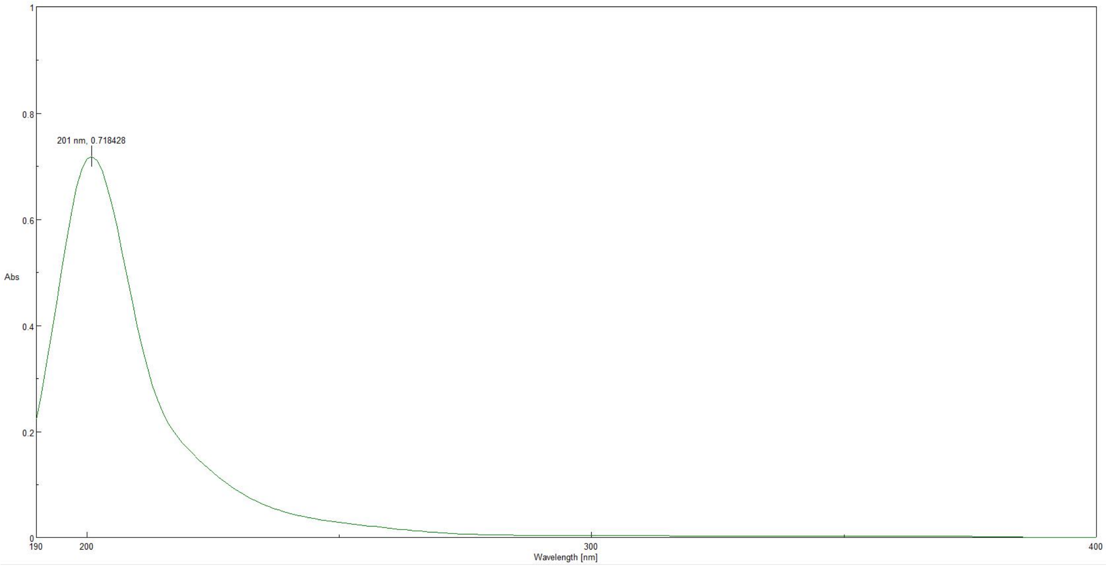

Figure S12. UV spectrum of **2** (CH_3OH)

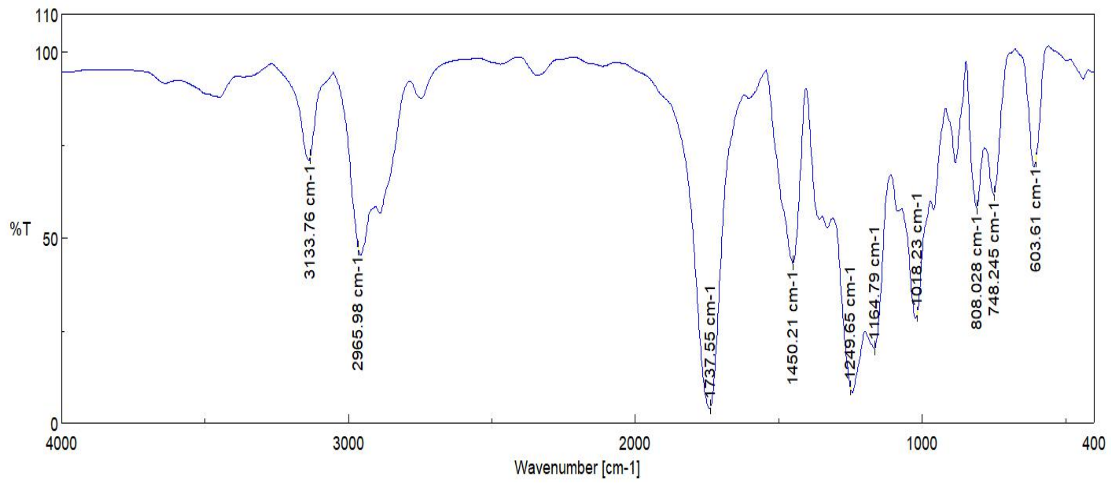

Figure S13. IR spectrum of **2** (KBr disc)

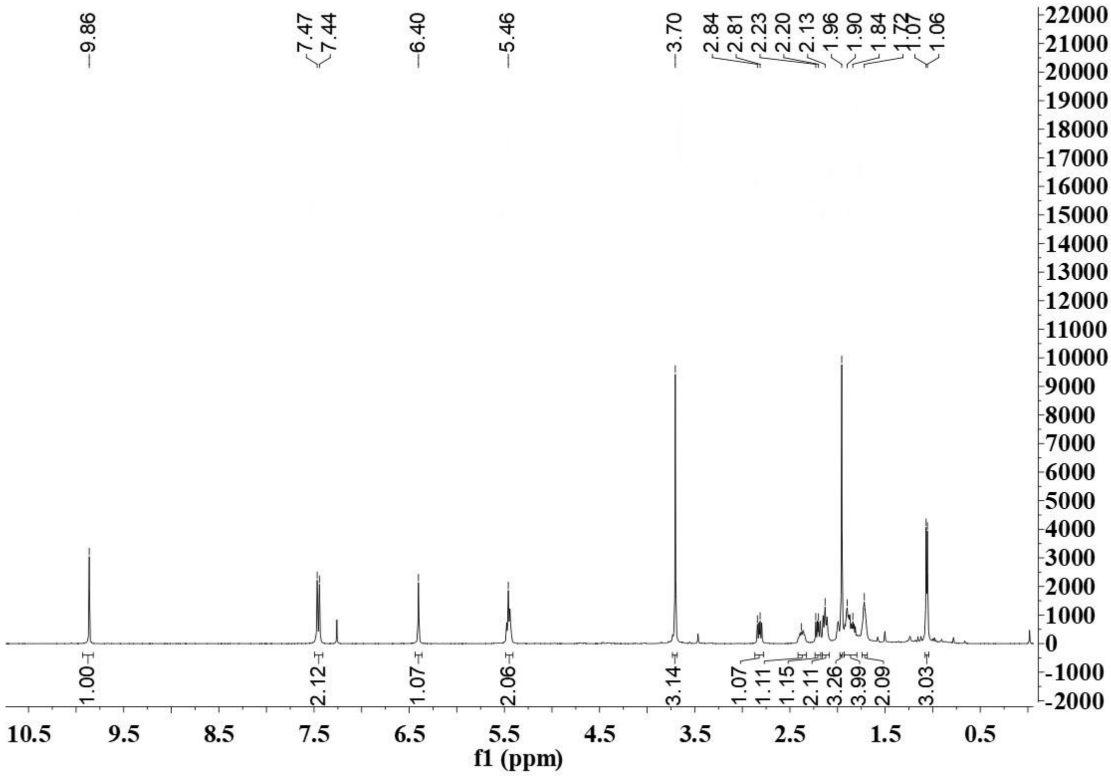

Figure S14. ^{1}H NMR spectrum of **2** in $CDCl_3$ (400 MHz)

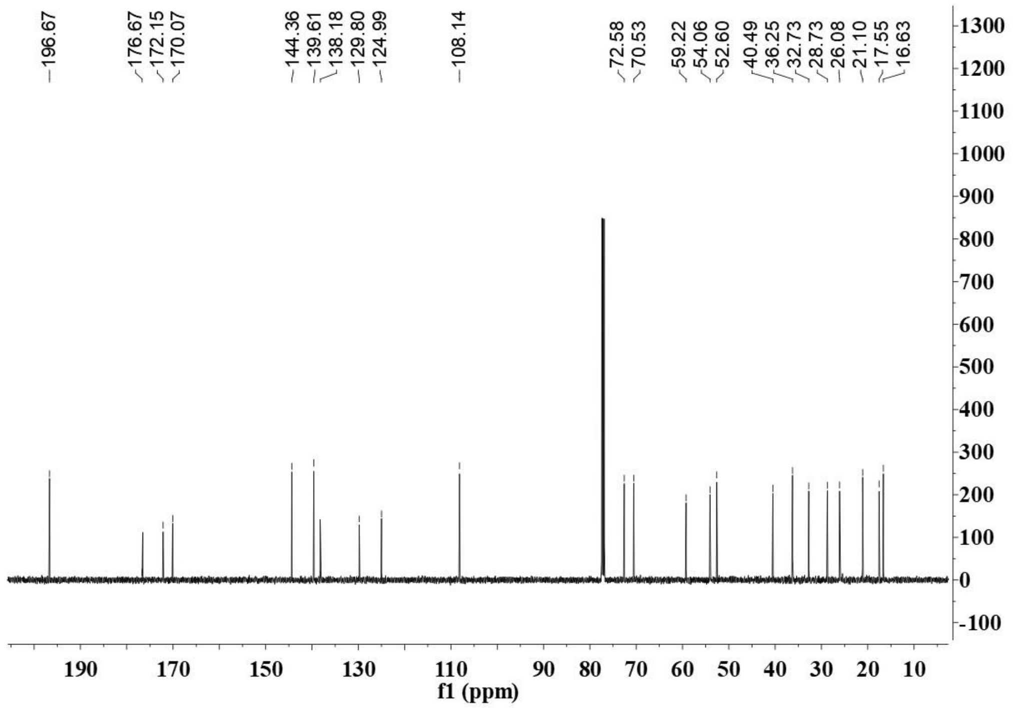

Figure S15. ^{13}C NMR spectrum of **2** in $CDCl_3$ (100 MHz)

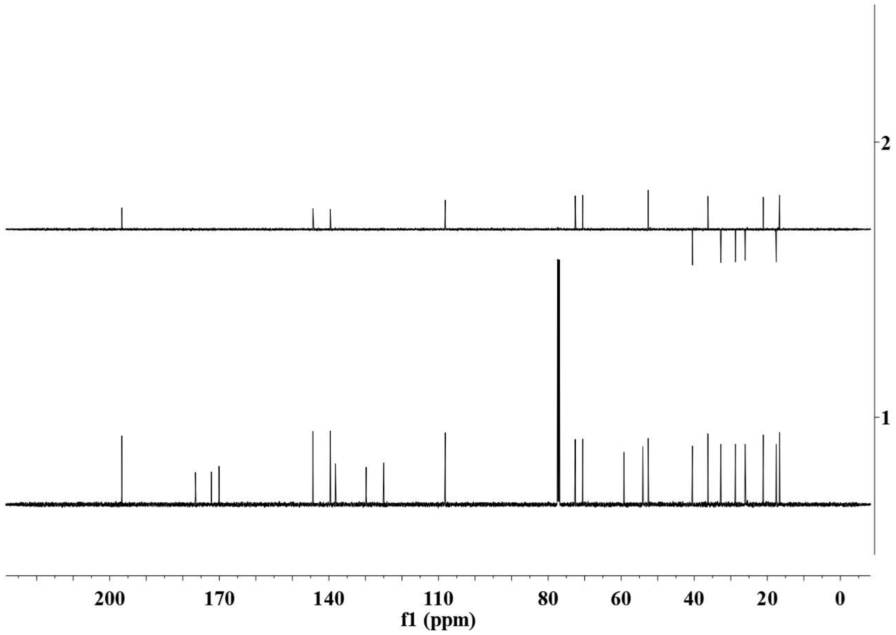

Figure S16. DEPT-135 spectrum of **2** in $CDCl_3$ (100 MHz)

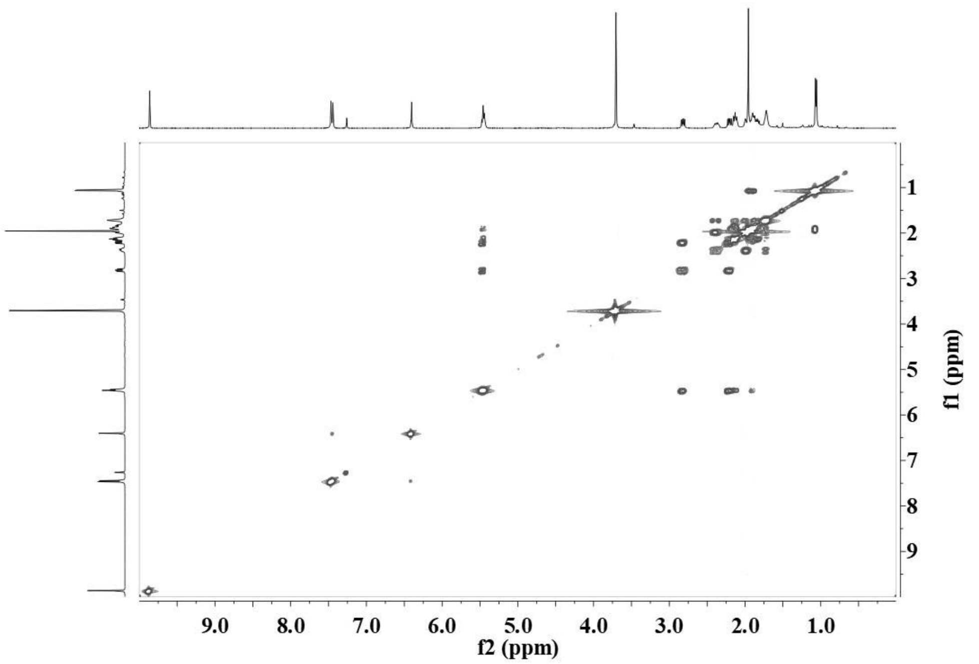

Figure S17. ^{1}H-^{1}H COSY spectrum of **2** in $CDCl_3$

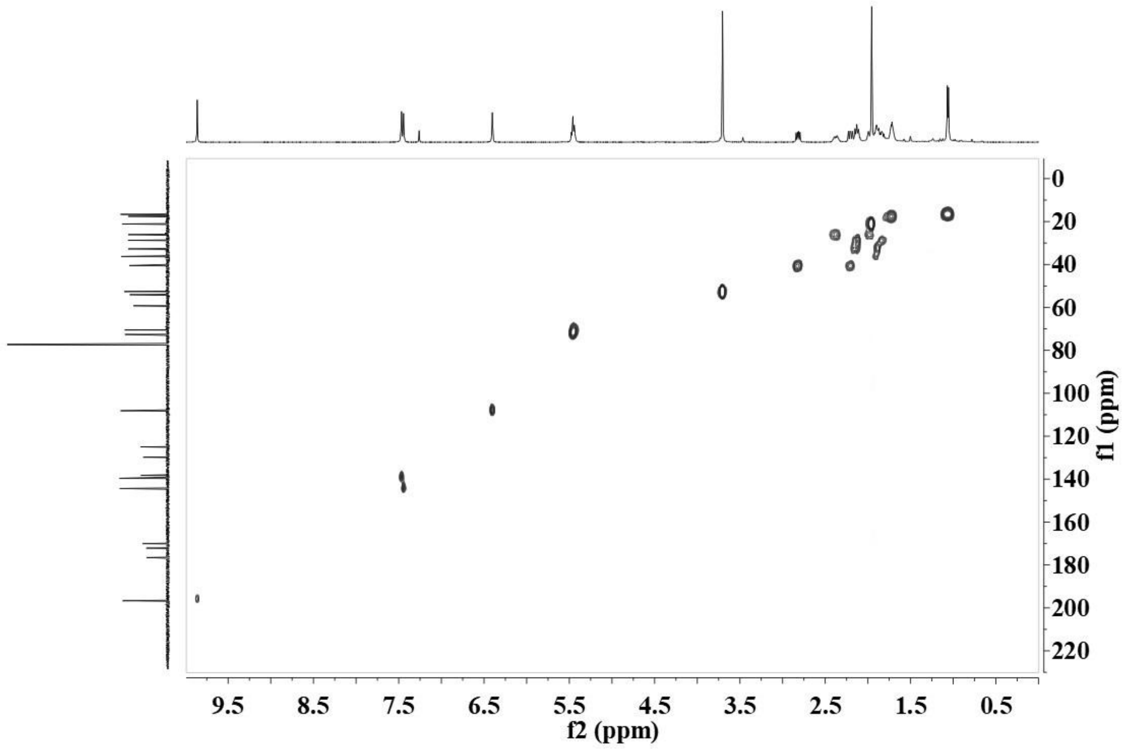

Figure S18. HSQC spectrum of **2** in $CDCl_3$

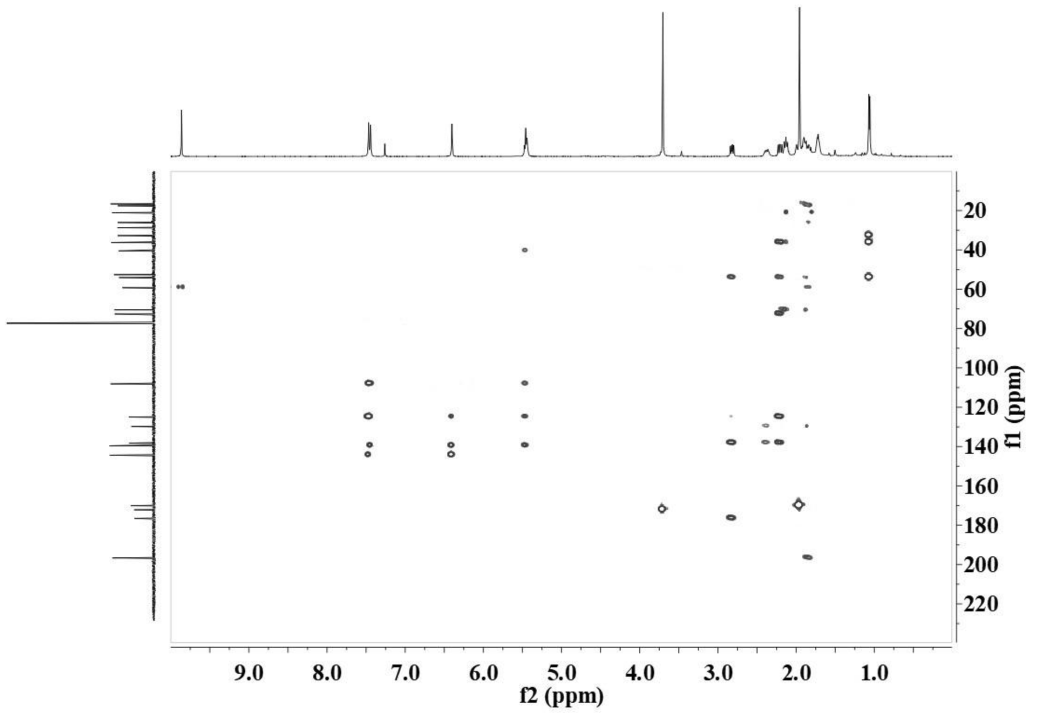

Figure S19. HMBC spectrum of **2** in $CDCl_3$

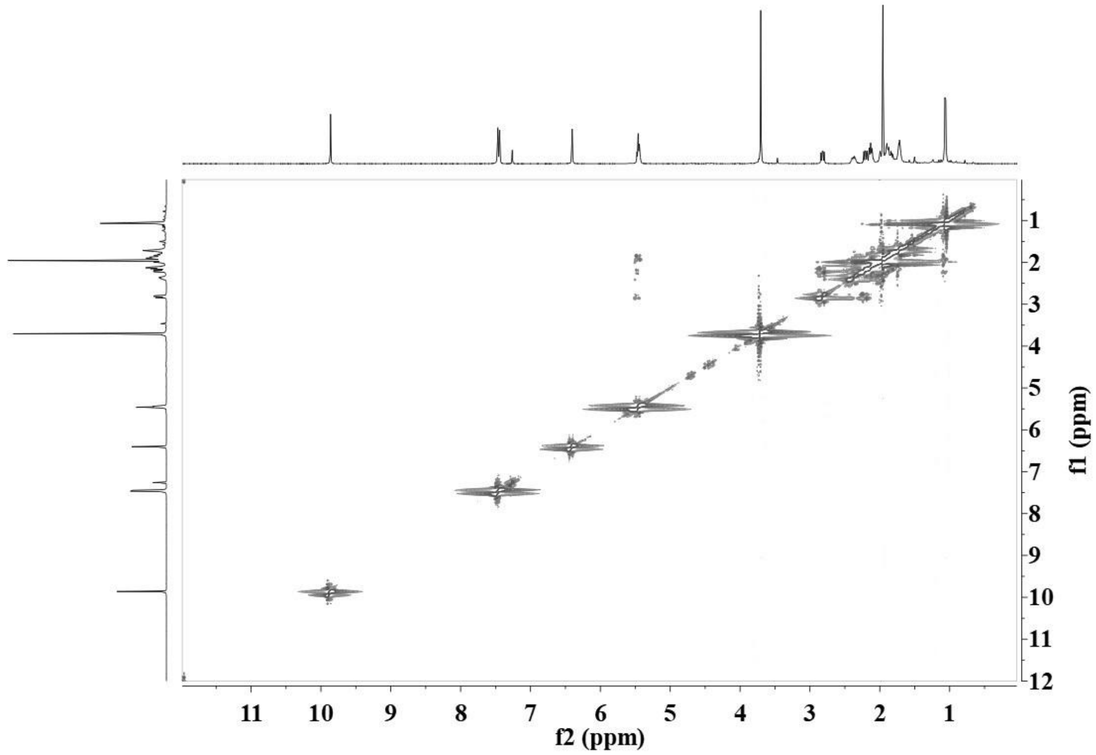

Figure S20. NOESY spectrum of **2** in $CDCl_3$

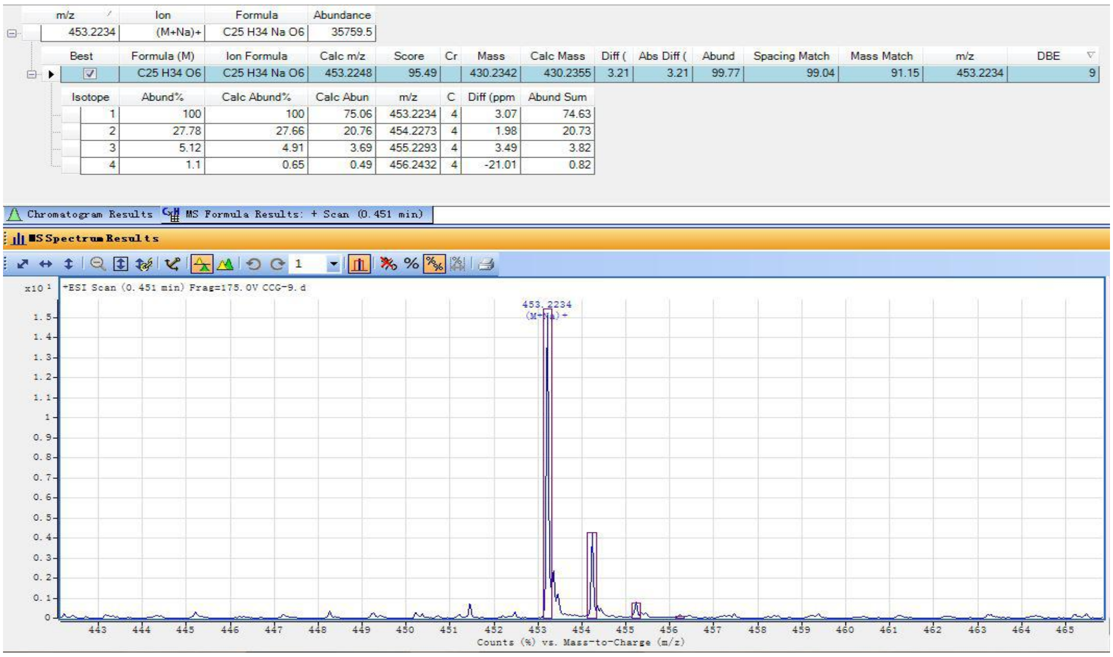

m/z	Ion	Formula	Abundance
453.2234	(M+Na)+	C25 H34 Na O6	35759.5

Best	Formula (M)	Ion Formula	Calc m/z	Score	Cr	Mass	Calc Mass	Diff (	Abs Diff (	Abund	Spacing Match	Mass Match	m/z	DBE
✓	C25 H34 O6	C25 H34 Na O6	453.2248	95.49		430.2342	430.2355	3.21	3.21	99.77	99.04	91.15	453.2234	9

Isotope	Abund%	Calc Abund%	Calc Abun	m/z	C	Diff (ppm	Abund Sum
1	100	100	75.06	453.2234	4	3.07	74.63
2	27.78	27.66	20.76	454.2273	4	1.98	20.73
3	5.12	4.91	3.69	455.2293	4	3.49	3.82
4	1.1	0.65	0.49	456.2432	4	-21.01	0.82

Figure S21. HR-ESI-MS of **3**

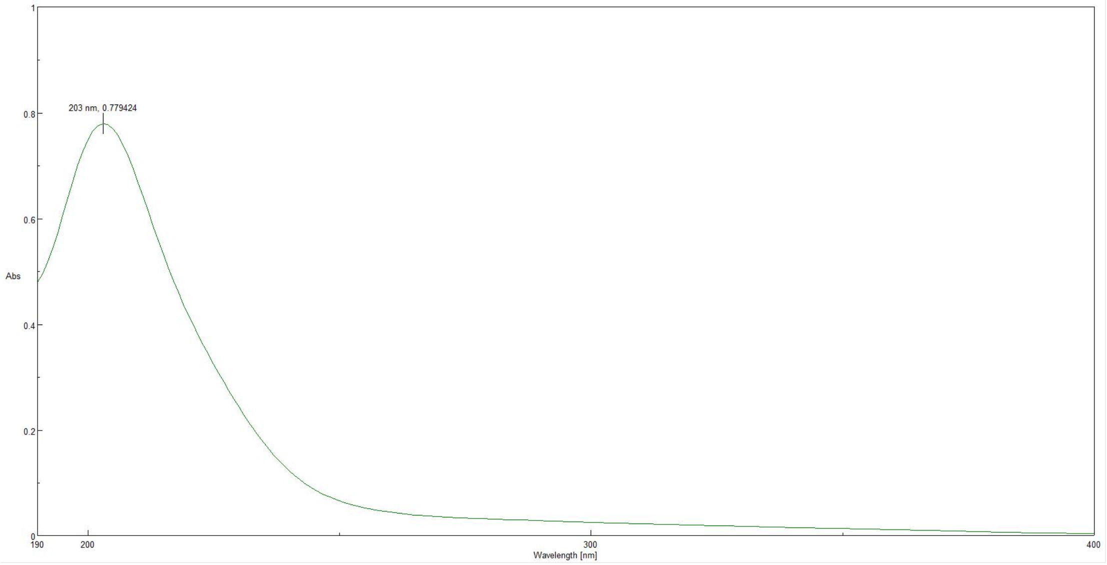

Figure S22. UV spectrum of **3** (CH_3OH)

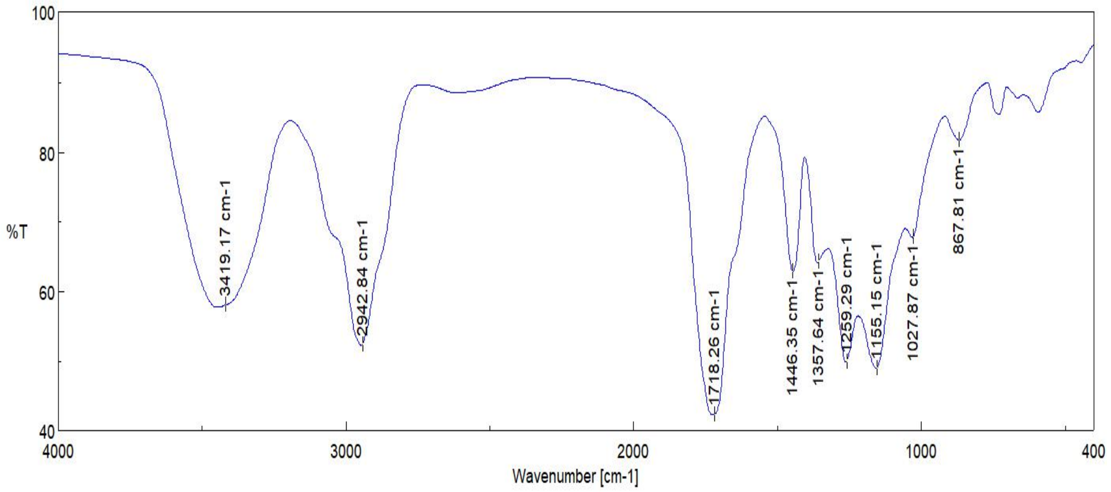

Figure S23. IR spectrum of **3** (KBr disc)

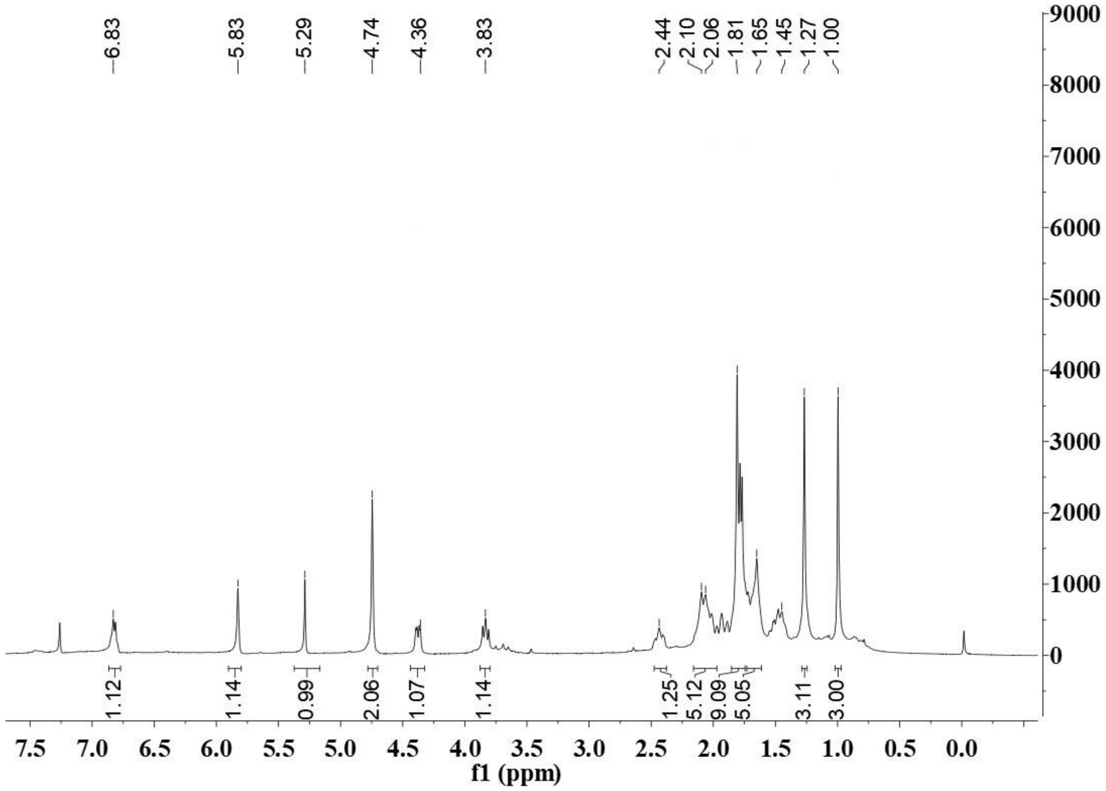

Figure S24. ^{1}H NMR spectrum of **3** in $CDCl_3$ (400 MHz)

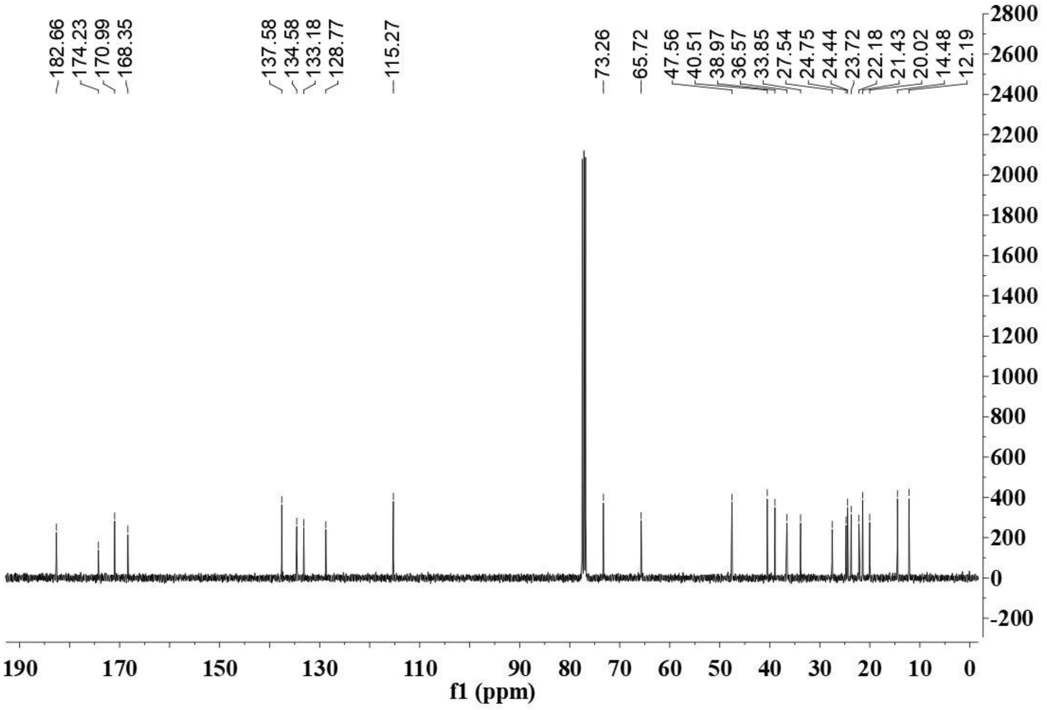

Figure S25. ^{13}C NMR spectrum of **3** in $CDCl_3$ (100 MHz)

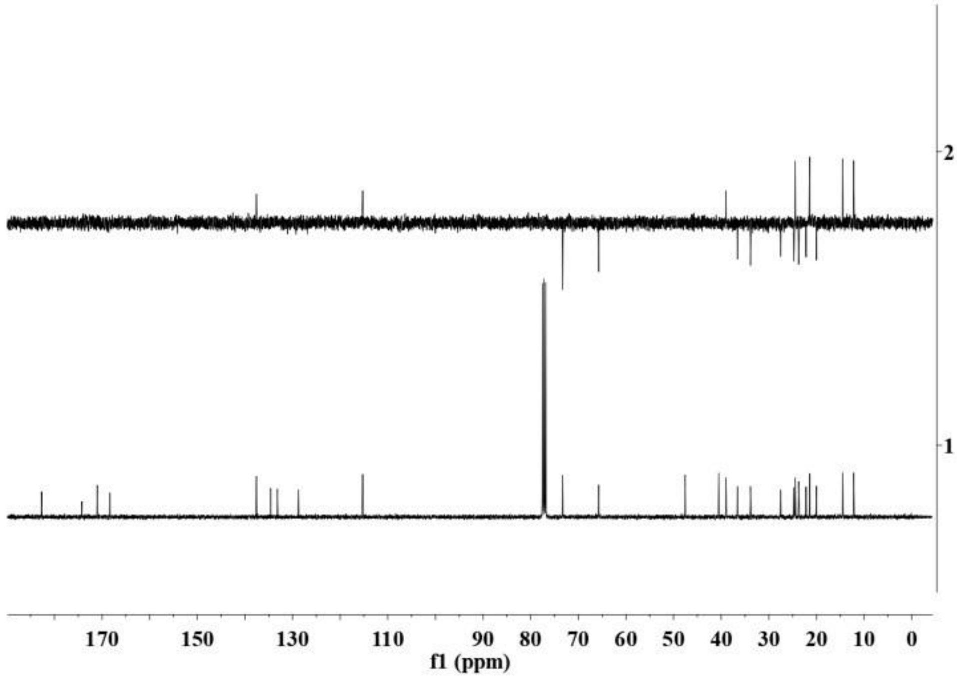

Figure S26. DEPT-135 spectrum of **3** in $CDCl_3$ (100 MHz)

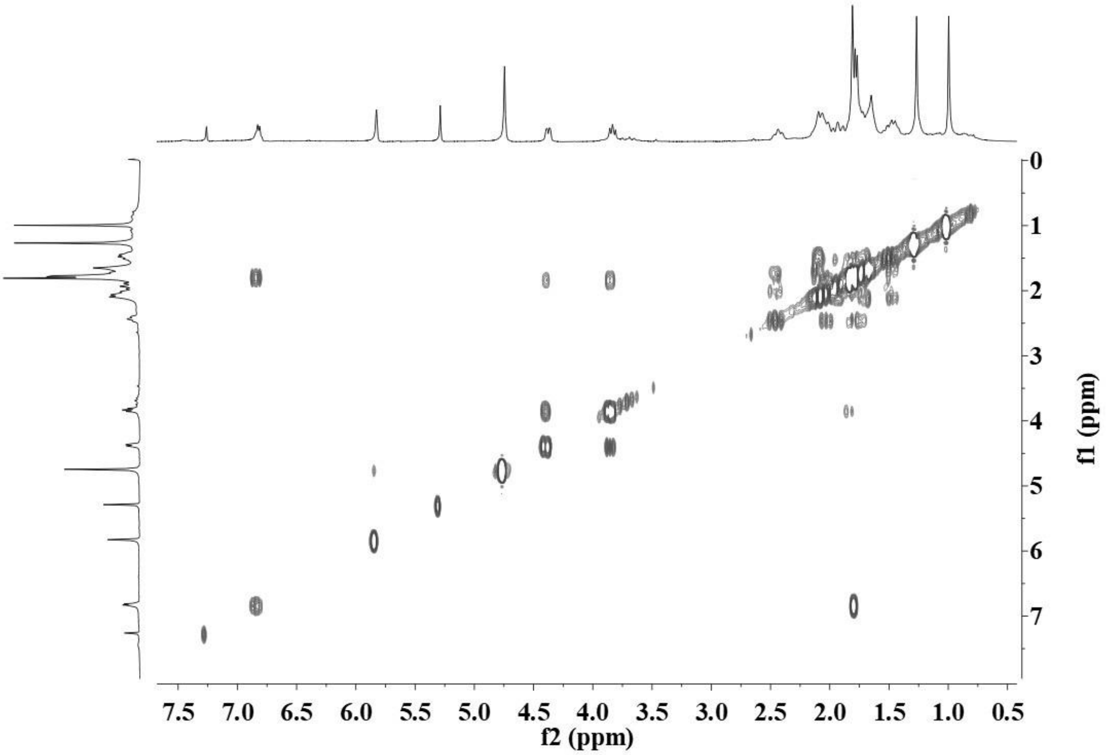

Figure S27. ^{1}H-^{1}H COSY spectrum of **3** in $CDCl_3$

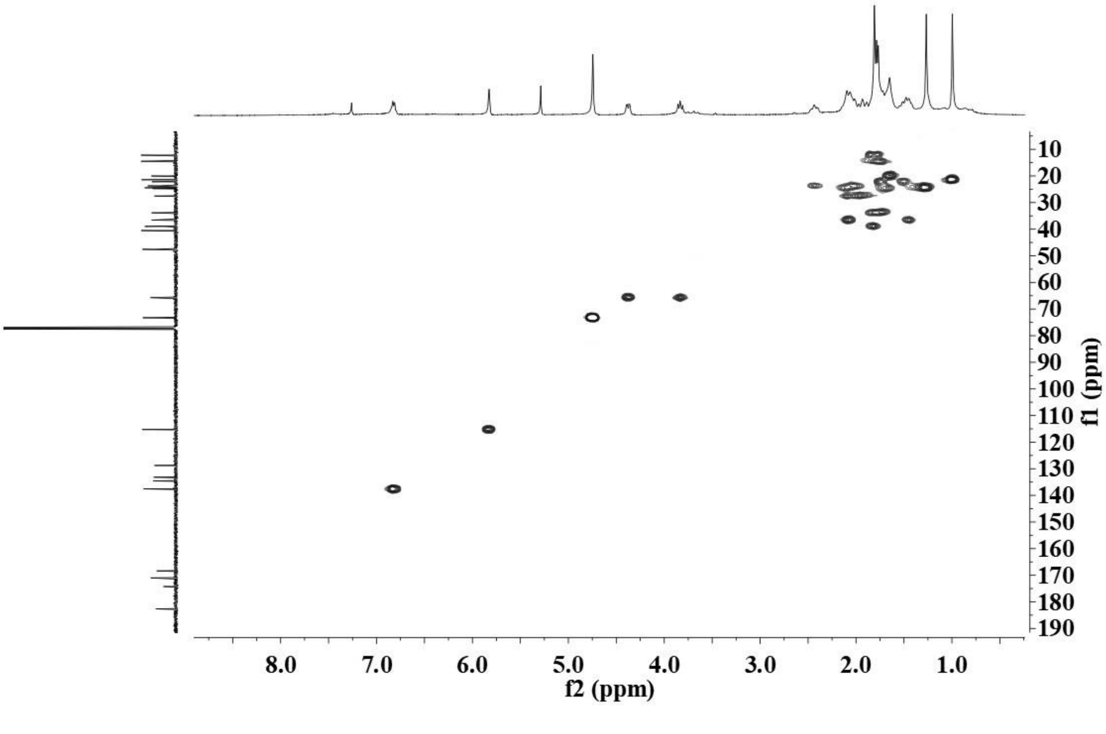

Figure S28. HSQC spectrum of **3** in $CDCl_3$

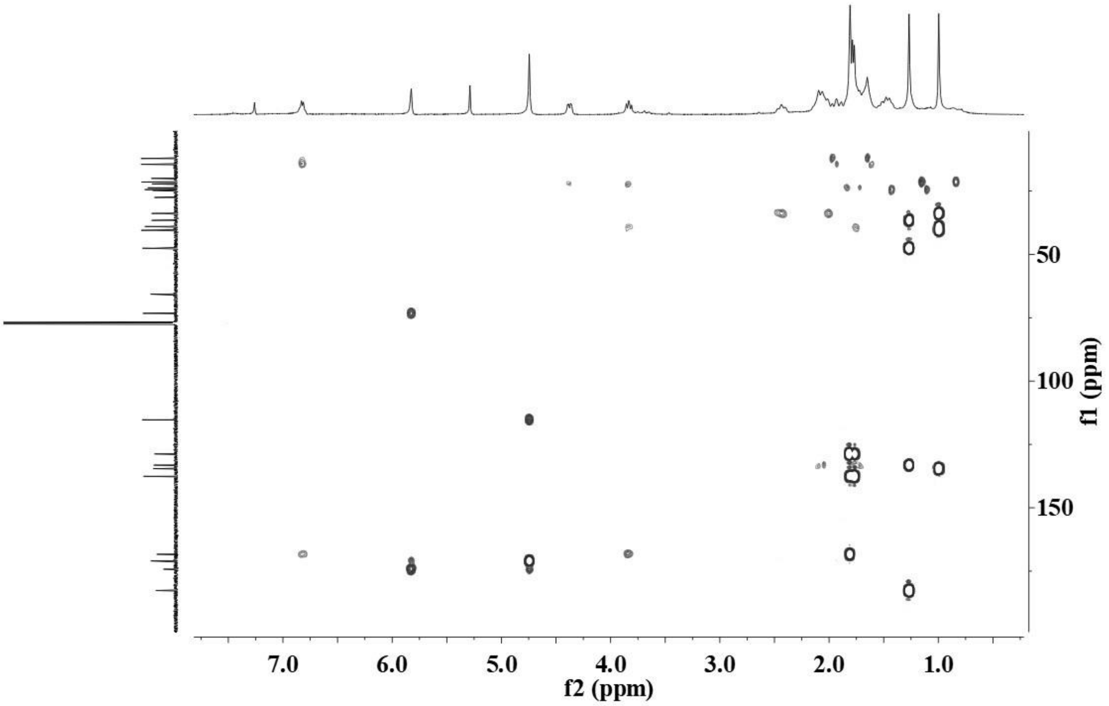

Figure S29. HMBC spectrum of **3** in $CDCl_3$

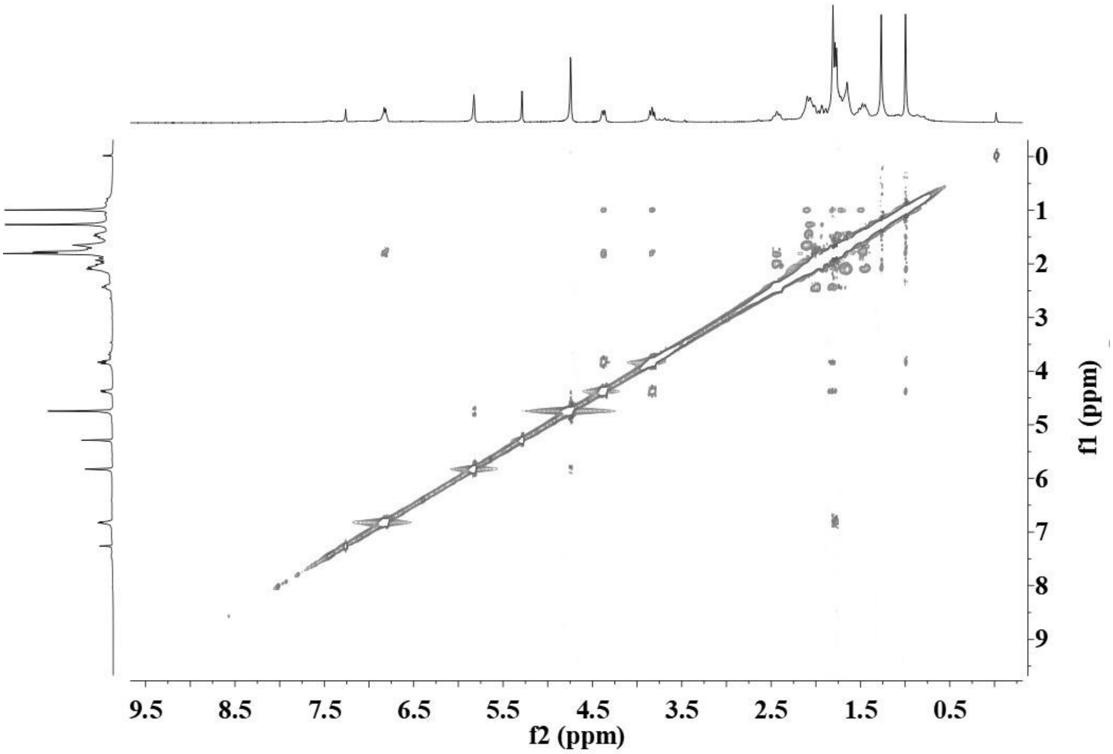

Figure S30. NOESY spectrum of **3** in $CDCl_3$

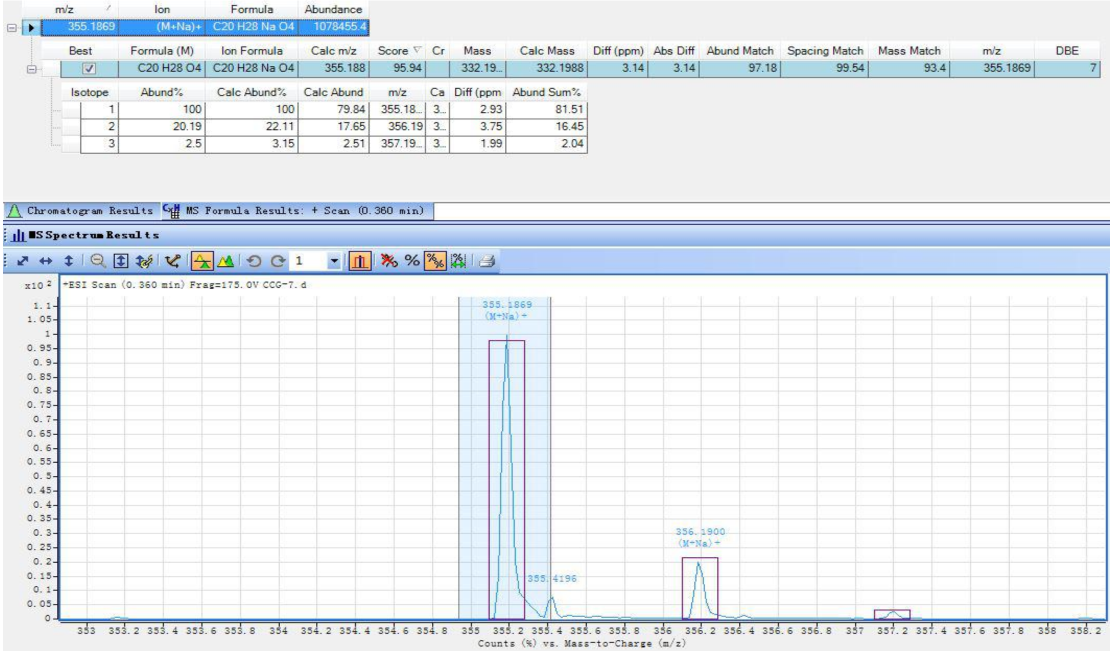

Figure S31. HR-ESI-MS of **4**

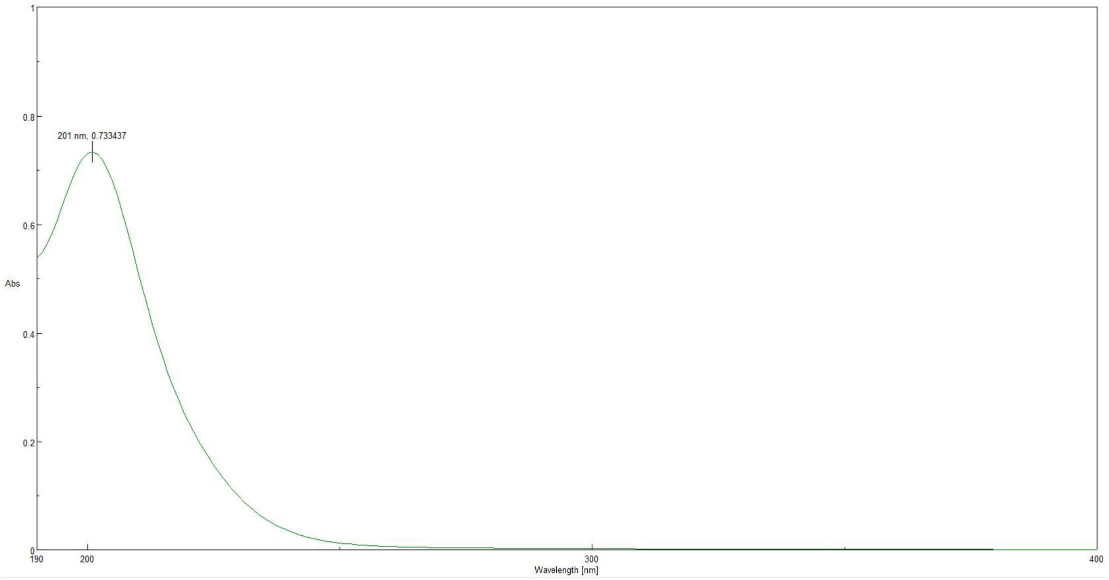

Figure S32. UV spectrum of **4** (CH_3OH)

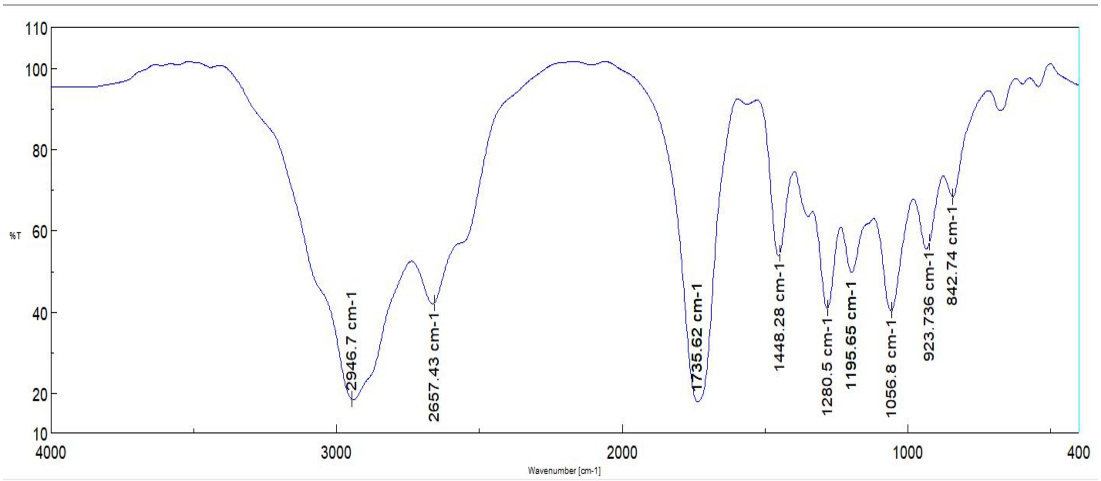

Figure S33. IR spectrum of **4** (KBr disc)

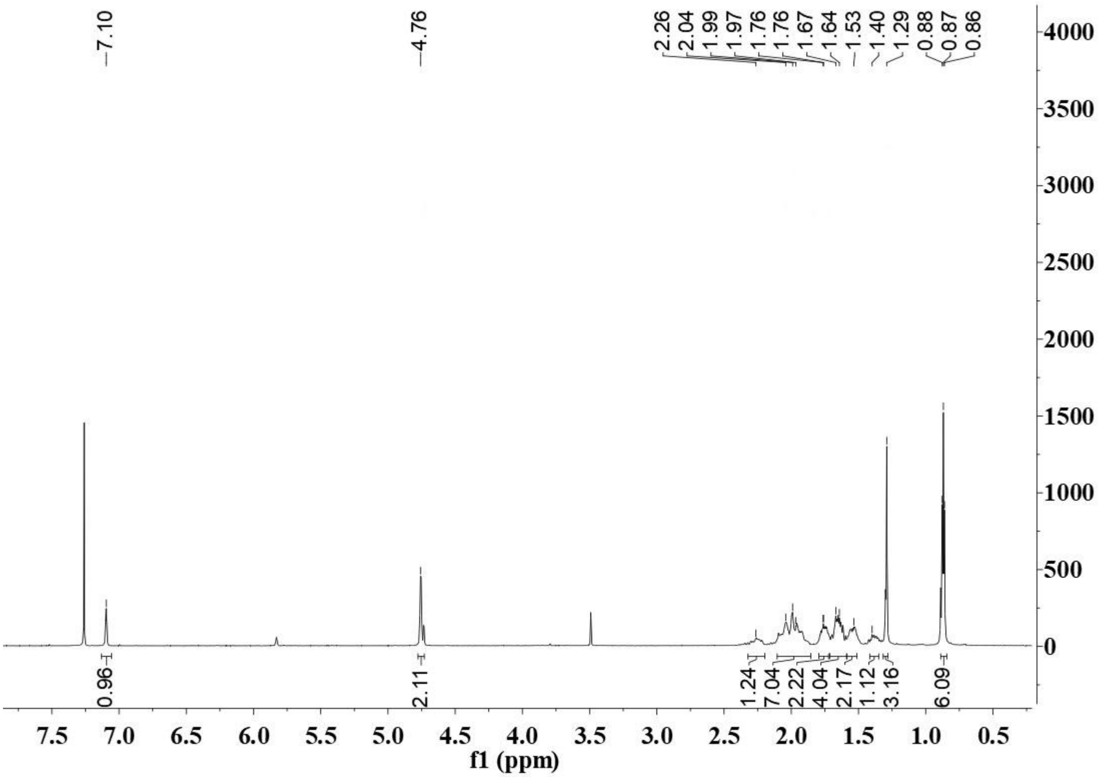

Figure S34. ^{1}H NMR spectrum of **4** in $CDCl_3$ (400 MHz)

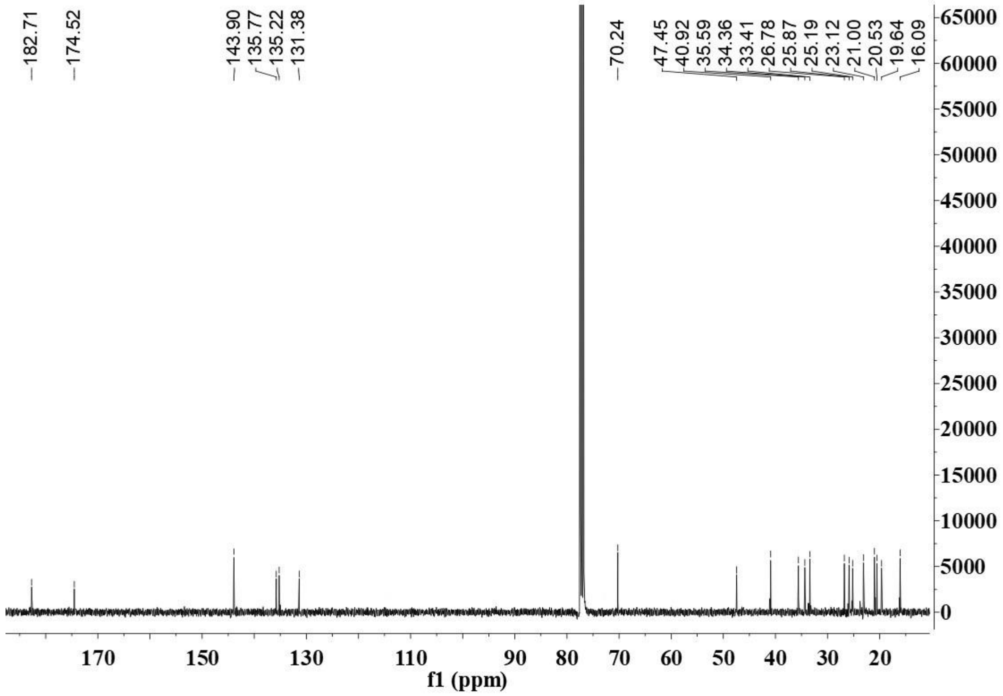

Figure S35. ^{13}C NMR spectrum of **4** in $CDCl_3$ (100 MHz)

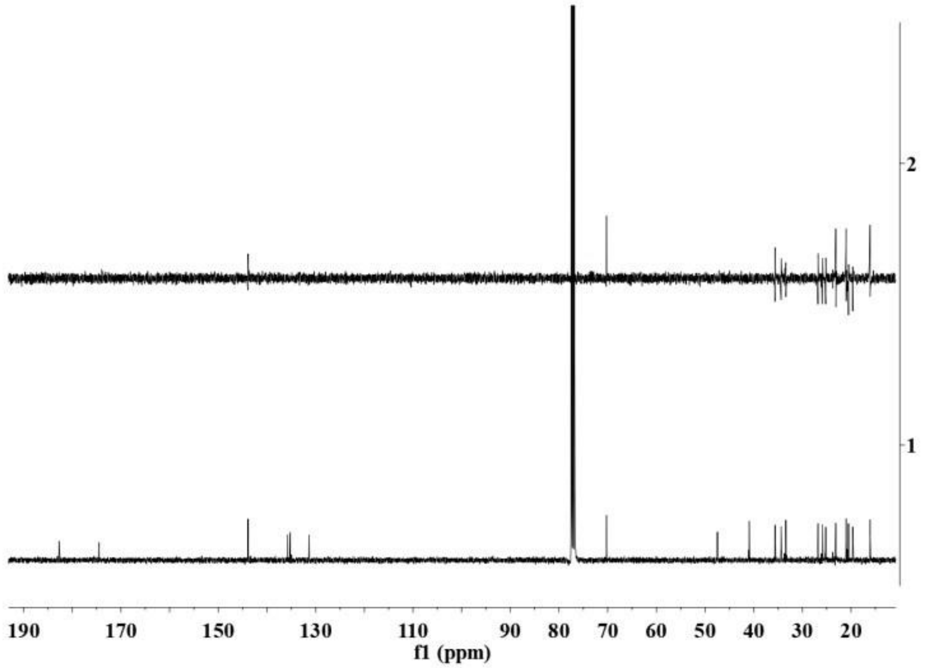

Figure S36. DEPT-135 spectrum of **4** in $CDCl_3$ (100MHz)

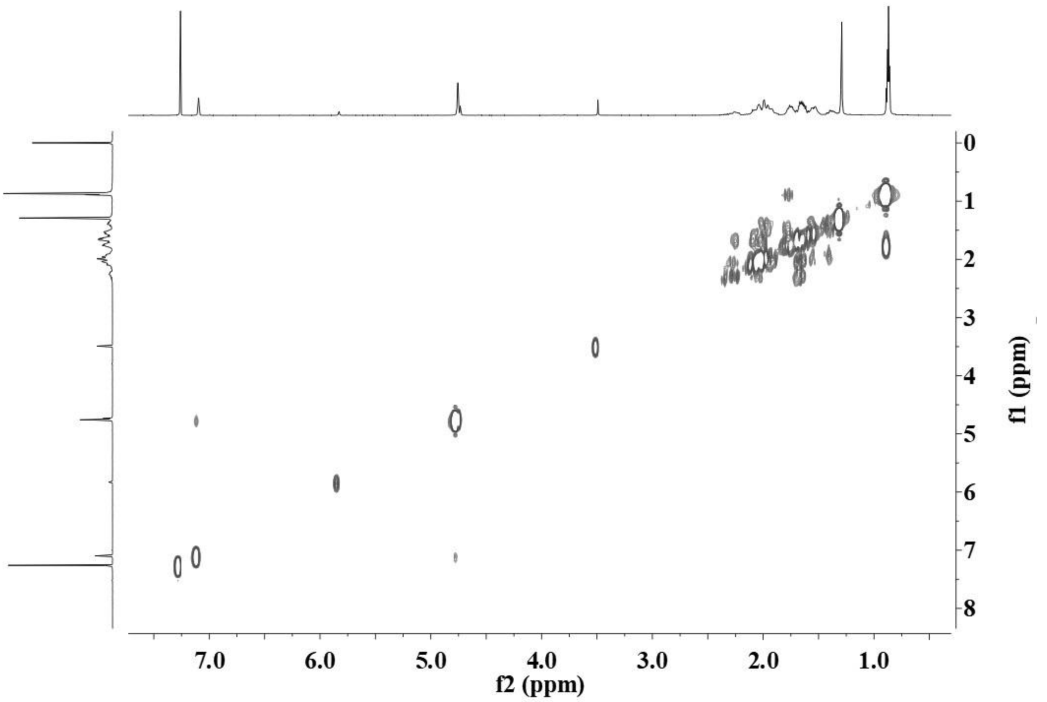

Figure S37. ^{1}H-^{1}H COSY spectrum of **4** in $CDCl_3$

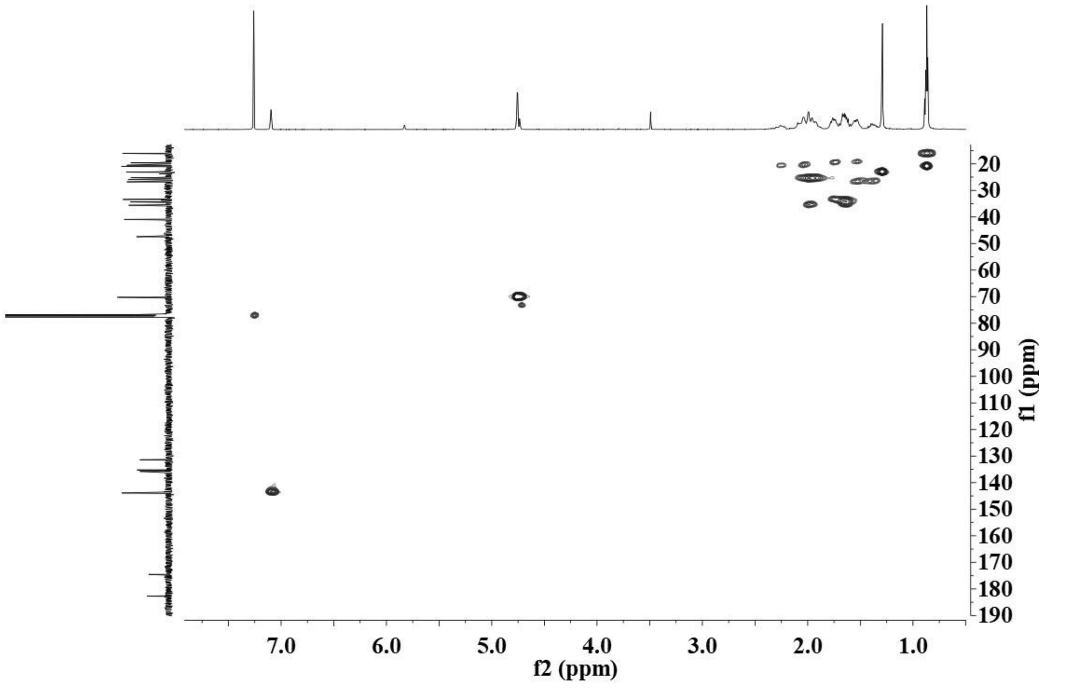

Figure S38. HSQC spectrum of **4** in $CDCl_3$

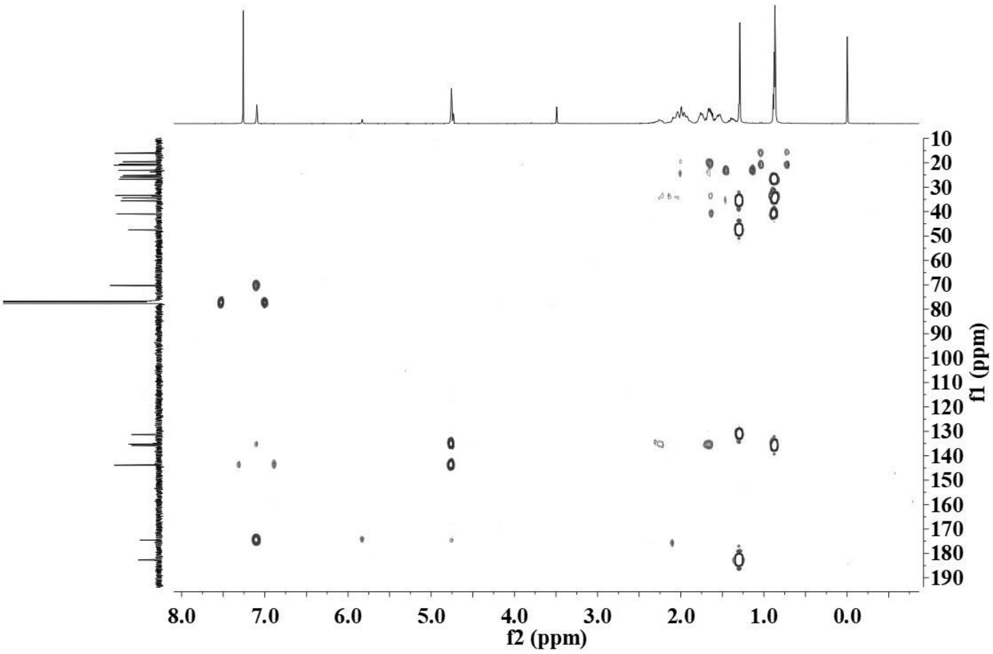

Figure S39. HMBC spectrum of **4** in $CDCl_3$

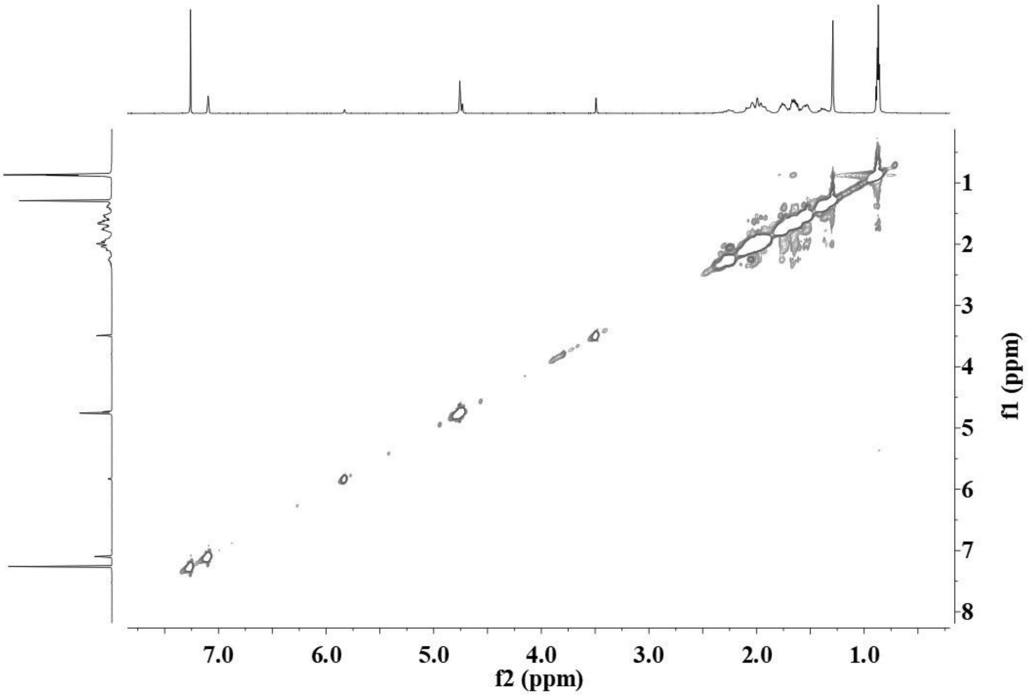

Figure S40. NOESY spectrum of **4** in $CDCl_3$

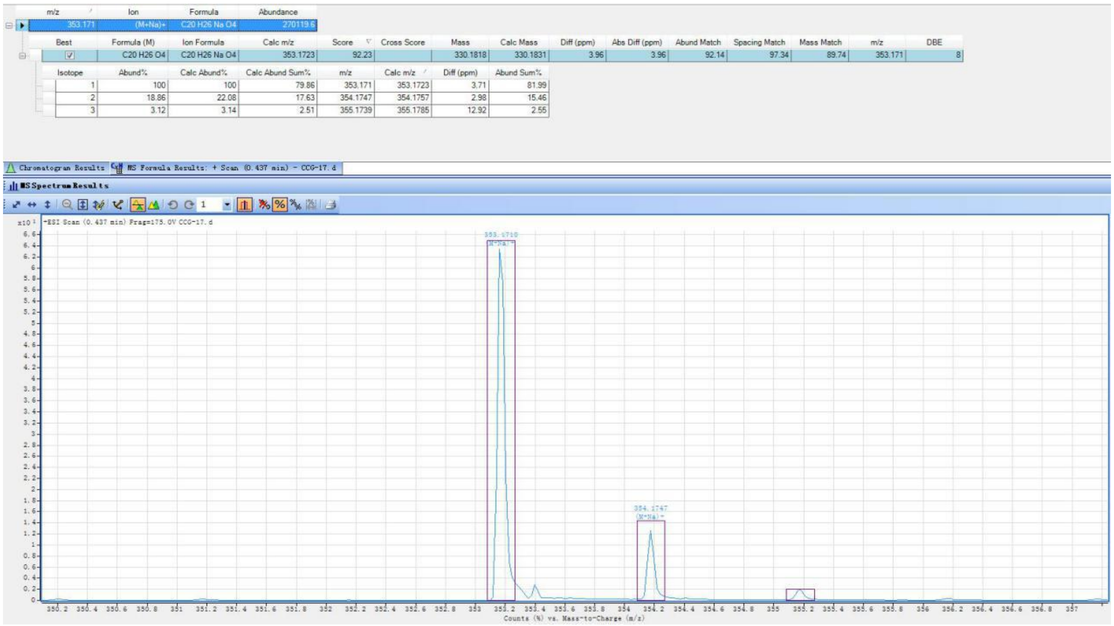

Figure S41. HR-ESI-MS of **5**

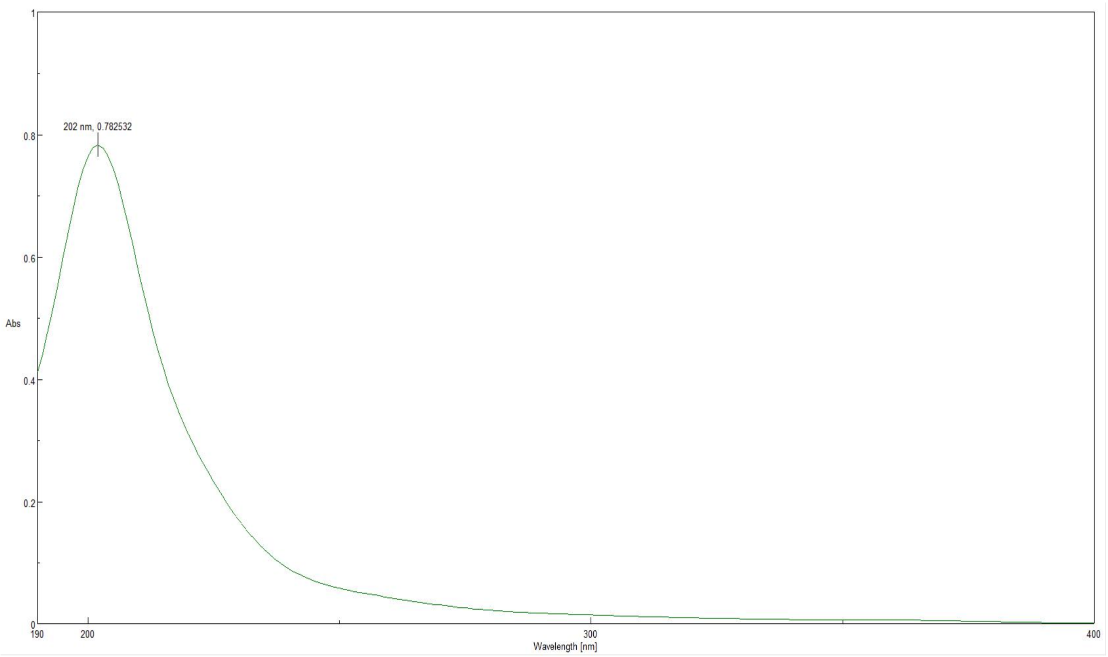

Figure S42. UV spectrum of **5** (CH_3OH)

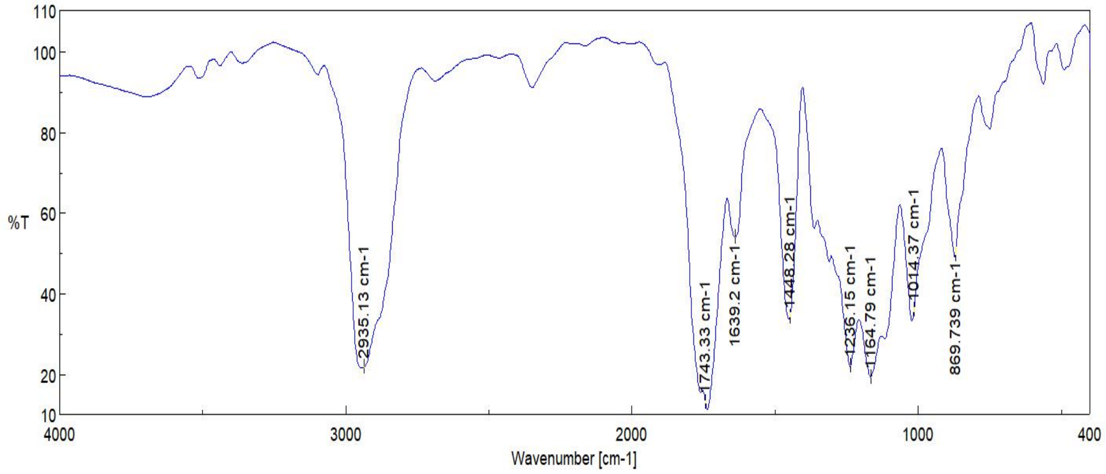

Figure S43. IR spectrum of **5** (KBr disc)

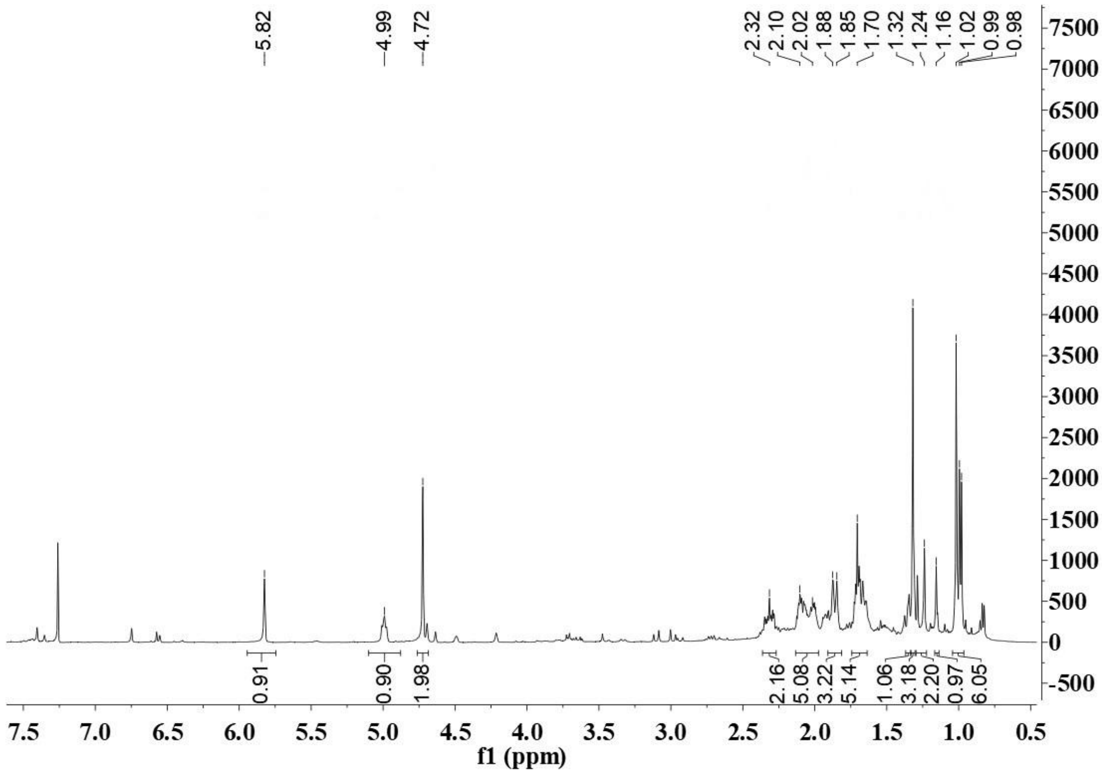

Figure S44. ^{1}H NMR spectrum of **5** in $CDCl_3$ (400 MHz)

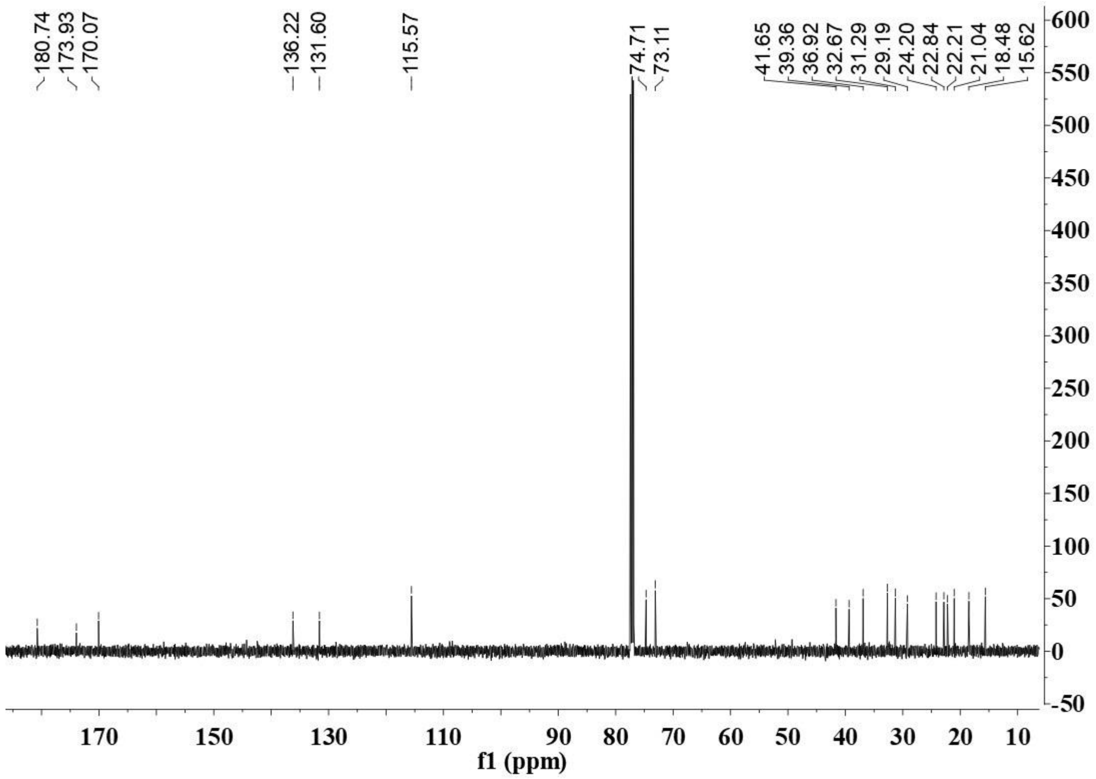

Figure S45. ^{13}C NMR spectrum of **5** in $CDCl_3$ (100 MHz)

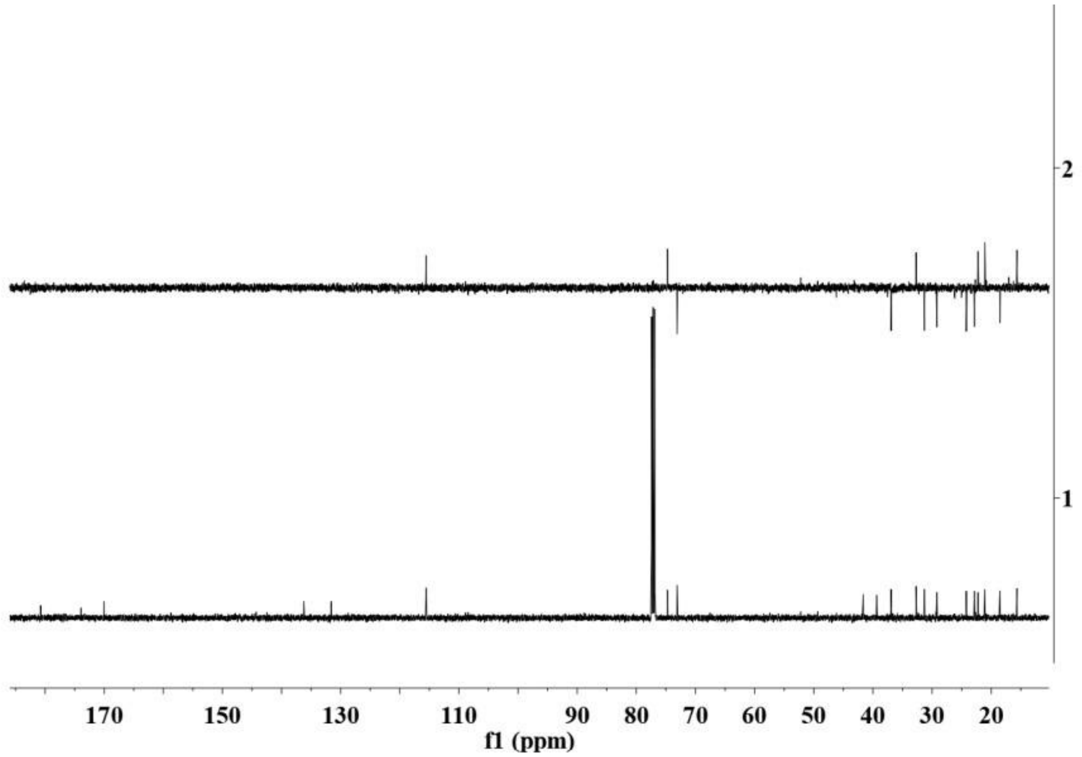

Figure S46. DEPT-135 spectrum of **5** in $CDCl_3$ (100 MHz)

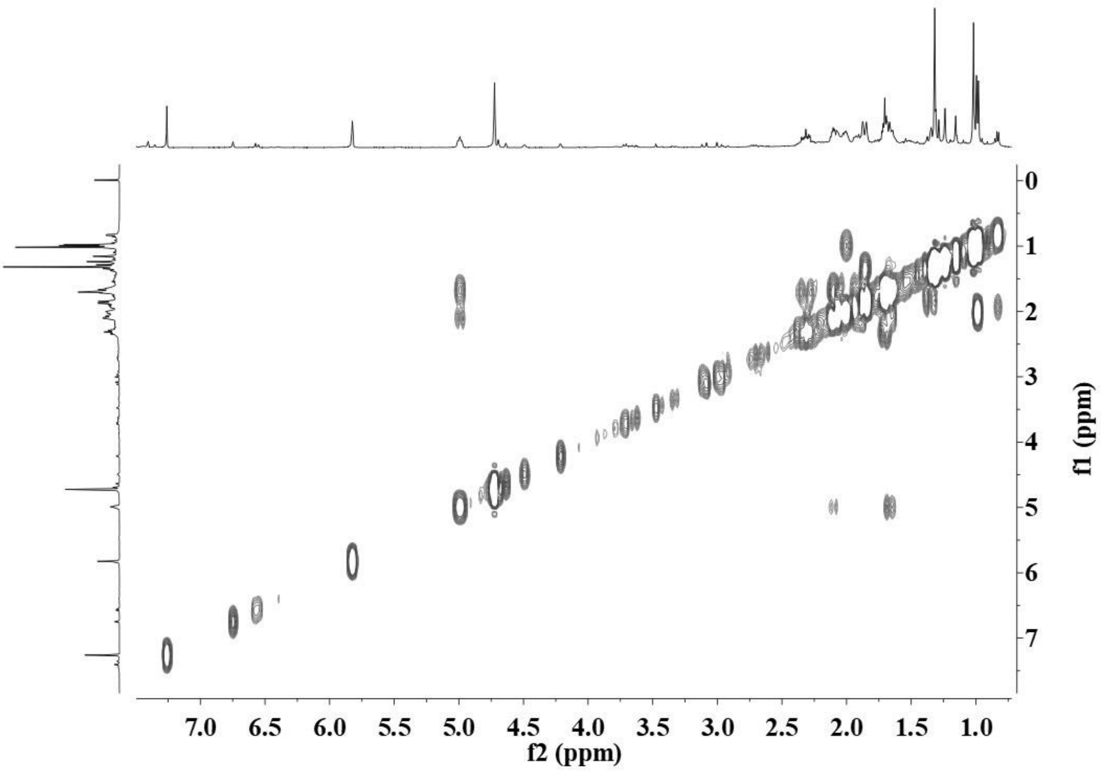

Figure S47. ^{1}H-^{1}H COSY spectrum of **5** in $CDCl_3$

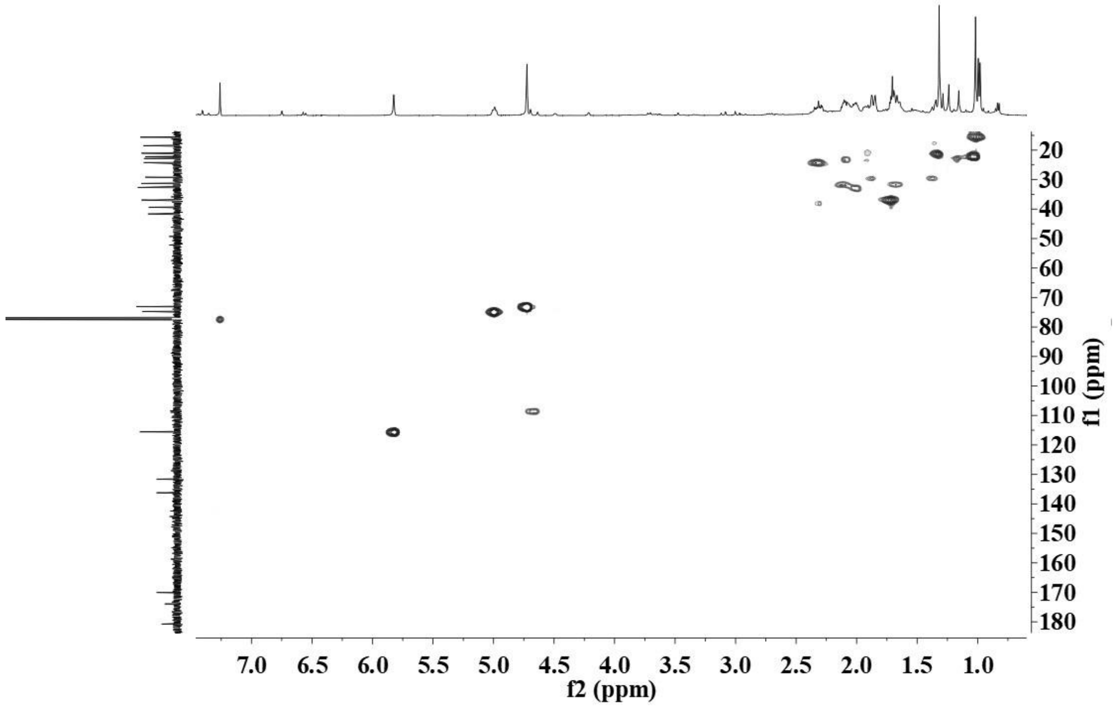

Figure S48. HSQC spectrum of **5** in $CDCl_3$

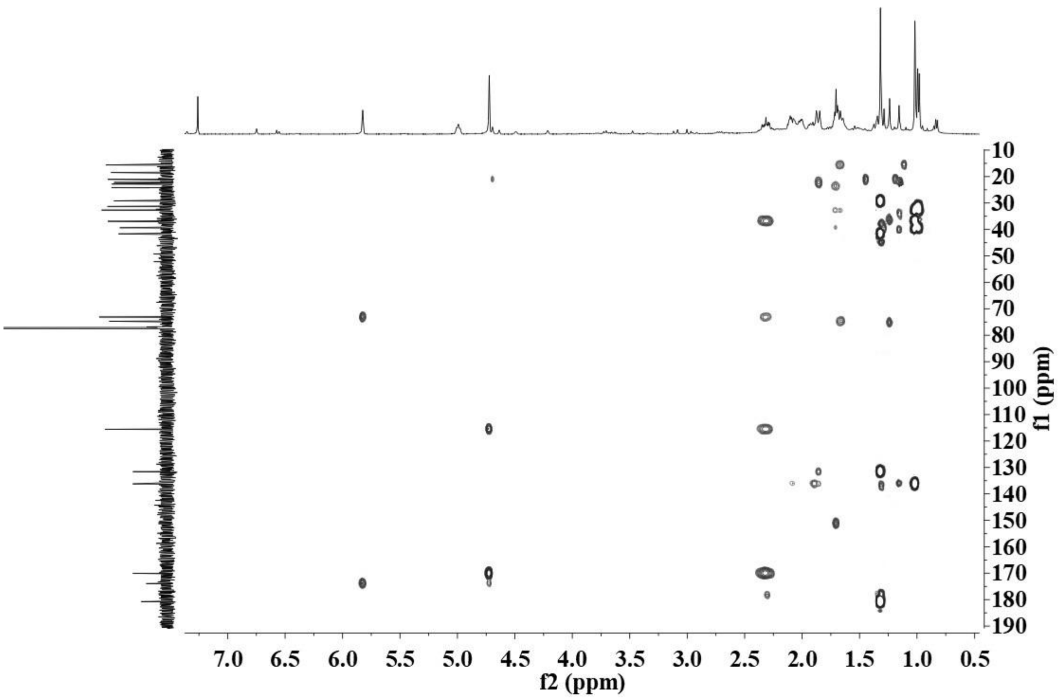

Figure S49. HMBC spectrum of **5** in $CDCl_3$

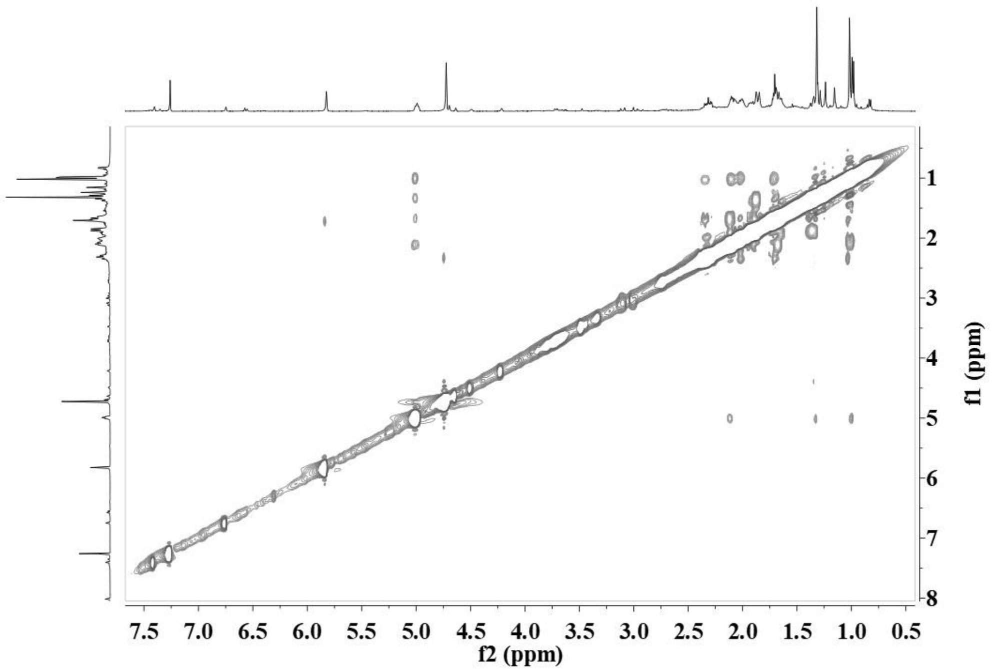

Figure S50. NOESY spectrum of **5** in $CDCl_3$

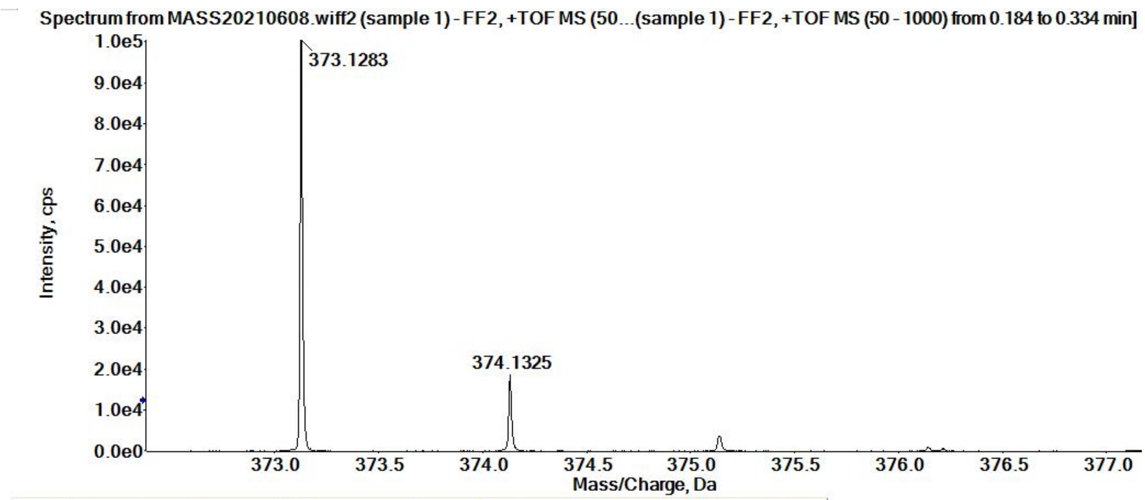

Hit	Formula	m/z	RDB	ppm	MS Rank	MSMS ppm	MSMS Rank	Found
1	C20H2007	373.1282	11.0	0.3	1			NA/NA

Figure S51. HR-ESI-MS of **6**

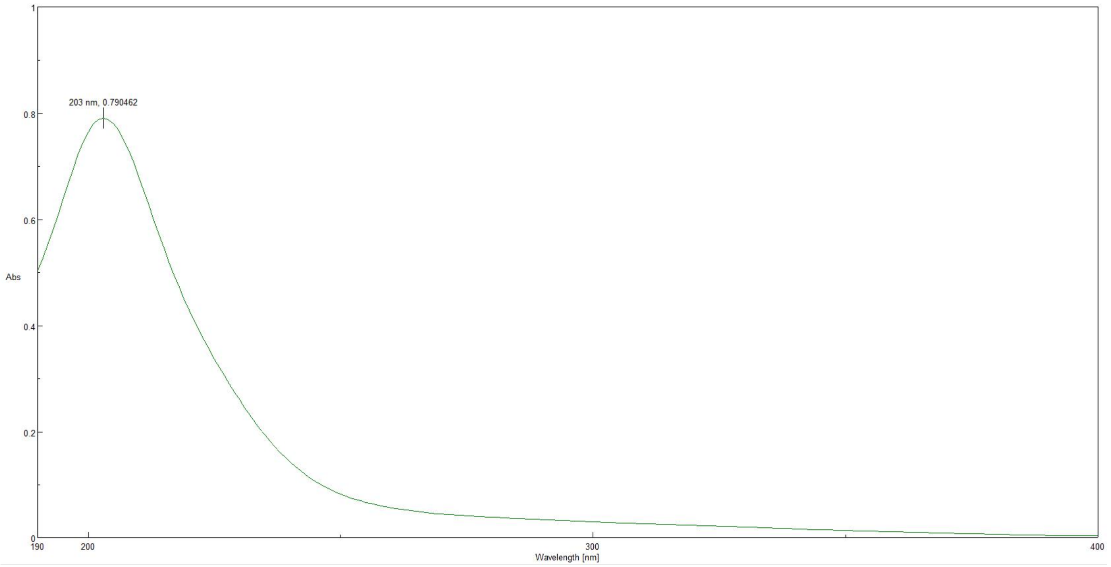

Figure S52. UV spectrum of **6** (CH_3OH)

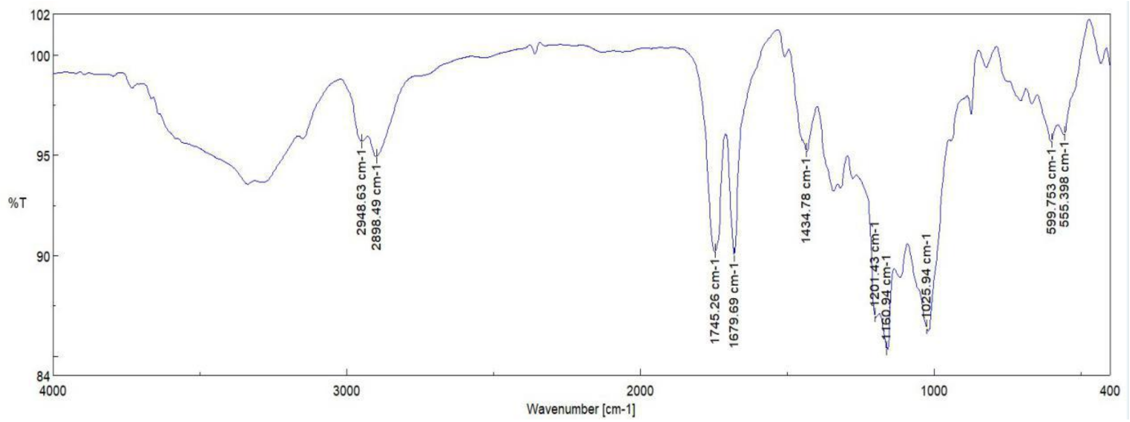

Figure S53. IR spectrum of **6** (KBr disc)

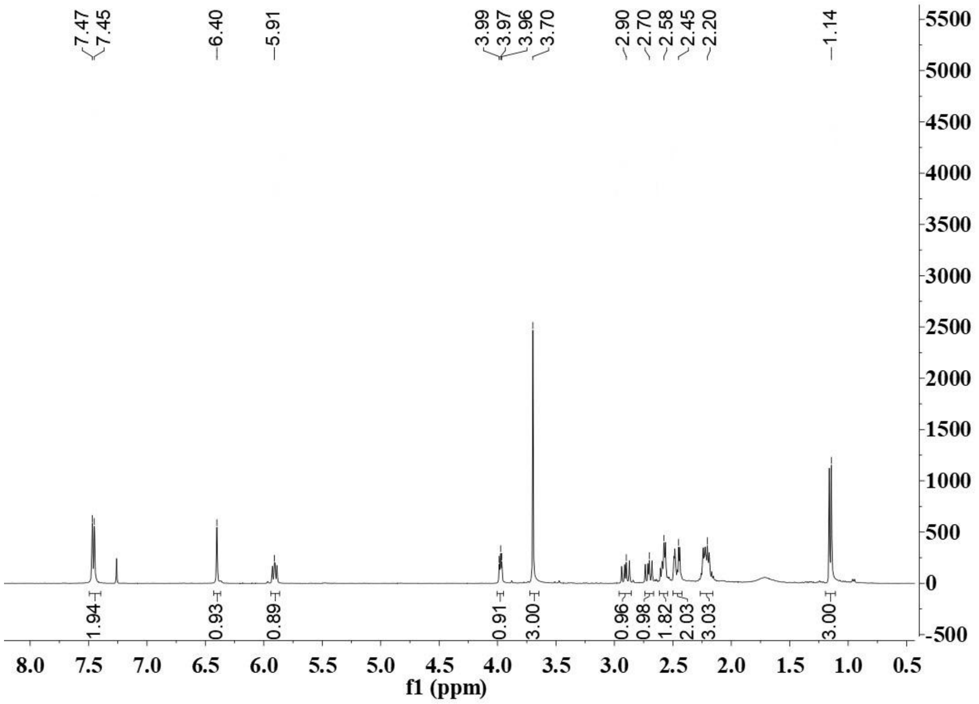

Figure S54. ^{1}H NMR spectrum of **6** in $CDCl_3$ (400 MHz)

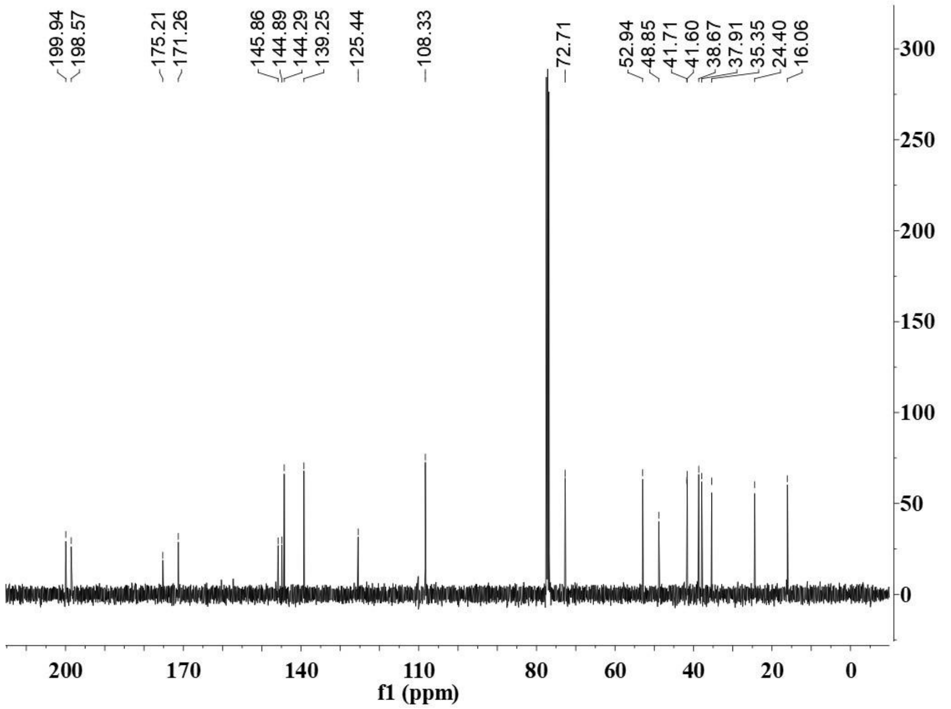

Figure S55. ^{13}C NMR spectrum of **6** in $CDCl_3$ (100 MHz)

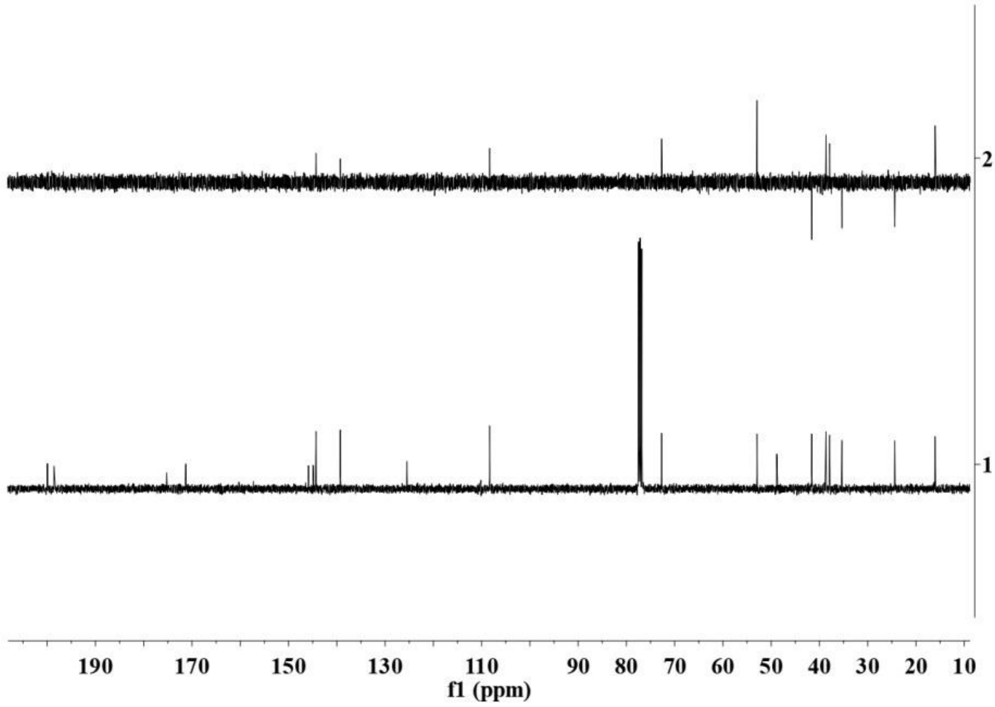

Figure S56. DEPT-135 spectrum of **6** in $CDCl_3$ (100 MHz)

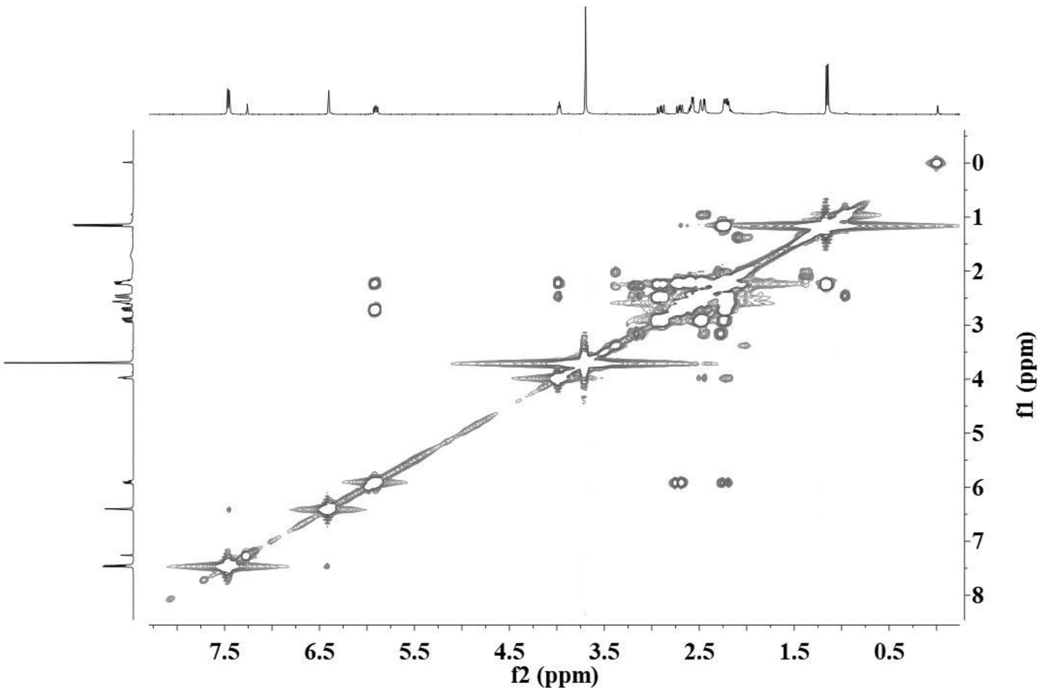

Figure S57. ^1H-^1H COSY spectrum of **6** in $CDCl_3$

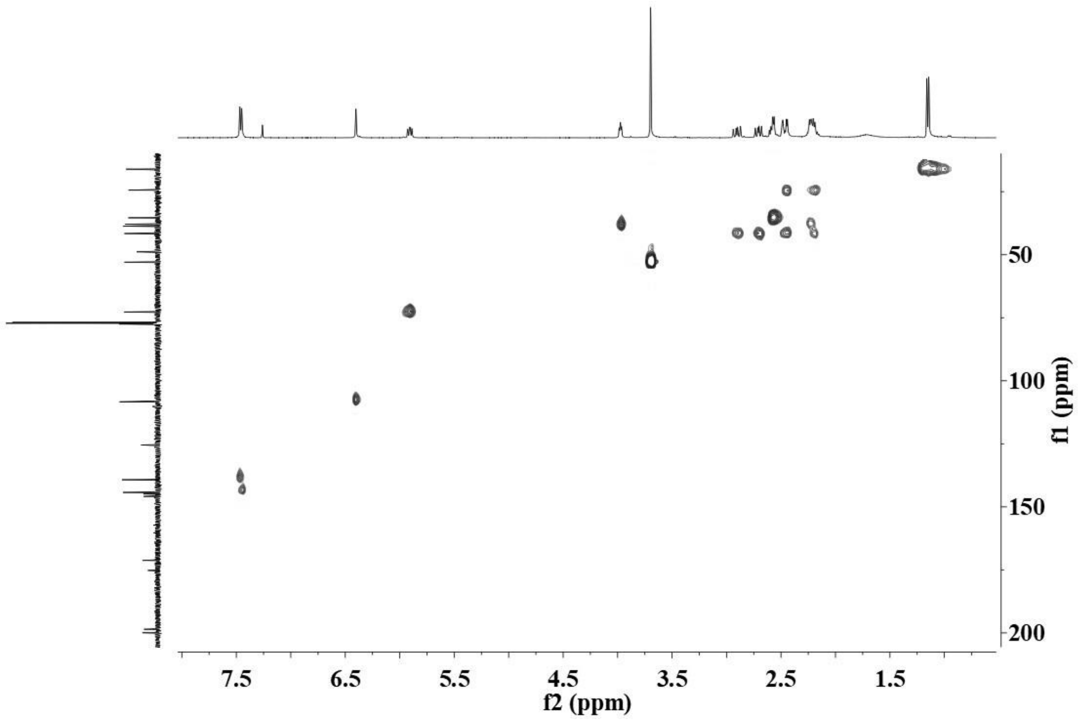

Figure S58. HSQC spectrum of **6** in $CDCl_3$

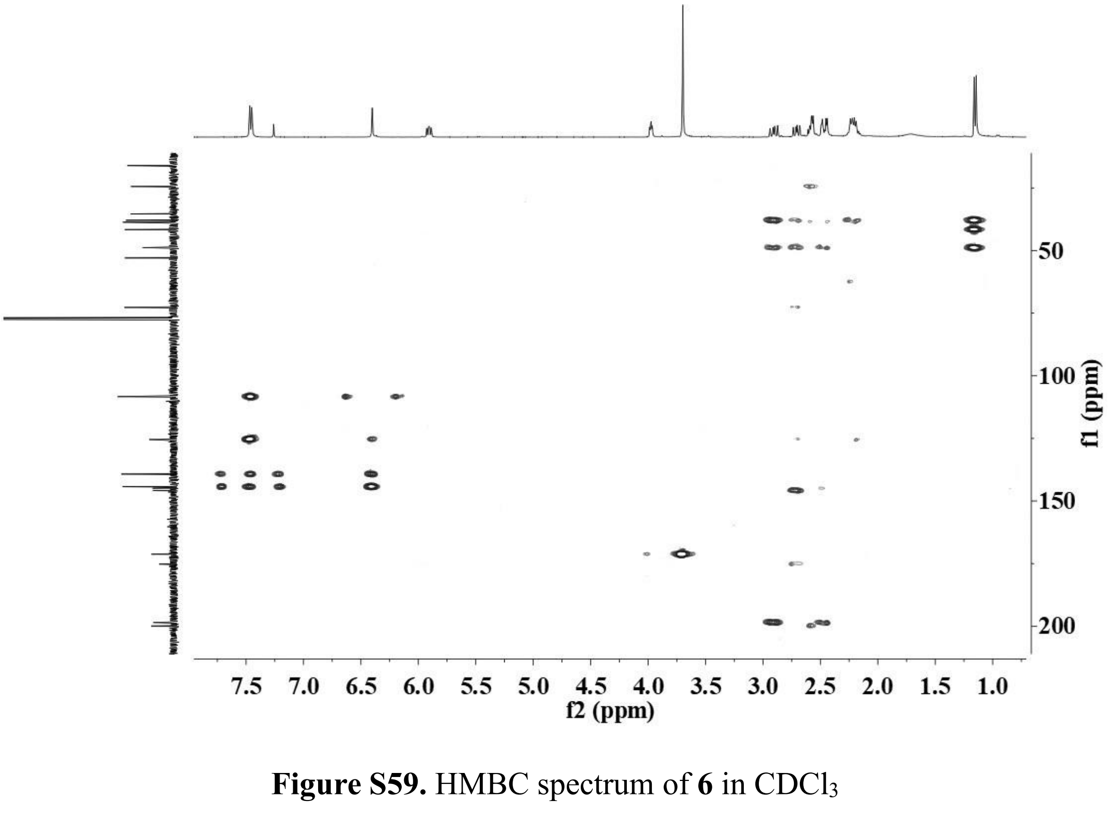

Figure S59. HMBC spectrum of **6** in $CDCl_3$

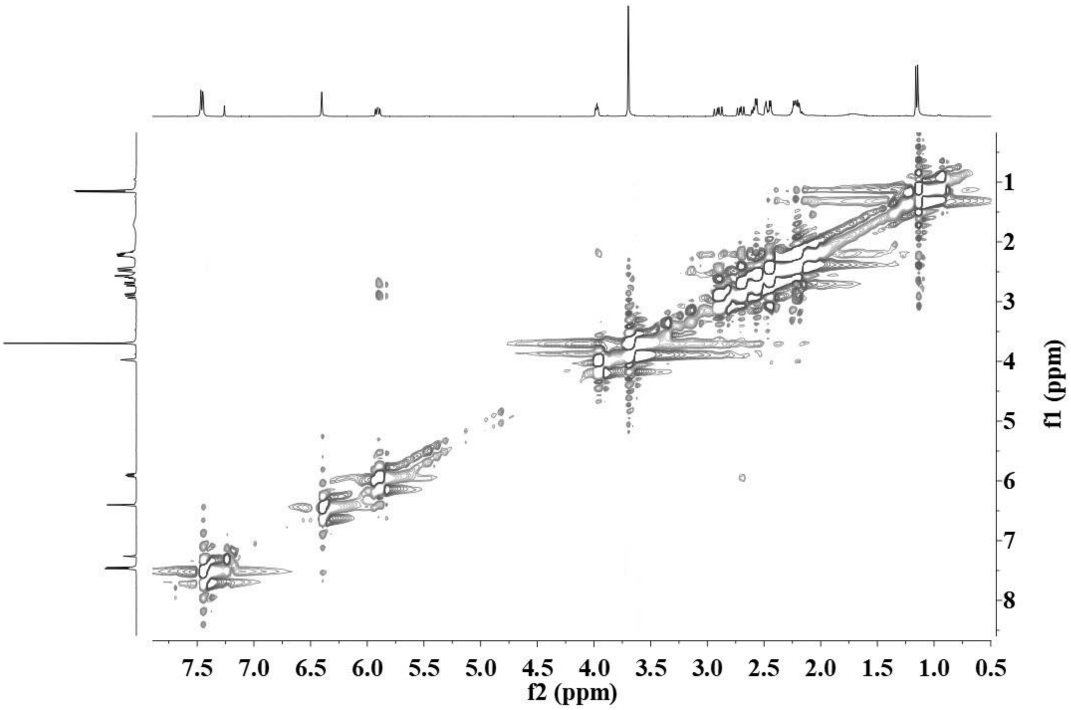

Figure S60. NOESY spectrum of **6** in $CDCl_3$

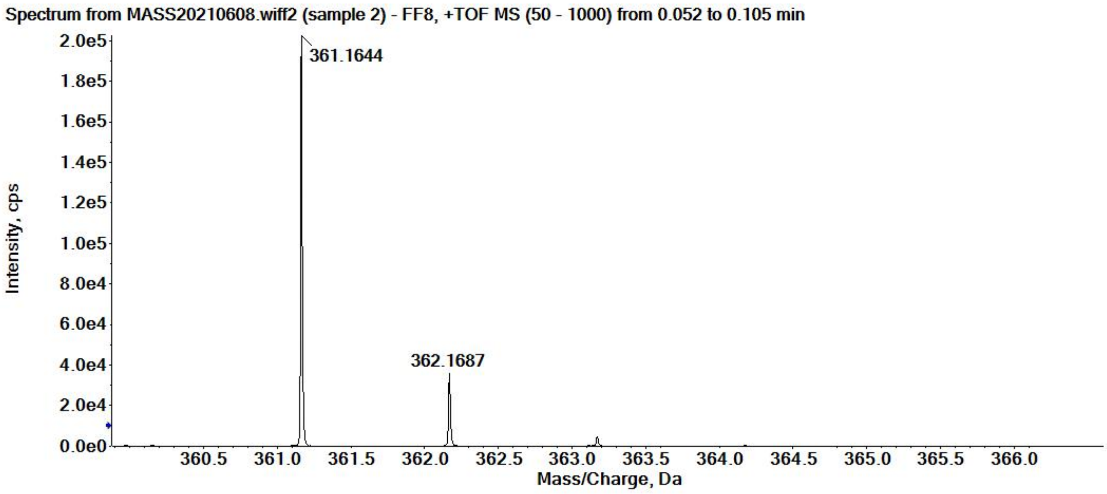

Hit	Formula	m/z	RDB	ppm	MS Rank	MSMS ppm	MSMS Rank	Found
1	C20H24O6	361.1646	9.0	−0.5	1			NA/NA

Figure S61. HR-ESI-MS of **7**

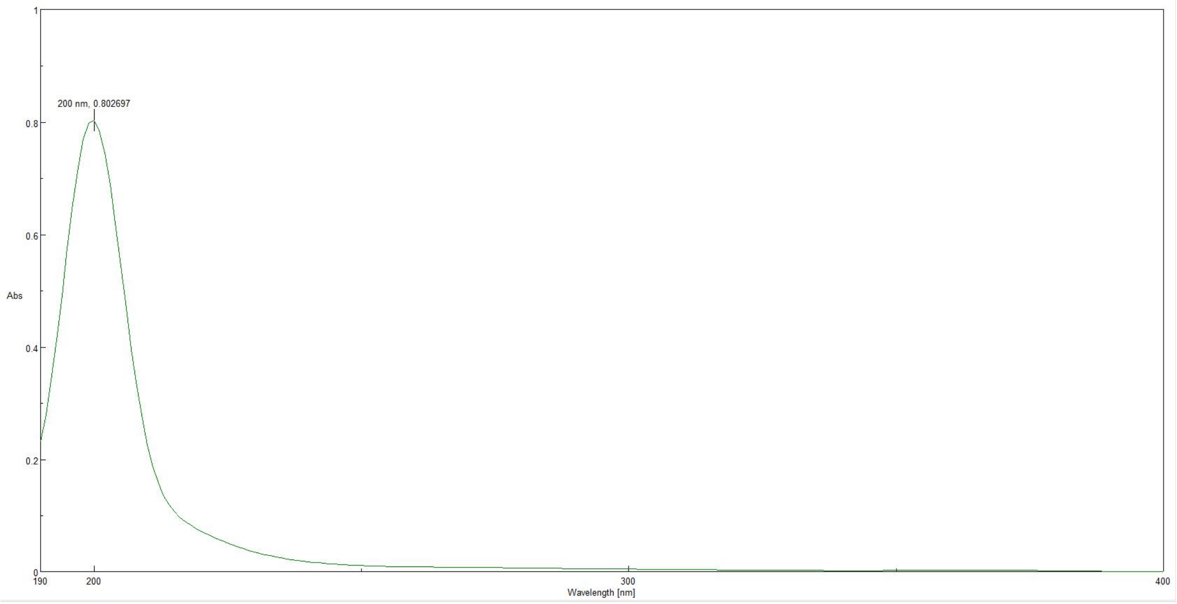

Figure S62. UV spectrum of **7** (CH_3OH)

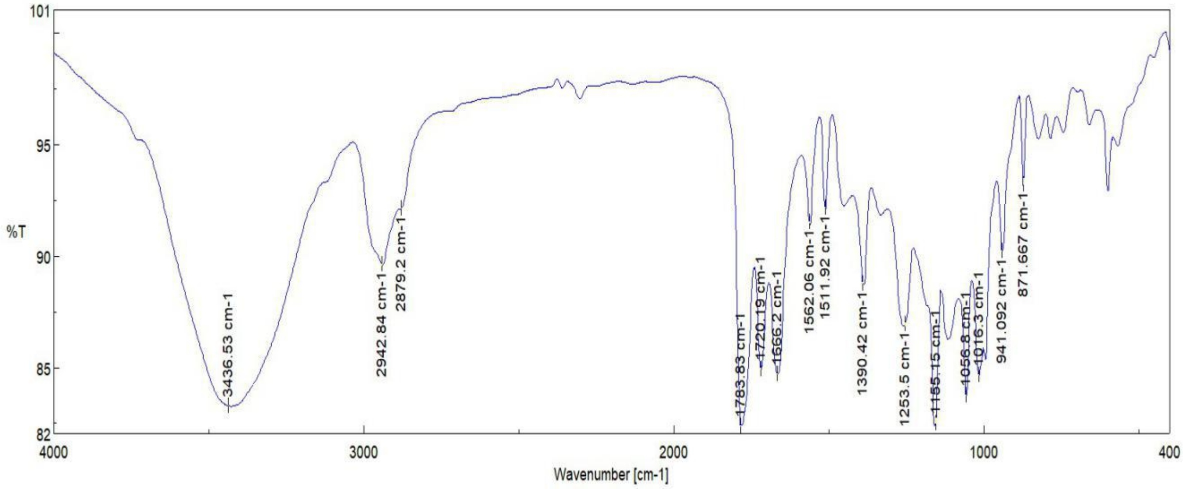

Figure S63. IR spectrum of **7** (KBr disc)

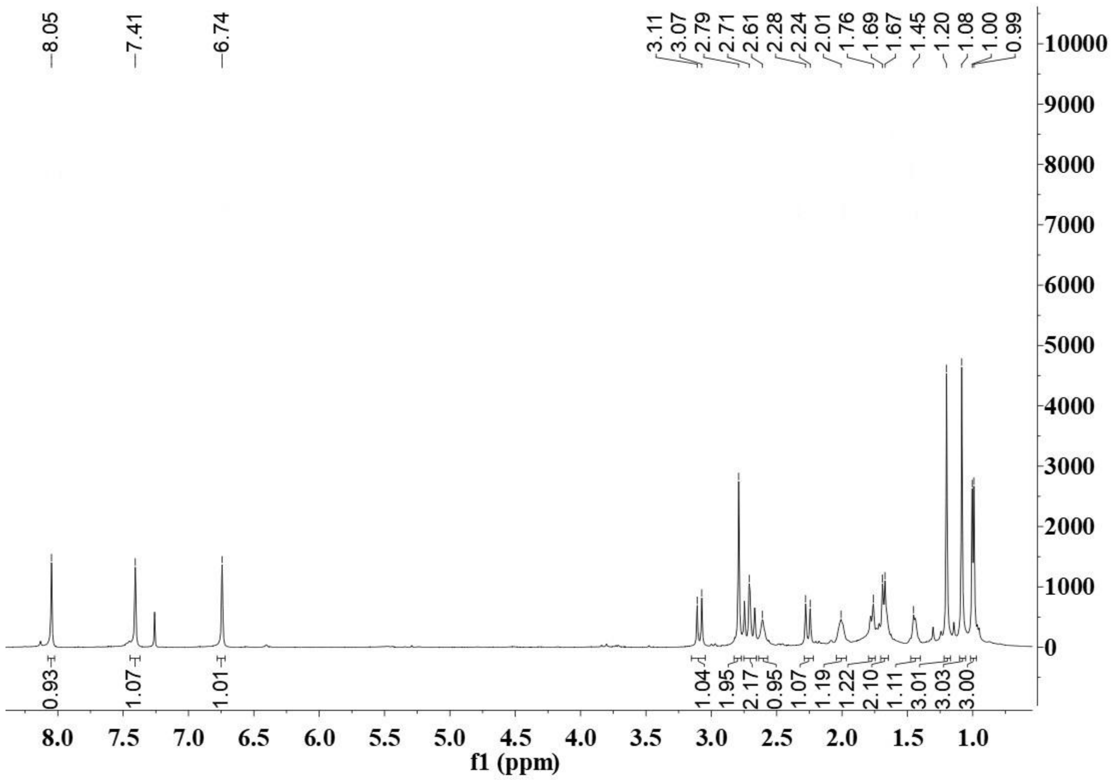

Figure S64. ^{1}H NMR spectrum of **7** in $CDCl_3$ (400 MHz)

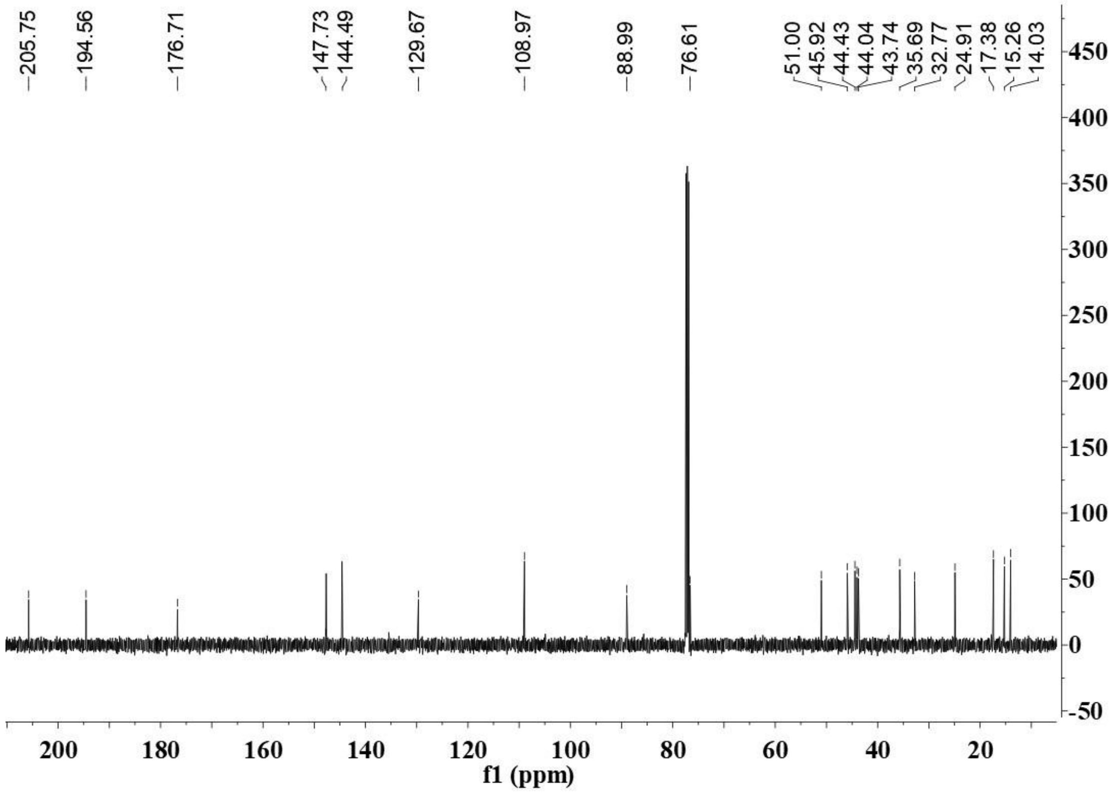

Figure S65. ^{13}C NMR spectrum of **7** in $CDCl_3$ (100 MHz)

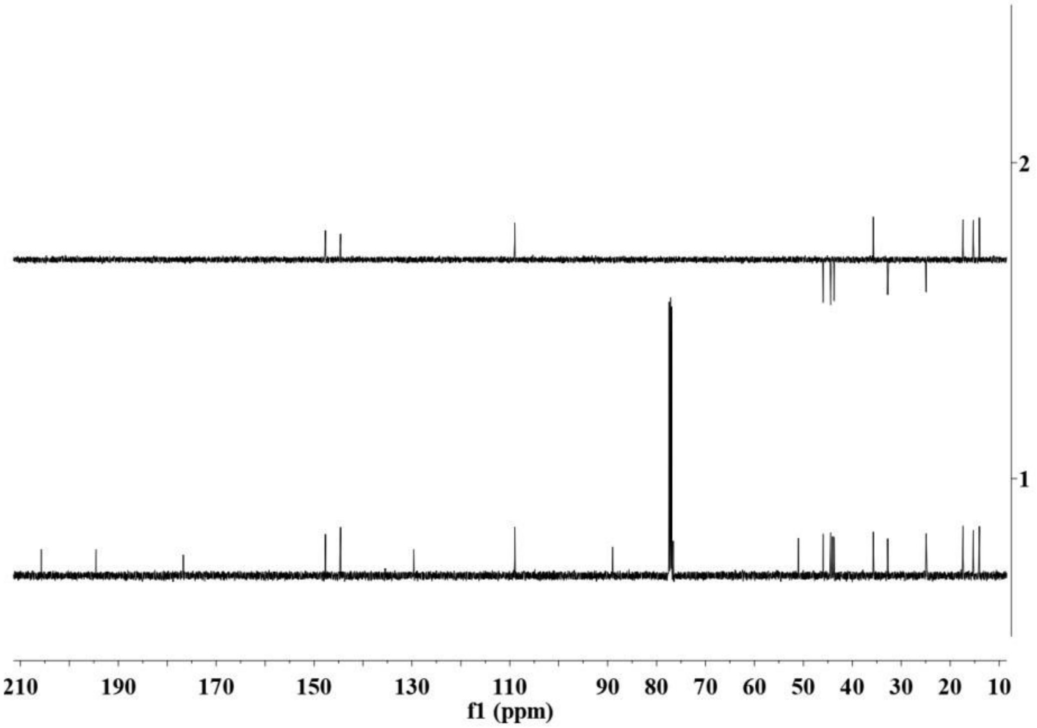

Figure S66. DEPT-135 spectrum of **7** in $CDCl_3$ (100 MHz)

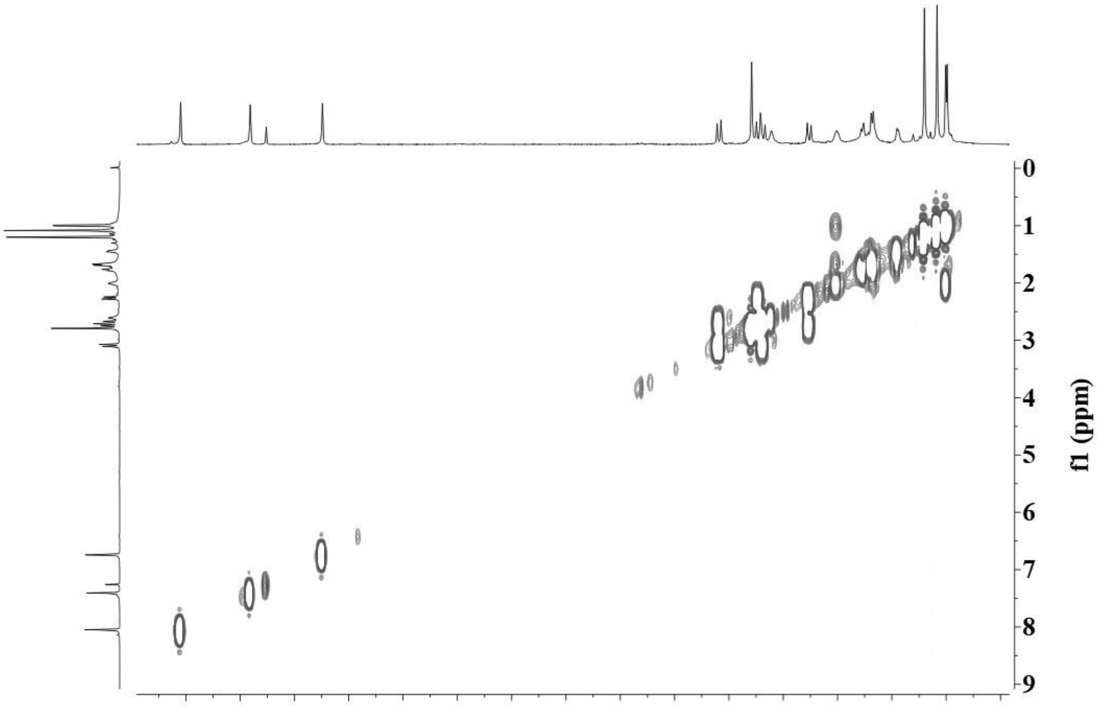

Figure S67. ^1H-^1H COSY spectrum of **7** in $CDCl_3$

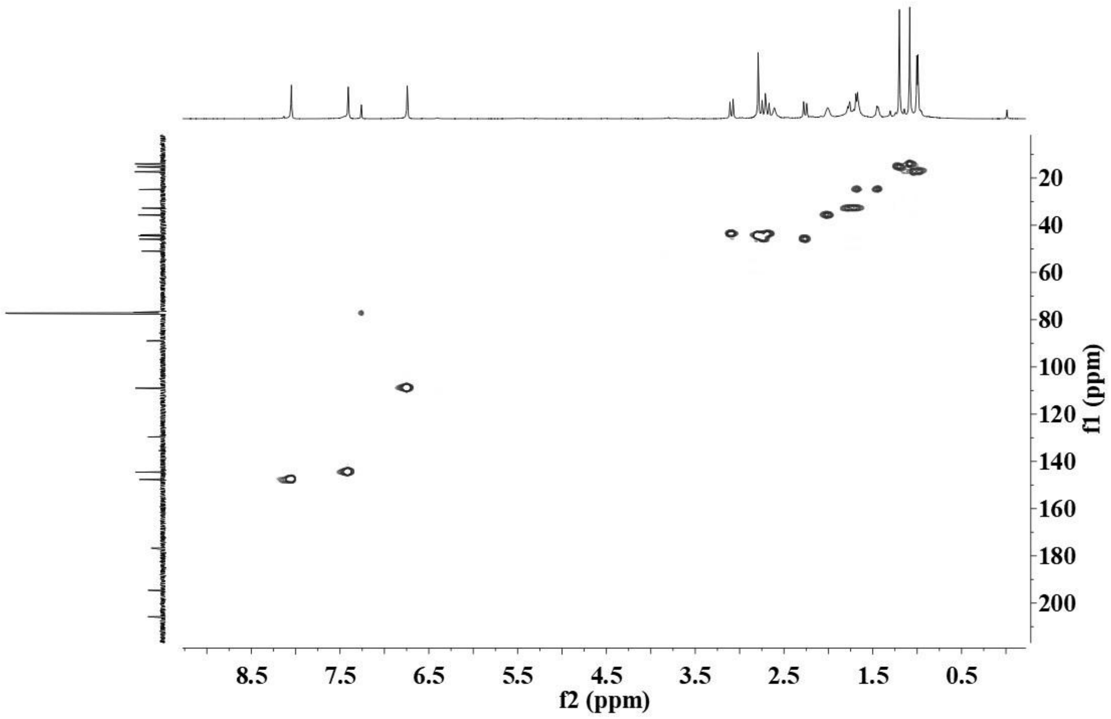

Figure S68. HSQC spectrum of **7** in $CDCl_3$

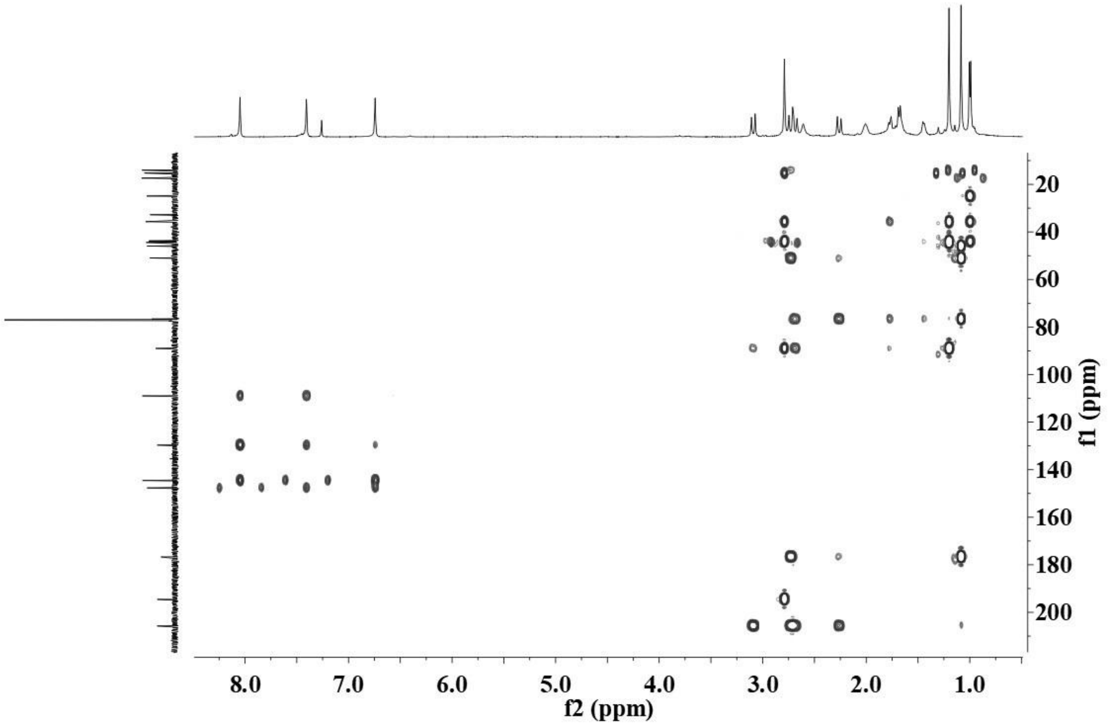

Figure S69. HMBC spectrum of **7** in $CDCl_3$

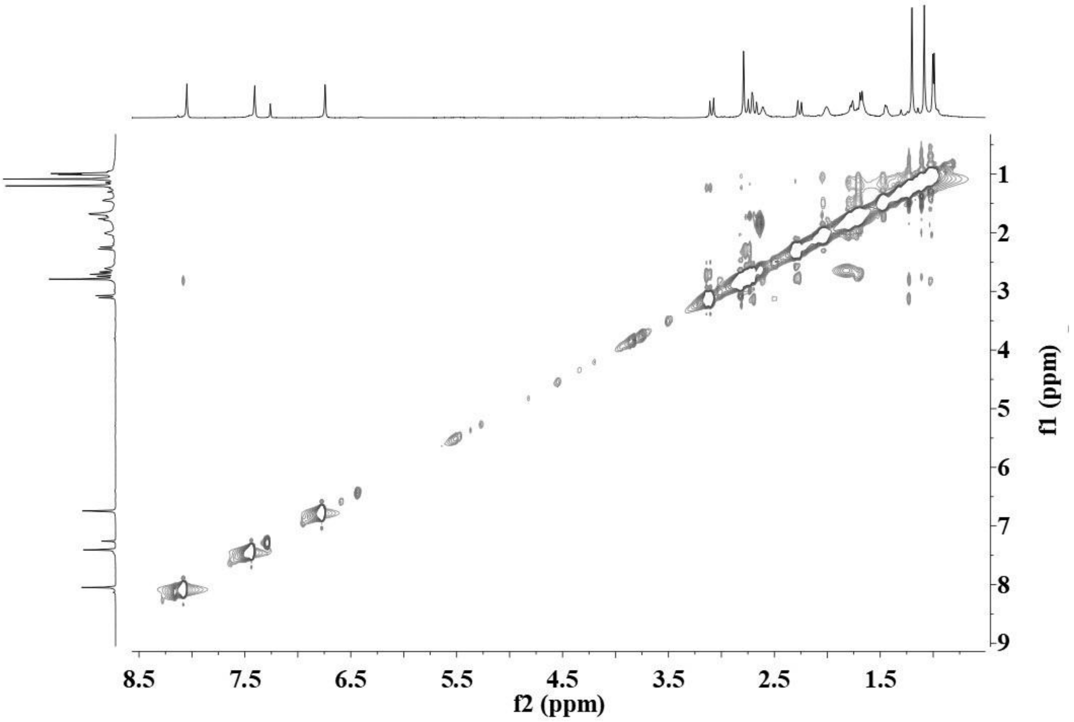

Figure S70. NOESY spectrum of **7** in $CDCl_3$

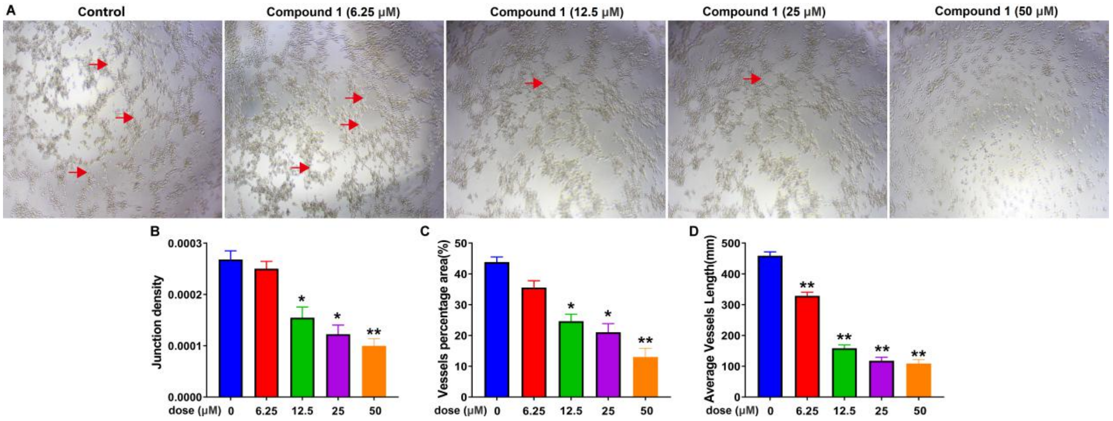

Figure S71. The inhibitory effect of compound **1** on angiogenesis. (A) Bright field images of HUVECs seeded on Matrigel for 24 h after treatment at different doses of compound **1**; red arrows indicated the cavities formation during angiogenesis. (B), (C), (D) Quantitative analysis on junction densities, vessels percentage areas and average vessels lengths, respectively. Data are expressed as mean ± SD of three independent experiments. $^{*}P < 0.05$, $^{**}P < 0.05$, $^{***}P < 0.001$ compared with the control group.

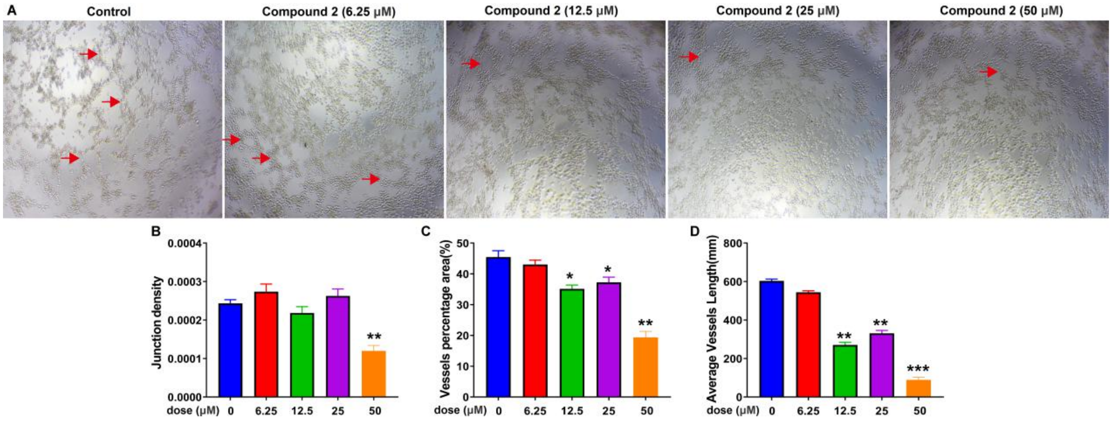

Figure S72. The inhibitory effect of compound **2** on angiogenesis.

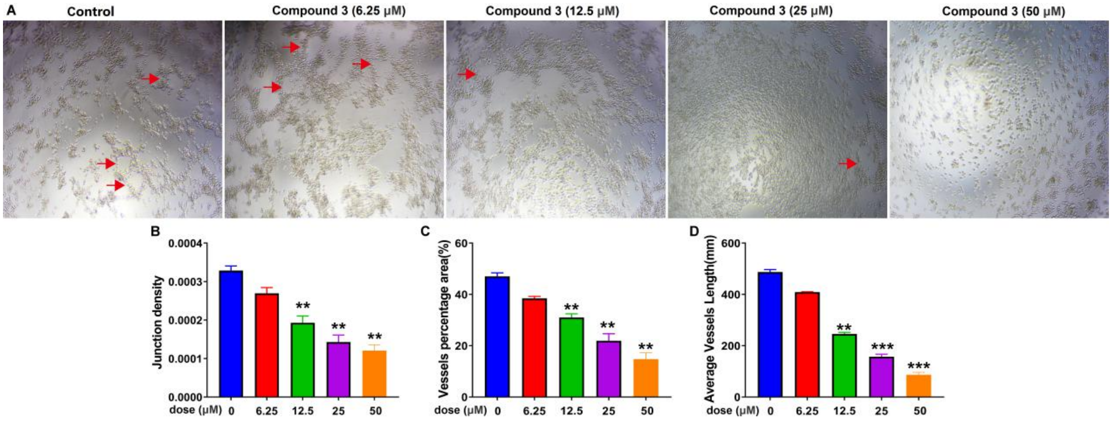

Figure S73. The inhibitory effect of compound **3** on angiogenesis.

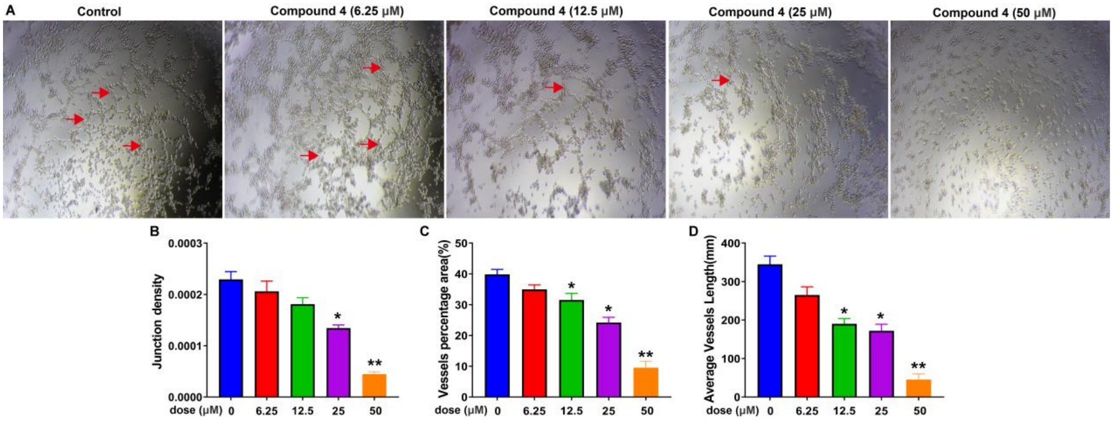

Figure S74. The inhibitory effect of compound **4** on angiogenesis.

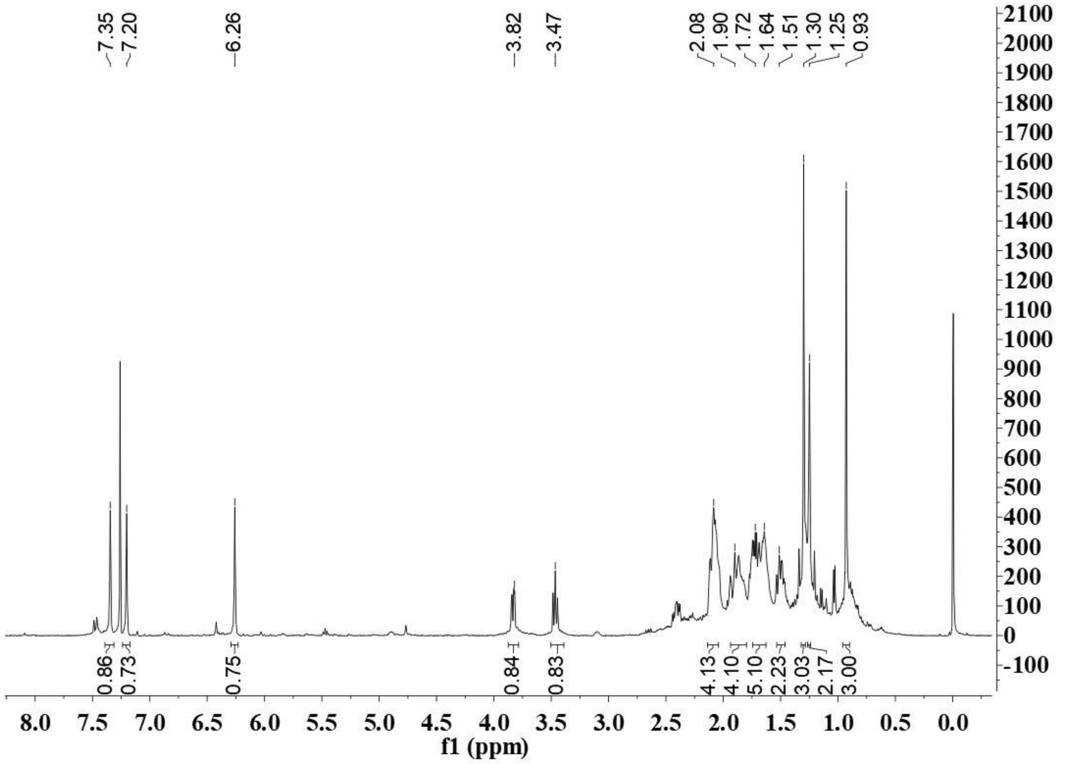

Figure S75. ^{1}H NMR spectrum of **8** in $CDCl_3$ (400MHz)

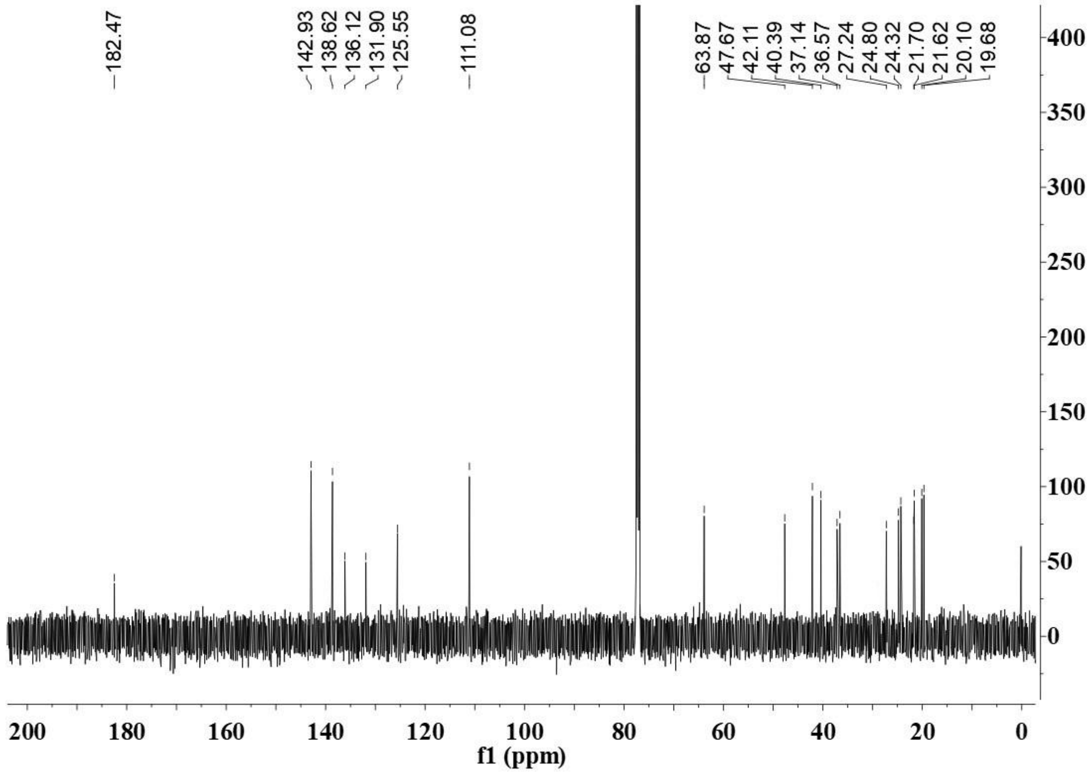

Figure S76. ^{13}C NMR spectrum of **8** in $CDCl_3$ (100MHz)

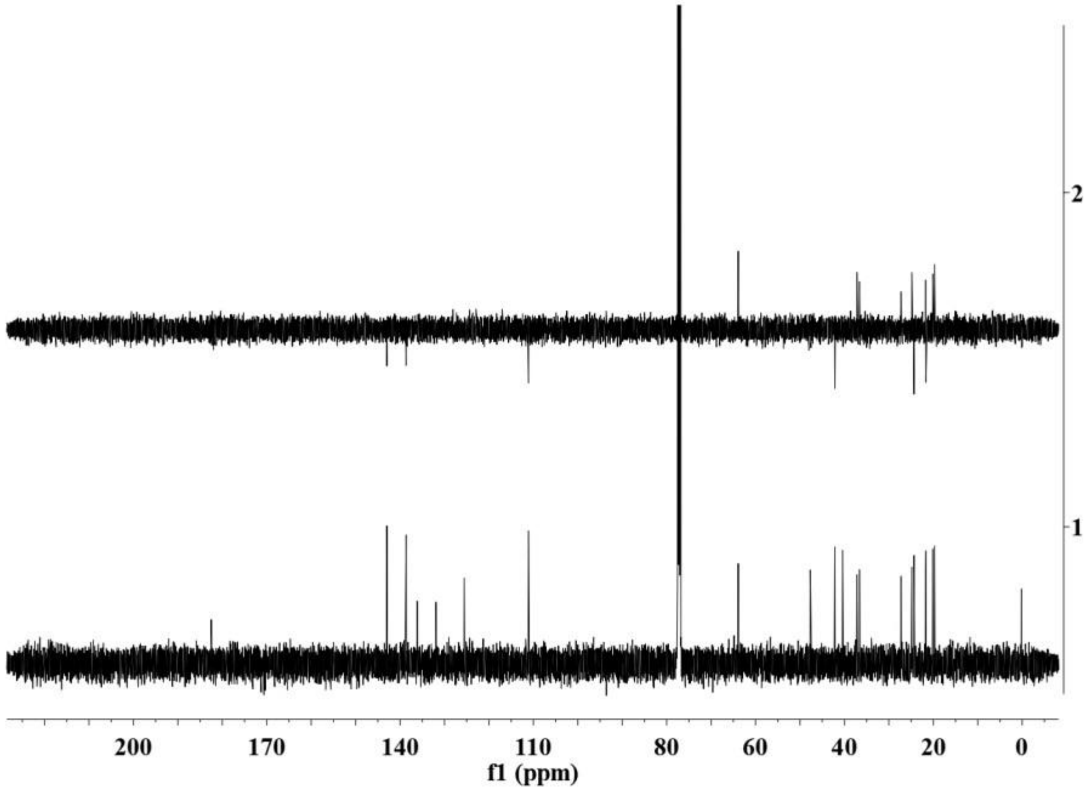

Figure S77. DEPT-135 spectrum of **8** in $CDCl_3$ (100MHz)

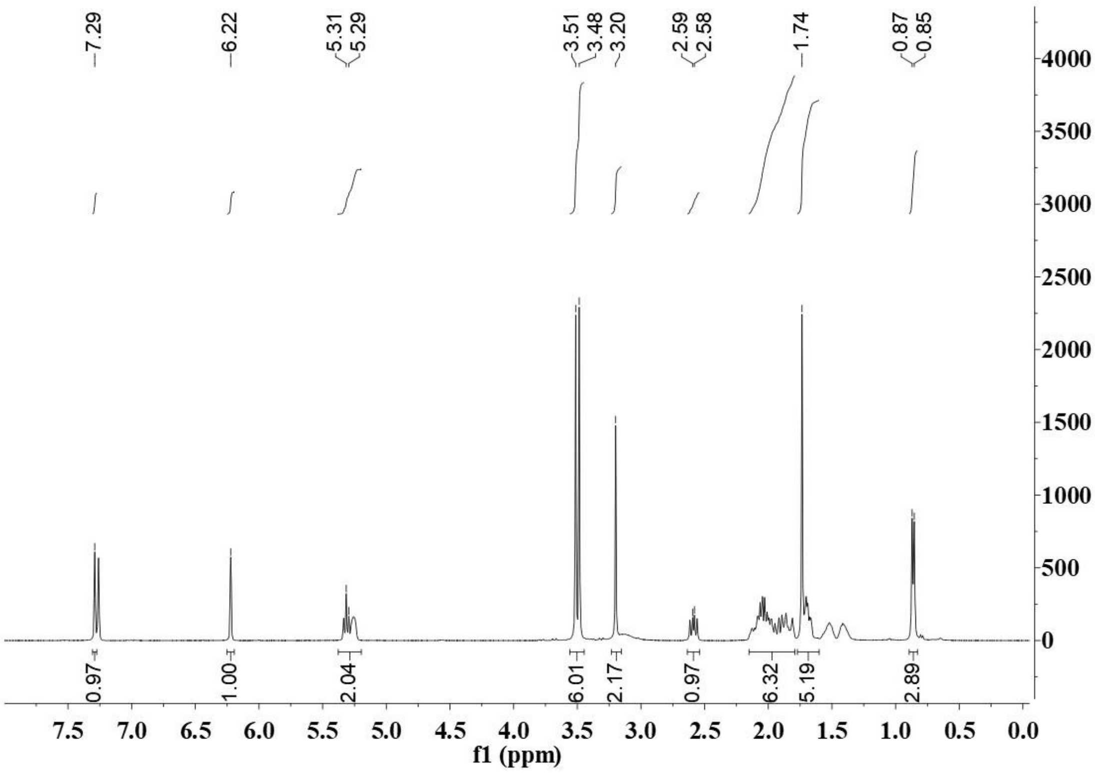

Figure S78. ^{1}H NMR spectrum of **9** in $CDCl_3$ (400MHz)

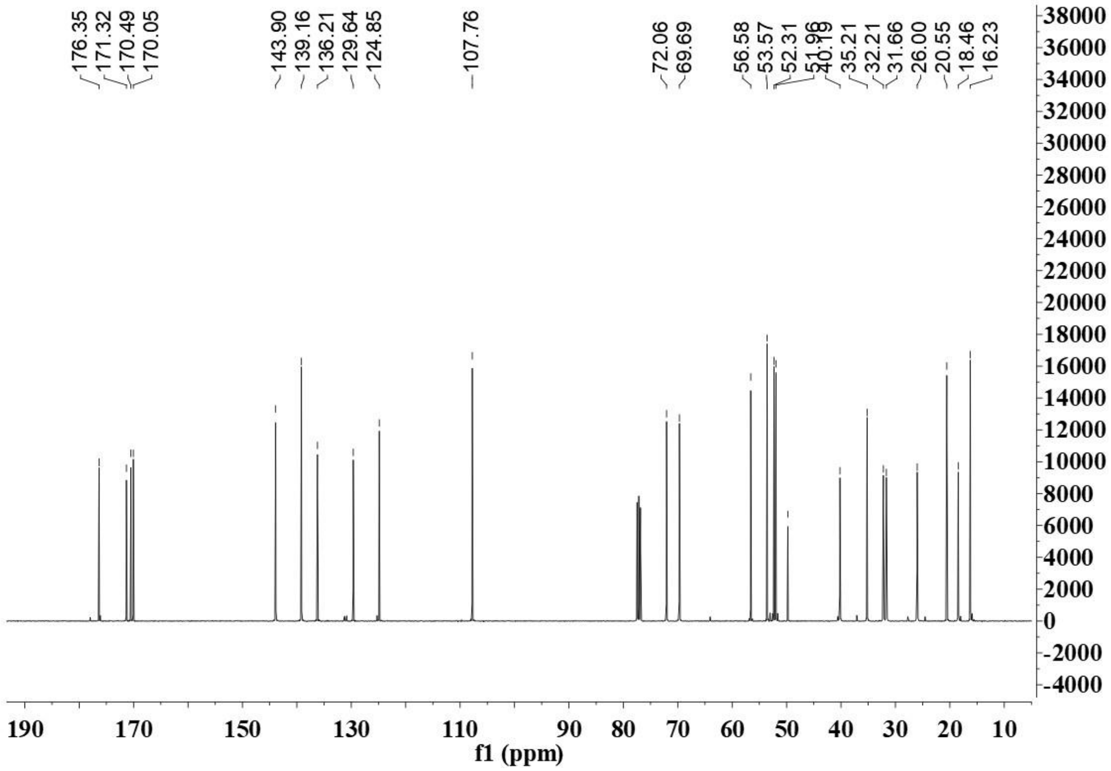

Figure S79. ^{13}C NMR spectrum of **9** in $CDCl_3$ (100MHz)

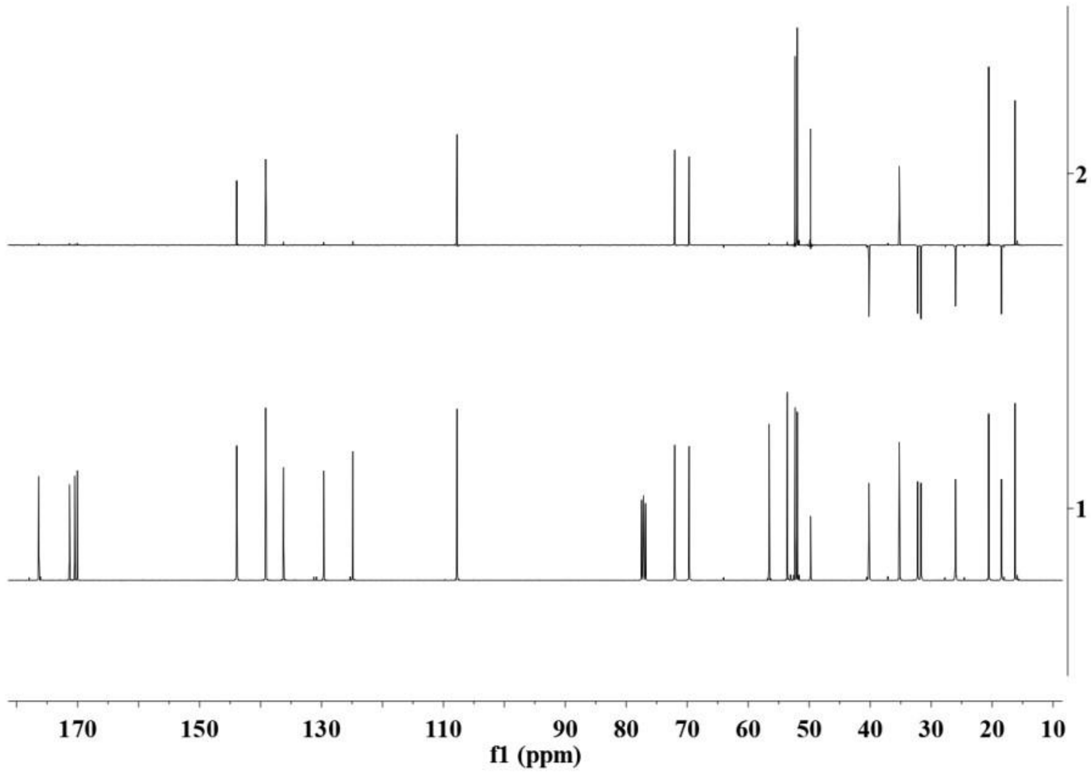

Figure S80. DEPT-135 spectrum of **9** in $CDCl_3$ (100MHz)

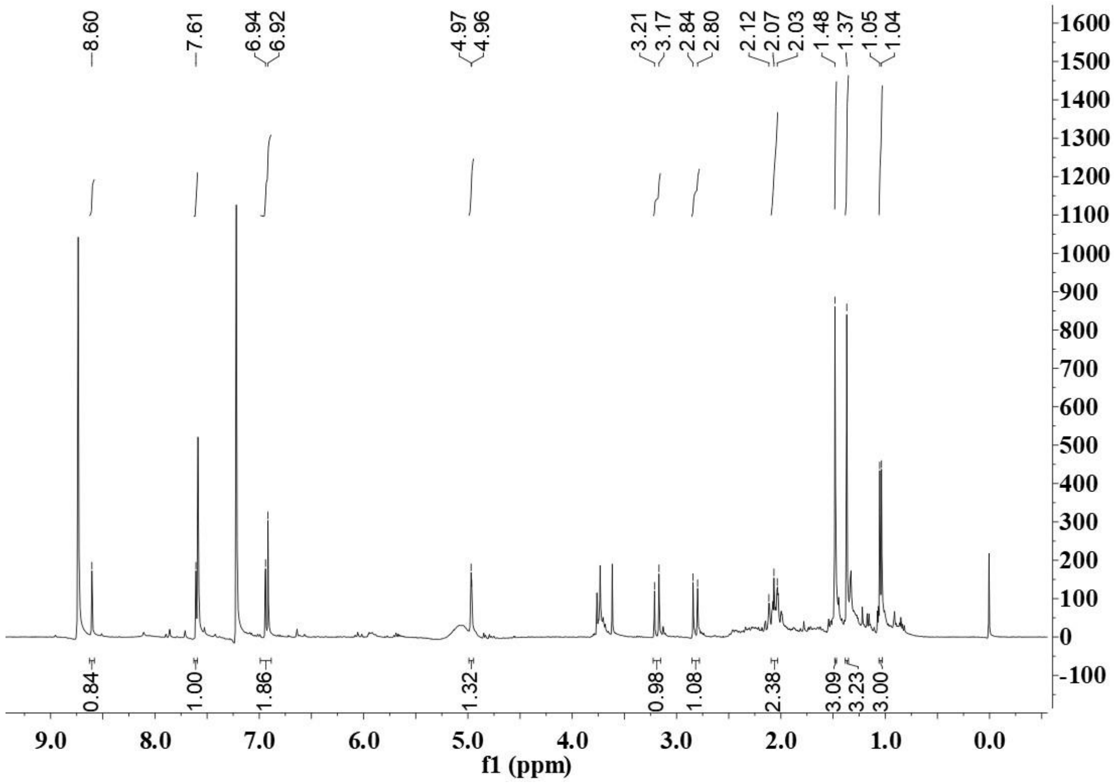

Figure S81. ^{1}H NMR spectrum of **10** in pyridine-d_5 (400MHz)

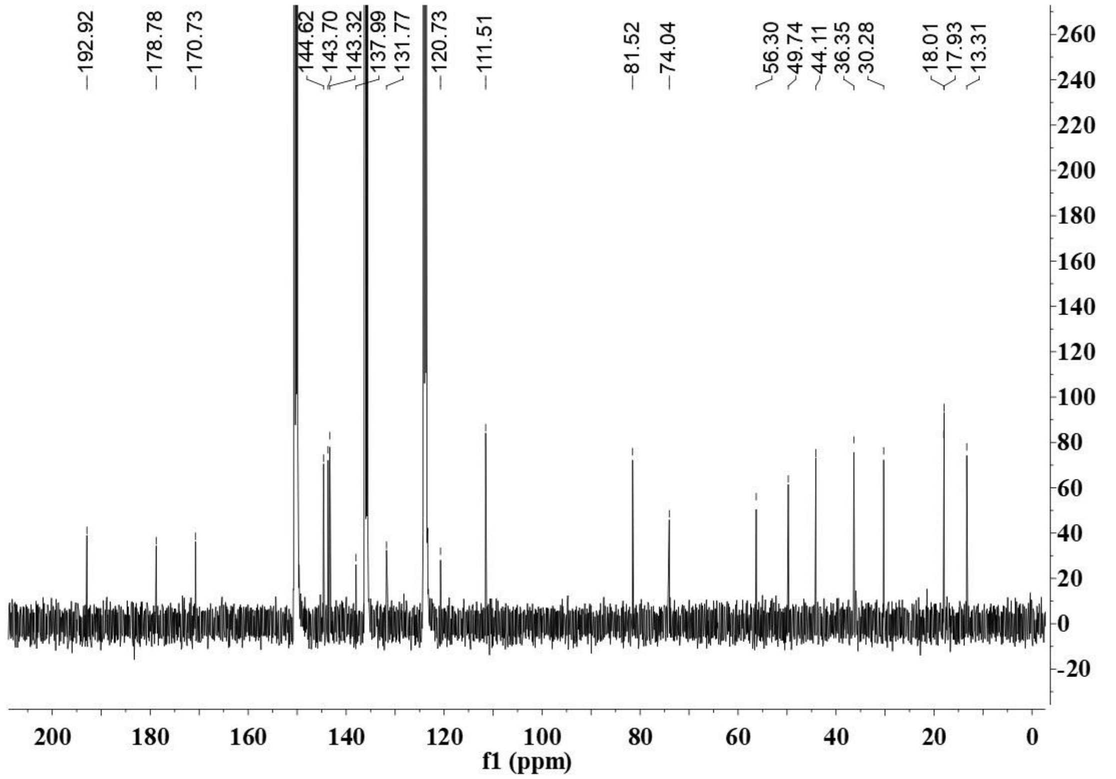

Figure S82. ^{13}C NMR spectrum of **10** in pyridine-d_5 (100MHz)

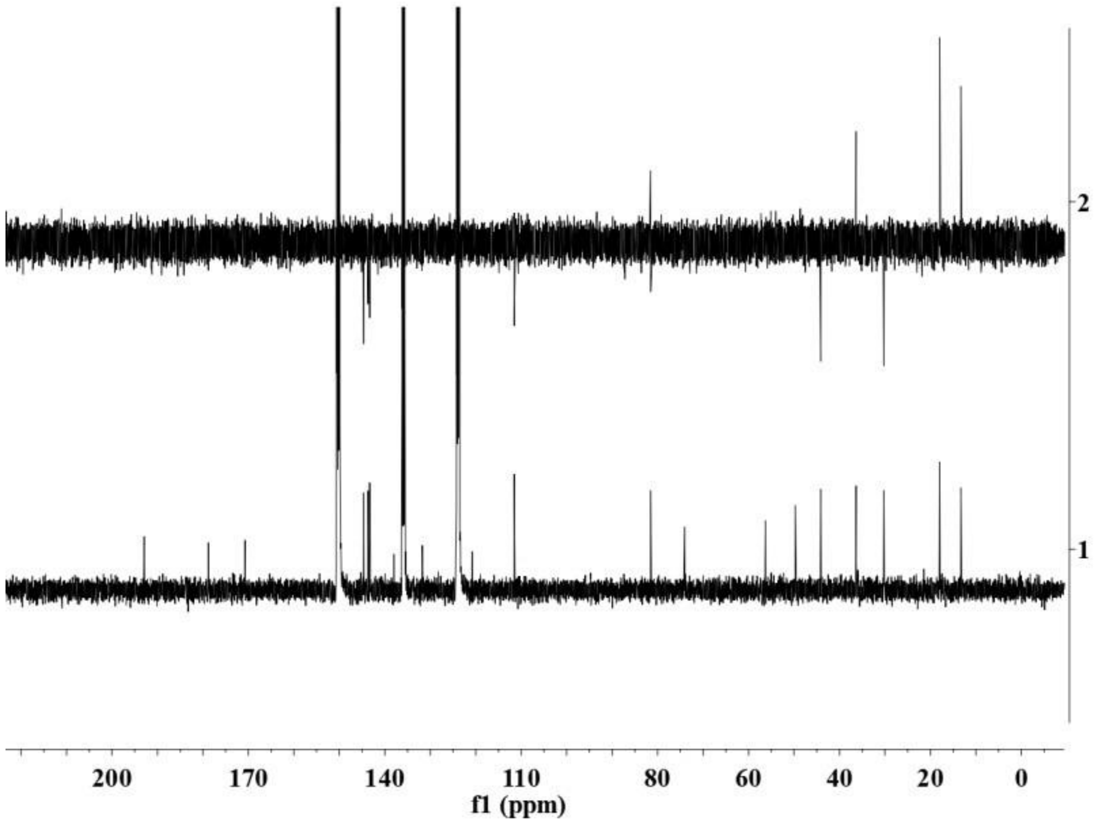

Figure S83. DEPT-135 spectrum of **10** in pyridine-d_5 (100MHz)

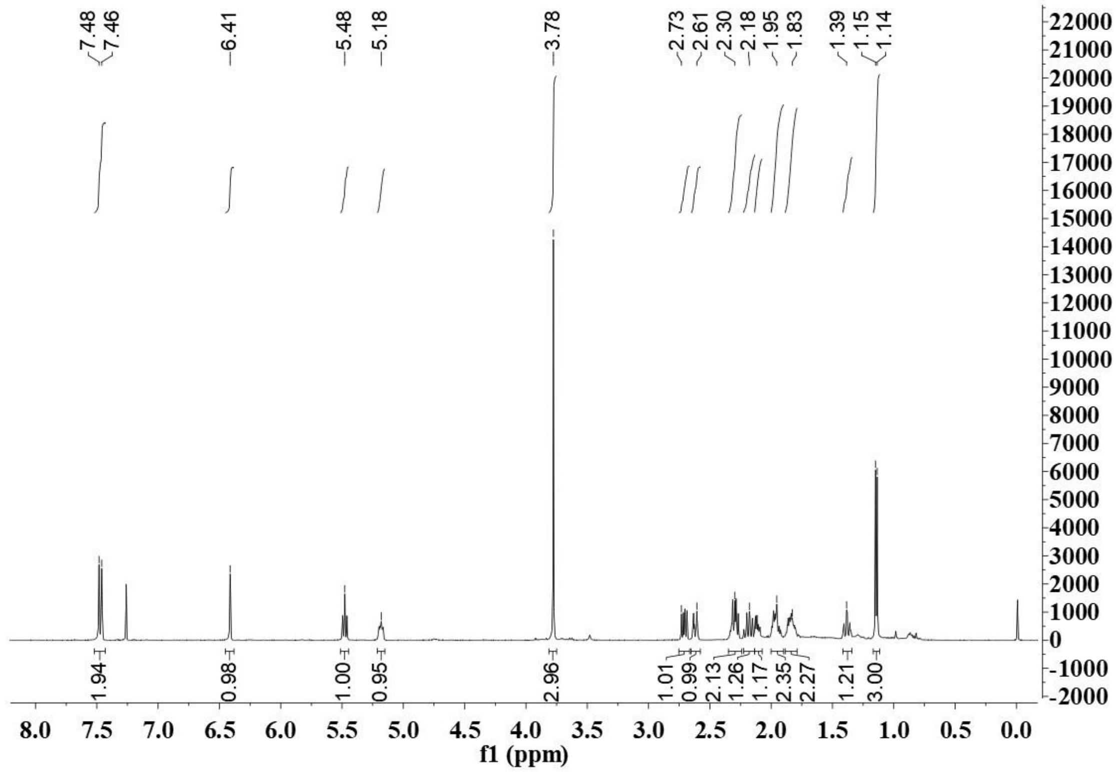

Figure S84. ^{1}H NMR spectrum of **11** in $CDCl_3$ (400MHz)

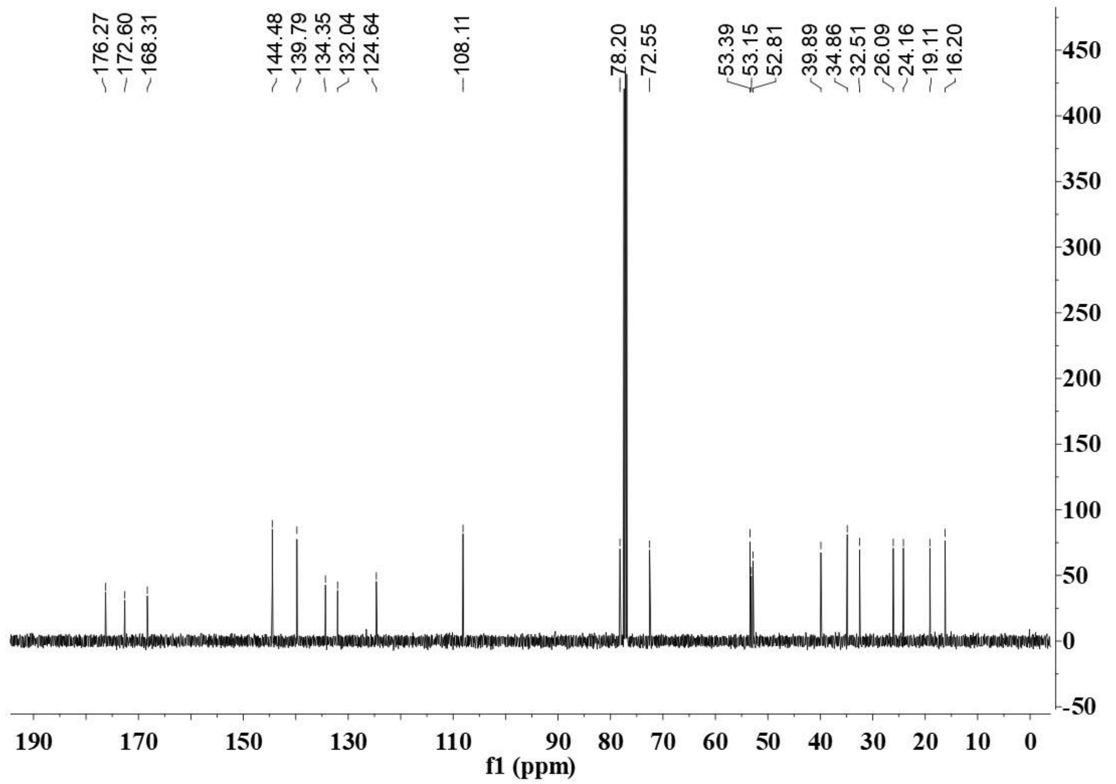

Figure S85. ^{13}C NMR spectrum of **11** in $CDCl_3$ (100MHz)

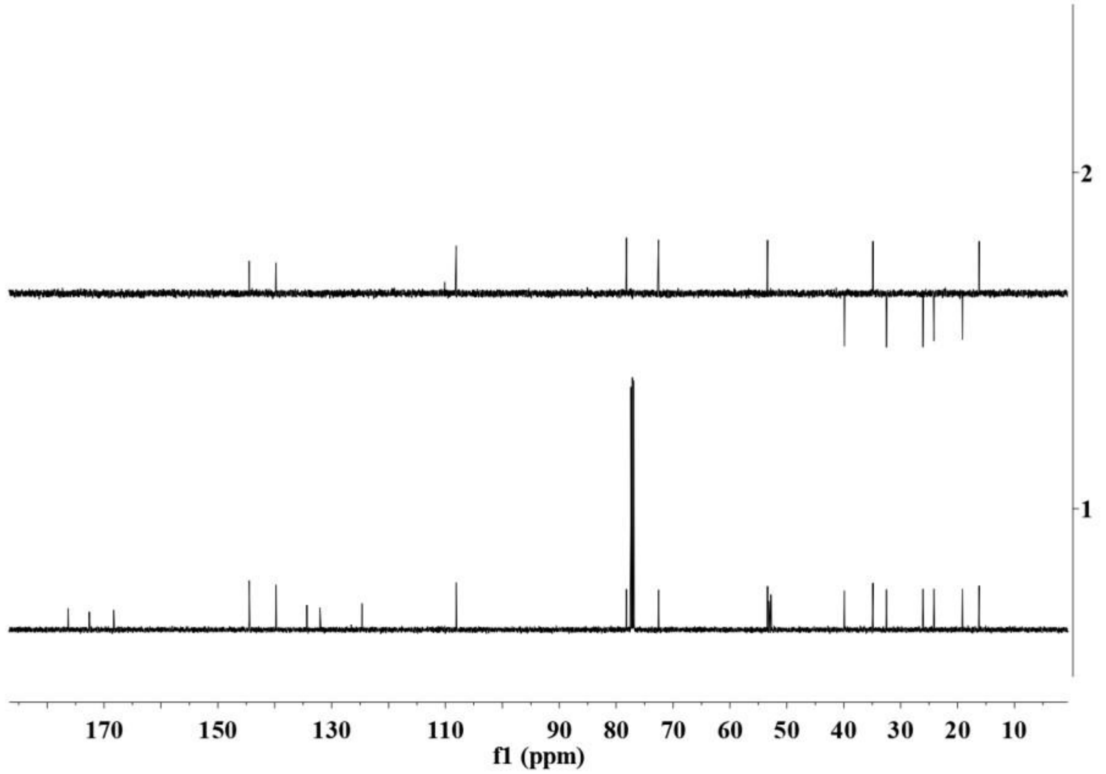

Figure S86. DEPT-135 spectrum of **11** in $CDCl_3$ (100MHz)

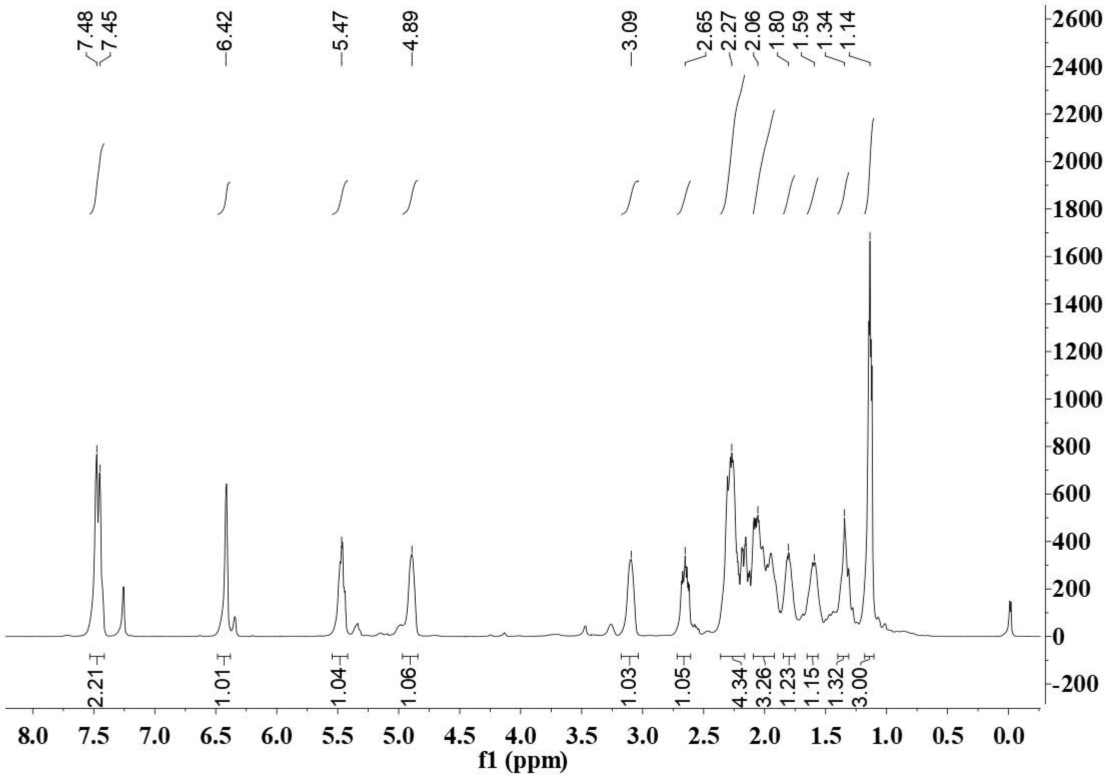

Figure S87. ^{1}H NMR spectrum of **12** in $CDCl_3$ (400MHz)

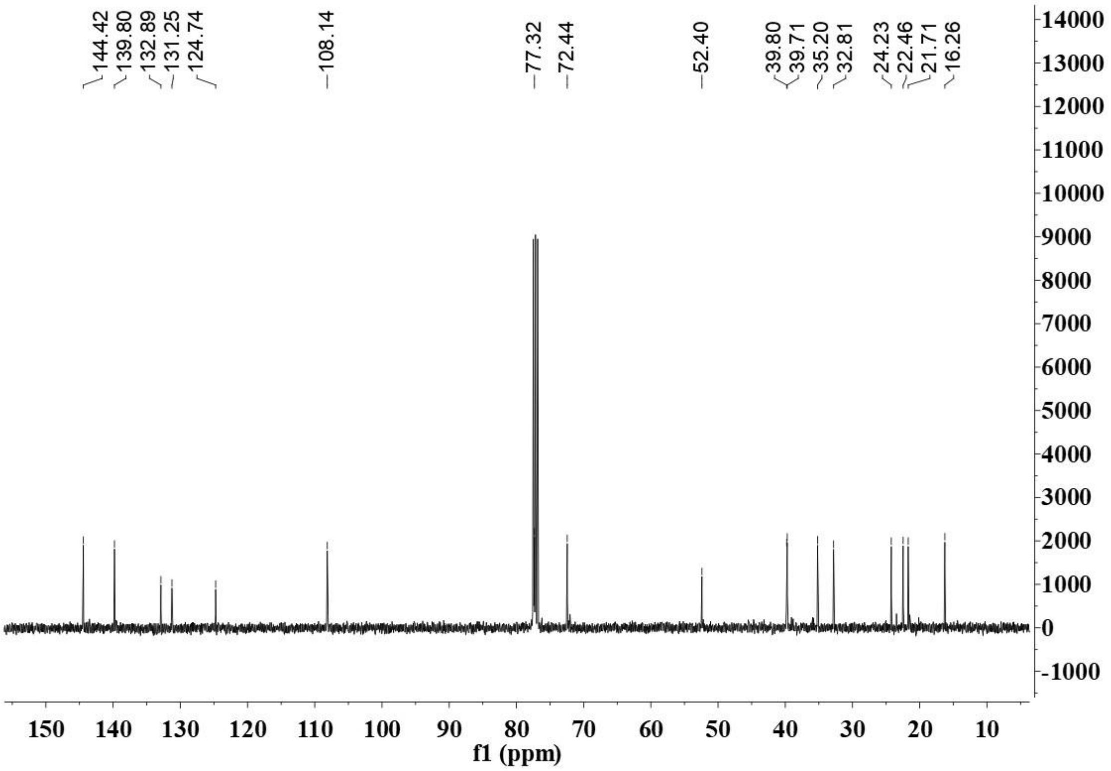

Figure S88. ^{13}C NMR spectrum of **12** in $CDCl_3$ (100MHz)

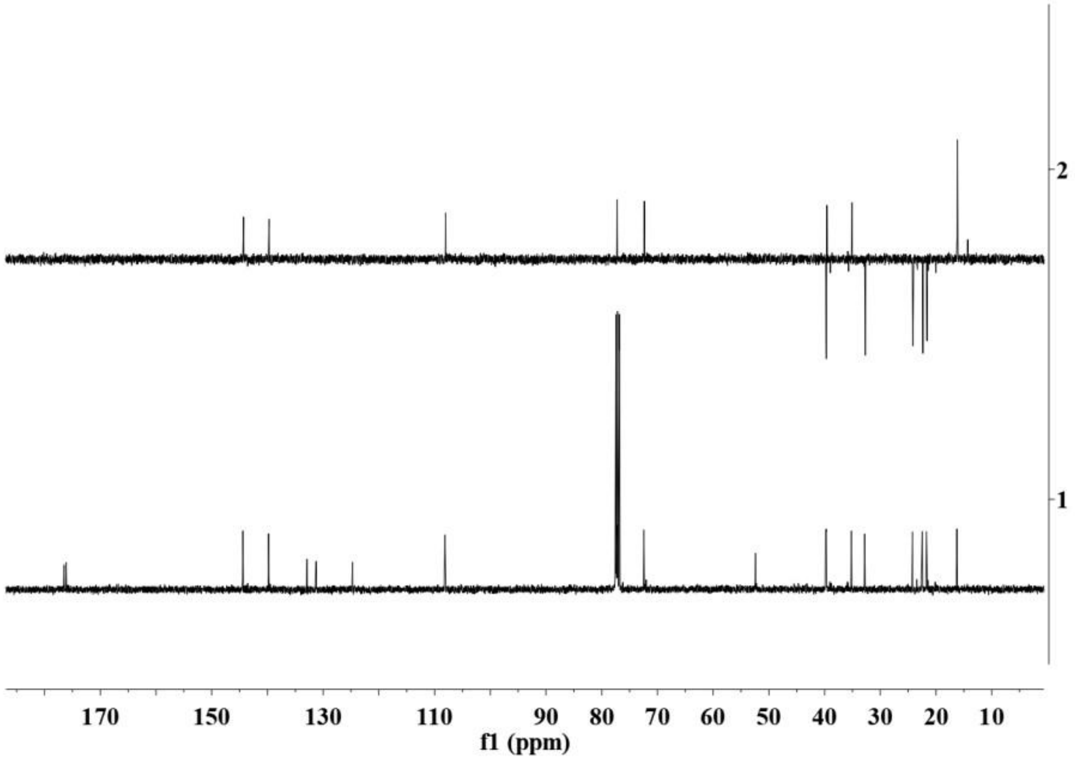

Figure S89. DEPT-135 spectrum of **12** in $CDCl_3$ (100MHz)
